# Technical workflows for hyperspectral plant image assessment and processing on the greenhouse and laboratory scale

**DOI:** 10.1093/gigascience/giaa090

**Published:** 2020-08-20

**Authors:** Stefan Paulus, Anne-Katrin Mahlein

**Affiliations:** Institute of Sugar Beet Research, Holtenser Landstr. 77, 37079 Göttingen, Germany; Institute of Sugar Beet Research, Holtenser Landstr. 77, 37079 Göttingen, Germany

**Keywords:** plant phenotyping, camera calibration, machine learning, hyperspectral signature, hyperspectral

## Abstract

**Background:**

The use of hyperspectral cameras is well established in the field of plant phenotyping, especially as a part of high-throughput routines in greenhouses. Nevertheless, the workflows used differ depending on the applied camera, the plants being imaged, the experience of the users, and the measurement set-up.

**Results:**

This review describes a general workflow for the assessment and processing of hyperspectral plant data at greenhouse and laboratory scale. Aiming at a detailed description of possible error sources, a comprehensive literature review of possibilities to overcome these errors and influences is provided. The processing of hyperspectral data of plants starting from the hardware sensor calibration, the software processing steps to overcome sensor inaccuracies, and the preparation for machine learning is shown and described in detail. Furthermore, plant traits extracted from spectral hypercubes are categorized to standardize the terms used when describing hyperspectral traits in plant phenotyping. A scientific data perspective is introduced covering information for canopy, single organs, plant development, and also combined traits coming from spectral and 3D measuring devices.

**Conclusions:**

This publication provides a structured overview on implementing hyperspectral imaging into biological studies at greenhouse and laboratory scale. Workflows have been categorized to define a trait-level scale according to their metrological level and the processing complexity. A general workflow is shown to outline procedures and requirements to provide fully calibrated data of the highest quality. This is essential for differentiation of the smallest changes from hyperspectral reflectance of plants, to track and trace hyperspectral development as an answer to biotic or abiotic stresses.

## Background

During recent years, hyperspectral sensing of plants has developed as a valuable tool for plant phenotyping [1] [2]. The principle of hyperspectral imaging (HSI) is based on the fact that all materials reflect electromagnetic energy in prominent patterns and specific wavelength owing to differences in their chemical composition, inner physical structure, and surface properties. This signal is characterized by measuring hundreds of narrow bands within the electromagnetic spectrum [3]. Spectroscopy is defined as the method of acquiring and explaining the hyperspectral characteristics of an object regarding light intensity emitted, reflected, or transmitted from molecules at different wavelengths to provide a precise fingerprint of an object. HSI combines spectral and spatial information in a manner similar to that of a digital camera [4]. HSI extends the measurable spectral range from the visible (RGB camera) to the near-infrared (NIR) range and samples the spectrum in a large number of narrow bands (>20 bands). If only a few (<20) spectral bands are sampled, the literature defines this as multispectral. Compared with spectroscopy, which measures the same spectral area, HSI is able to measure spectral and spatial information in an image, which enables a more detailed analysis of the object.

Hyperspectral cameras have become affordable during recent years. Unlike RGB cameras that image the visible spectrum ($400\!-\!700\, \mathrm{nm}$), this area is extended by the ultraviolet (UV, $200\!-\!400\, \mathrm{nm}$) [5]), the NIR ($700\!-\!1000\, \mathrm{nm}$) [6], or even the short-wave infrared spectrum ($1000\!-\!2500\, \mathrm{nm}$) [7]. This is highly interesting for plant science because many plant traits and biophysiological processes can be traced beyond the visible spectral range [8]. HSI of plants has been used to measure plant tissue characteristics [9] and to detect abiotic stresses [10] or plant diseases [11] among others.

Typically, laboratory workflows differ in their use of cameras, measurement set-ups, and data handling such as calibration, smoothing, and segmentation. There are several hardware calibration steps to understand and execute, starting from the camera pixel position mapping to the proper wavelength, the correction of the camera and lens distortion, to the correction of the 3D set-up when measuring upper and lower leaves of a plant. Thus, the introduction of a standardized workflow of hyperspectral image processing is needed to enable the comparison of results from different laboratories regarding their hyperspectral analysis.

To introduce HSI as a state-of-the-art tool for plant phenotyping, we present a literature overview showing the different biological objectives that hyperspectral sensors are used for in the laboratory and at the greenhouse scale. The overview comprises stress detection, disease classification, and a link to molecular analysis (quantitative trait locus [QTL] analysis). All found use-cases are grouped by the introduced trait level description.

The following section introduces techniques to overcome different impairments on the measured spectrum coming from the experimental set-up, the sensor, the role of illumination, and the challenges when measuring complex plants with plant-specific optical properties. The complete workflow from sensor adjustment, correction, calibration, and segmentation to the extraction of hyperspectral plant traits and to a deeper analysis using routines of machine learning (ML) to extract biological information is described.

The application part describes the different aspects of plant traits based on HSI. Finally, a level description model is introduced from the perspective of a data scientist. It describes the increase of complexity in data acquisition and data handling when switching from an averaged spectrum of the plant canopy to an organ-specific spectrum to spectral development in time course to multi-sensor plant models. The latter is needed for the geometrical correction of the spectral data.

## HSI: A Tool for Plant Screening

A comprehensive literature review shows examples for hyperspectral application from detection of biotic stresses such as diseases or viruses and abiotic stresses such as heavy metal or cold exposure and extraction of plant traits such as biochemical traits or leaf water content at greenhouse and laboratory scale. Table 1 emphasizes different use-cases from plant science, where HSI cameras were used to differentiate between different situations.

**Table 1:** Overview of a representative selection of HSI use-cases in plant science at the greenhouse and laboratory scale

Purpose	Group	Plant	Method	Trait level	Target	Reference
Detection of impurities in seeds	Traits	Wheat, spelt, barley	SVM	TL 1	Classification	[17]
Insect damage detection	Biotic stress	Soybean	SVDD	TL 1	Classification	[18]
Cold stress detection	Abiotic stress	Maize	CNN	TL 1	Regression	[19]
Heavy metal stress detection	Abiotic stress	Rice	SVM	TL 1	Classification	[12]
Germination detection	Traits	Trees	LDA	TL 1	Classification	[20]
Virus detection	Biotic stress	Tomato, tobacco	SVM	TL 1	Classification	[21]
Weed resistance analysis	Traits	Amaranth	FLDA	TL 1	Classification	[22]
pH-value determination	Traits	Rice and water hyacinth	PLS and NN	TL 1	Regression	[23]
Nitrogen concentration	Traits	Oilseed rape	SAE and FNN	TL 1	Regression	[24]
Leaf water content	Traits	Maize	PLSR	TL 1	Regression	[14]
Disease detection	Biotic stress	Sugar beet	ANN, DT, SVM	TL 2	Classification	[25]
Disease resistance and QTL analysis	Biotic stress	Sugar beet	SAM	TL 2 and 3	Classification	[26]
Disease development	Biotic stress	Wheat	DT	TL 2 and 3	Classification	[27]
Biomass and biofuel potential	Traits	Maize	SDA	TL 3	Classification	[28]
Water stress detection	Abiotic stress	Tomato	DT	TL 3	Classification	[13]
Salt stress detection	Abiotic stress	Wheat	SiVm	TL 3	Classification	[29]
Biochemical trait analysis	Traits	Maize, soybean	PLSR	TL 3	Regression	[15]
Detection of plant communication	Traits	Maize	LDA	TL 3	Classification	[30]
Disease forecast	Biotic stress	Barley	GAN	TL 3	Classification	[31]
Disease early detection	Biotic stress	Sugar beet	SVM, PLS, DT	TL 3	Classification	[32]
Disease differentiation	Biotic stress	Cucumber	SDA	TL 4	Classification	[33]
Disease detection	Biotic stress	Sugar beet	SVM	TL 4	Classification	[34]

HSI is widely used for detection of biotic and abiotic stresses as well as for trait description. Traits are categorized by a complexity description starting from trait level (TL) 1 (TL1, whole plant) to TL2, organ-specific traits; TL 3, time series; and TL4, multi-sensor traits. ANN: artificial neural network; CNN: convolutional neural network; DT: decision tree; FLDA: Fisher linear discriminant analysis; FNN: fully connected neural network; GAN: generative adversarial network; NN: neural network; PLS: partial least squares; PLSR: partial least squares regression; SAE: stacked auto encoder; SDA: stepwise discriminant analysis; SiVm: SiVm: simplex volume maximization; SVDD: support vector data descriptor; SVM: support vector machines.

In Table 1 hyperspectral data are grouped by TL, which categorizes traits by complexity, starting from simple image analysis (TL 1) to organ identification (TL 2), to time series (TL 3), and to a final multi-sensor data acquisition (TL 4). HSI is shown to be used for classification and regression problems across all 4 TLs. A closer introduction into these phenotypic trait levels can be found below in the text.

Three main groups can be identified including (i) detection and quantification of biotic stresses such as disease [11], (ii) detection and quantification of abiotic stresses such as heavy metal exposure [12] or water stress [13], and (iii) extraction of plant traits to describe water content [14] or biochemical traits [15].

Thus, HSI is widely used for different aspects of plant screening and can be depicted to be a state-of-the-art tool for plant phenotyping.

## Data Acquisition and Processing

Hyperspectral systems and resulting data will vary owing to many factors, including camera characteristics, experimental set-up, calibration, environmental characteristics, and data processing. This leads to inconsistencies regarding the data quality and the validity of results. This increases the difficulty of comparing data from different sensors. Multiple steps are needed to acquire valid physical reflectance data, starting from the sensor wavelength calibration to the instrument function, the radiometric calibration, and spectral and pixel binning.

The goal of calibration is to standardize the spectral axis, to determine whether the sensor is working properly, to provide the accuracy of the extracted data (sensor + processing), to validate the credibility and to quantify the instrument errors, accuracy, and reproducibility under different operating conditions [4].

**Figure 1:**
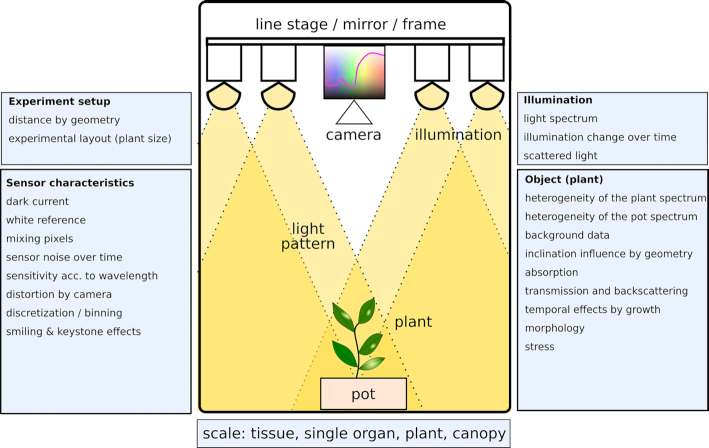
**Influences on the measured spectrum of a plant**. The four main sources of influence are the experimental set-up—the way the camera is mounted, the distance to the plant and so forth; the light spectrum and focus; the object of interest with its absorbing and transmitting properties when imaging plants; and the sensor, in particular the dark and white referencing, its noise and sensitivity, distortion, discretization, and binning.



Four categories of factors that influence the measured spectrum of plants can be defined (see Fig. 1): (i) the experimental set-up including the optical configuration; (ii) the sensor characteristics including sensor offset, noise, and sensitivity behaviour and distortion effects [16]; (iii) the illumination effects from the light source when using active illumination or the surrounding light when using environmental light; and (iv) the object and its properties. Plant object properties means spectral variability due to differences in genotypes, plant organs and materials within the image such as pot and background data; inclination influence due to the architecture of plants; absorption, transmission, and backscattering due to plant tissue properties; and temporal variation due to growth.

#### Camera characteristics

**Figure 2:**
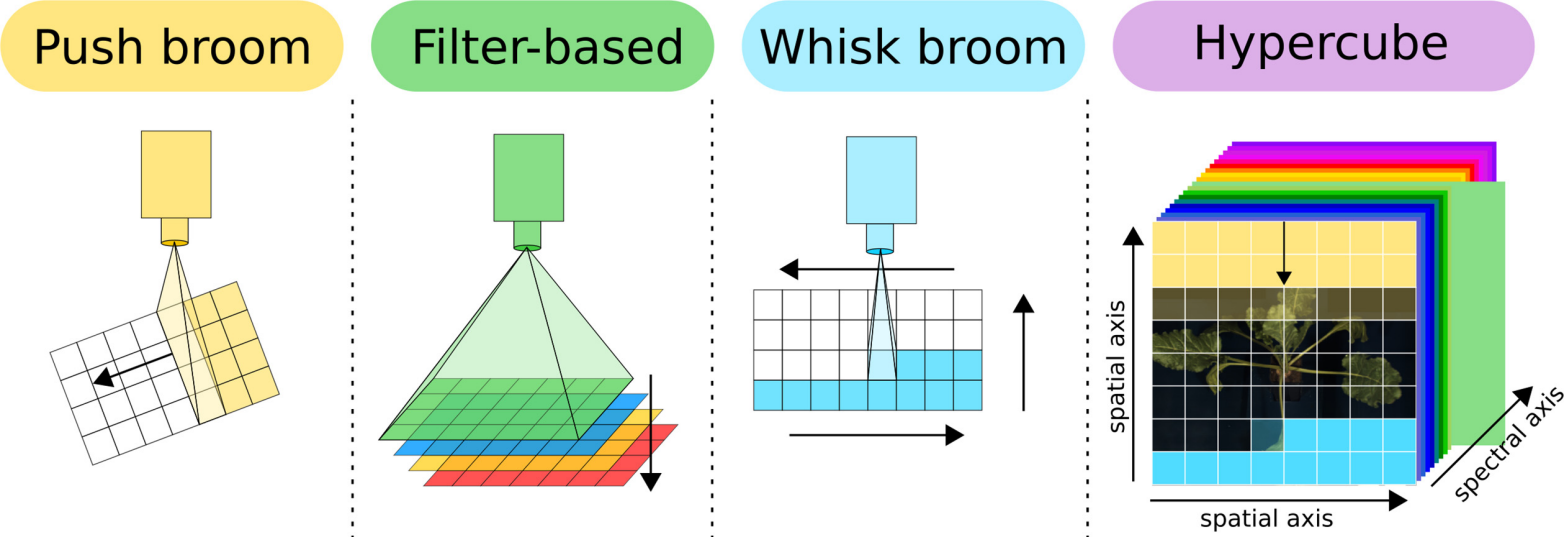
**Overview of common HSI techniques:** 3 different HSI set-ups are commonly used. Push broom cameras (yellow) are line scanners that are moved over the object or alternatively use a mirror; filter-based systems (green) scan single wavelengths according to the filters one after the other; and whisk broom cameras (blue) scan the full spectrum pixel by pixel. All set-ups result in a 3D hypercube (purple) showing 2 spatial axes and 1 spectral axis.

HSI can be performed using 3 different sensor types: the push broom/line scanner, the filter-based sensor set-up, and a whisk broom set-up (see Fig. 2). Push broom cameras scan the region below the sensor in lines and complete the full scan by either moving the sensor [15] or using a mirror that is panned over the object of interest. A filter-based system measures the complete region of interest using different filters either by splitting the scan ray using prisma or by using a combined filter pattern. Whisk broom sensors measure the full spectral range pixel by pixel similar to a spectrometer that is moved over the region of interest. All 3 set-ups result in a 3D hypercube showing 2 spatial axes and 1 spectral axis.

Whisk broom sensors have more moving parts and thus are likely to wear out. Push broom cameras have fewer moving parts but need high-quality calibration because the different regions of the chip can show different sensitivity, which can result in stripes within the data cube. Filter-based systems are commonly restricted by the number of filters and provide less spectral resolution. Currently most state-of-the-art plant phenotyping centers use push broom line scanners.

#### Measurement set-up

Choosing the right camera for a sensor set-up has to take into account the point of interest, side view or top view, depending on whether a single image from the top is sufficient or multiple images obtained by rotating the plant are needed. Other factors to take into account include the spectral region of interest depending on the camera chip (silicon for $380\!-\!1,000\, \mathrm{nm}$, indium-gallium-arsenide for $1,000\!-\!2,500\, \mathrm{nm}$), the focal length, the minimum working distance, the maximum resolution resulting from sensor height and plant height, the focused signal-to-noise ratio, dynamic range, spectral and spatial resolution, pixel size, frame rate, lenses, and operating temperature [35]. In general, the field of view should cover the complete plant from small seedlings to the bigger plants in a time-series experiment. This is accompanied by a periodical adaption of the focal plane because the plant height is changing due to plant development. Here the desired resolution has to be considered because the ratio between plant pixels and background pixels is changing continuously. For reference panels the options are a permanent reference measurement after each plant if a box design is used, referencing within the measurable volume at the same height as the majority of the plant pixels, or a periodical referencing along the scan axis when using a measurement set-up at a longer line stage. More information about reference panels can be found in the section “Data preprocessing—reflectance retrieval.”

#### Illumination for measuring

Illumination is essential for HSI, but not every light source can be used. The use of passive light such as sunlight, which is available outdoors and in greenhouses, is preferred. Some types of greenhouse glass can alter the sunlight spectrum if a coating or special glass is used. Active light sources need a closer consideration. Tungsten halogen lamps are a broad-band emitter ($400\!-\!2,600\, \mathrm{nm}$) and are economically affordable and technically easy to set up. In contrast, gas discharge tubes (fluorescent tubes or uncoated tubes) are not usable because they emit high narrow lines in the spectrum. Nevertheless, deuterium gas discharge can be used for UV-measurement applications and arc sources such as xenon lamps can be used for snapshot cameras. LED lamps can be used depending on the implemented technology and use-case according to the measurement scenario and emitted wavebands [36].

**Figure 3:**
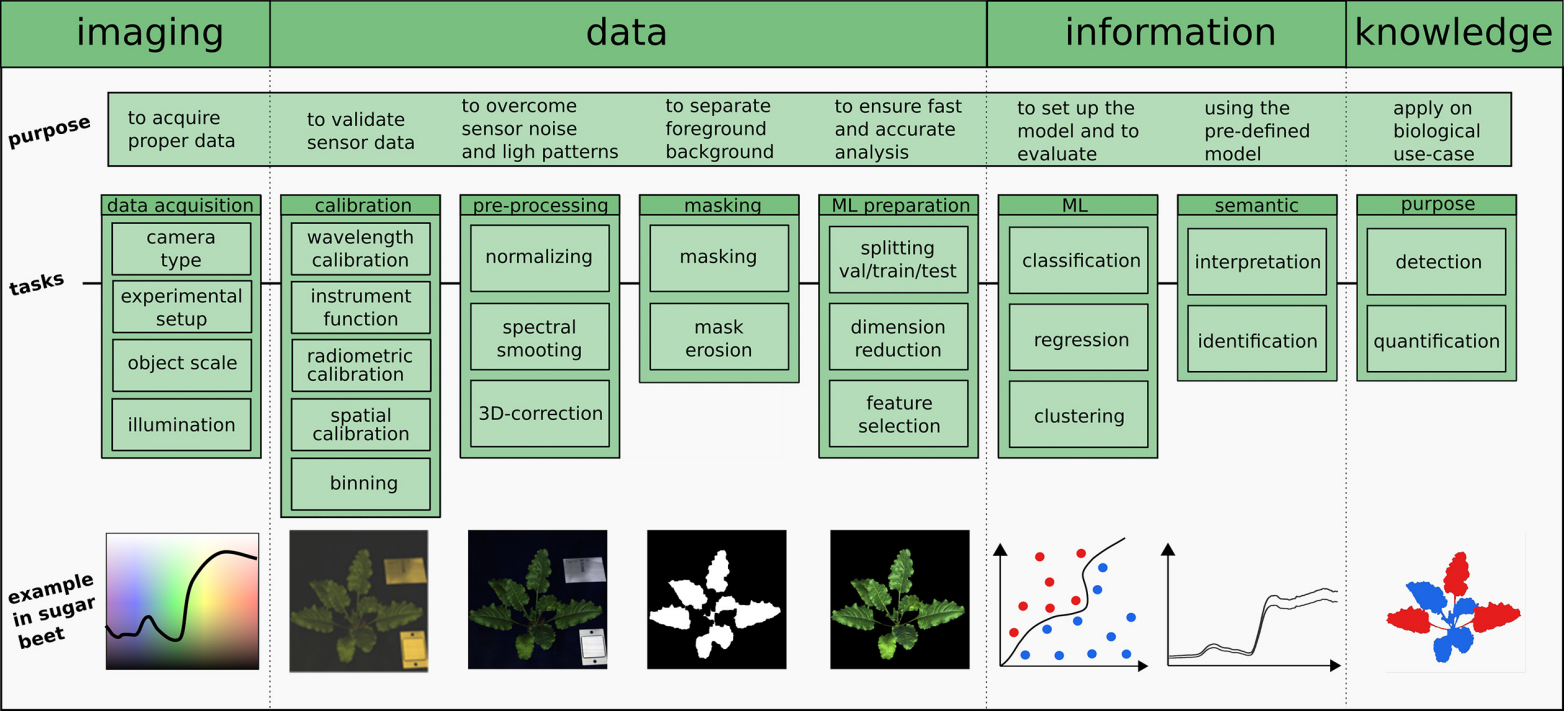
**A generalization of a hyperspectral workflow**. The workflow to extract information from sensor data and to bring it into a biological context to generate knowledge starts with data acquisition, hardware calibration, a proper normalization step, data pre-processing, and masking to focus on the object of interest—the plant—and to eliminate background (e.g., plant pot and stabilization sticks). Depending on the experiment set-up, data and the analysis type have to be divided into validation (val), training, and test dataset to train a model and then evaluate it on the test data. This is followed by the result interpretation and identification of diseases, stresses, or other properties of the plants. Vertical dashed lines describe in a general way the transition between the imaging process, the processing of the data, the generation of information, and interpretation of knowledge.

To acquire a proper data cube different calibration routines are needed to ensure highly accurate reflectance values. Fig. 3 shows a generalized processing pipeline for hyperspectral cubes for the demands of plant imaging in greenhouses and laboratories as is common for plant phenotyping.

#### Wavelength calibration—from pixel to wavelength

When a pushbroom sensor is used, 1 dimension of the detector represents the spatial information of the lines of the target. The other dimension represents the full spectrum of a single line of pixels. The wavelength calibration describes the comparison of measured spectral values with known values [37] and, consequently, the mapping of the dispersed geometric axis to wavelength in nanometers.

Calibration is needed after manufacturing and after any physical changes to the optical path [38]. Wavelength calibration is obtained by exposing the optical system to a calibration light source or sources. Three aspects are critical for obtaining a proper wavelength calibration including (i) the selection of the calibration light, (ii) the determination of the center of characteristic peaks, and (iii) polynomial fitting to the data [39]. The calibration light source(s) should cover the wavelength range to be calibrated. Wavelength calibration light sources emit atomic emission lines of known wavelengths. A polynomial fit of the geometric position of the atomic emission lines on the chip and the known wavelength is conducted. This step is usually performed primarily by the manufacturer and enables the spectral axis to be displayed in units of wavelength (nanometers).

#### Instrument function/point-spread function—overcoming spectral distortion

Measurements of any optical device can be described as a convolution of the original data with the appropriate transfer function of the sensor and optical set-up. This convolution is characterized as a (spectral and spatial) blurring or smearing of the data [40]. The terms “instrument function" and “point-spread function" are both used to describe this convolution. The term “point-spread function" typically refers to the spatial convolution. The term “instrument function" refers to the convolution in the spectral domain. Both terms define the highest possible spectral and spatial resolution. Effects resulting from the point-spread function are described in the following paragraphs. In contrast to spatial distortions the (spectral) instrument function is typically not corrected.

#### Spatial calibration—overcoming spatial distortion

Similar to 2D-RGB cameras, which come with barrel and pillow distortion [41], the images of a hyperspectral line scanner tend to show similar effects called smile and keystone effects. Smile is the curvature distortion of the horizontal spectra lines [16], or a shift in wavelength in the spectral domain [42]. Keystone is the distortion of the focal plane rectangle into a trapezoid [16], or a band-to-band misregistration [42]. These effects can be corrected using geometric control points [16]. A spatial calibration of the hyperspectral cube describes the character of the spatial mapping process. This process results in a rectified image. Not all manufacturers provide this calibration by default.

#### Radiometric calibration—from counts to a physical unit

Owing to differences in quantum efficiency of the detector and varying efficiency of the grating and other optical components (e.g., lenses), measurements using different optical systems of the same object under the same illumination conditions may not be identical [38]. The data level is influenced by sensor characteristics, atmospheric conditions, and surface properties of the plants. On the most basic level cameras return their measurement values as digital numbers. To correct for such instrument-related variability within these returned digital numbers, radiometric calibration of the measurement device or white referencing is needed. Radiometric calibration transforms these digital numbers to radiance values. Radiance depicts the physical measurement of the spectral power flux emitted, received, transmitted, or reflected by an object per unit solid angle and projected area. It uses an integrating sphere to measure the calibration coefficients for each wavelength band (pixel) [43].

The camera digital output is mapped to a physical quantity (radiance) using a certified spectral transfer standard (integrating sphere plus calibrated emitter). Thus, radiometric calibration accounts for the spectral variation of the external lens system, internal optics, sensor, and dispersive elements (grating and filter). Radiance values are typically used in high-altitude/long-distance measurement scenarios (plane- or satellite-based measurements). Radiometric calibration does not account for a potential active illumination light source or atmospheric absorption between the object under study and the camera system, as well as surface properties of the specimen. It corrects for the camera and optics spectrally varying efficiency.

Radiance data can be converted to reflectance data if the irradiation source is known or measured [44]. In many applications absolute radiometric calibration and the corresponding radiance data are not required. Often, it is preferred to use reflectance data rather than radiance data. In contrast to radiance data, which involve an absolute calibration, reflectance data do not require absolute calibration. A relative spectral calibration to correct for the spectrally varying system efficiency using a simple white reference and dark offset subtraction is sufficient for reflectance measurements. Reflectance data are corrected for camera effects, atmospheric conditions, and lighting effects, so only the surface properties of the measured object remain.

#### Spectral and spatial binning—reducing the noise level

To acquire a high retrieval accuracy within the acquired data a high signal-to-noise ratio (SNR) is required. SNR is the ratio of the radiance measured to the noise created by the detector and instrument electronics [4]. This ratio can be increased by combining spectral image information along the spectral axis (spectral binning) or by integrating the neighbour pixels (spatial binning) [35]. It was shown that binning along the spectral axis using just a few neighbours reduces the (spectral) image size in favour of an enhanced SNR [45]. Nevertheless, the lowest SNR is usually found at the beginning and end of the measurable range of a sensor. A common step to deal with this area is simply cutting the first and last few spectral bands of the sensor [36].

In general, wavelengths next to each other are highly correlated [46]. Thus it can be stated that a limited spectral binning will not affect the informative value of the remaining spectrum.

Binning can be performed directly at the camera internal hardware (hardware binning) or by processing software when loading the data cube (software binning). In general, hardware binning results in less noise than software binning because the sensor signal is directly merged in the camera prior to analog-digital conversion. In hardware binning, this step has to be performed before any calibration. In software binning, it is the first step in the pre-processing right after the hardware calibration steps.

### Data pre-processing

Pre-processing can be initiated after hardware calibration and measurement validation. A standardized process is needed to compare measurements from different timepoints and from different measurement set-ups. The pre-procesing steps include the reflectance retrieval, the spectral smoothing and 3D correction, masking of the object of interest, data splitting, dimension reduction, and feature selection for ML.

#### Reflectance retrieval—overcoming the light source influence

To enable comparable measurements for time series within the same measurement set-up, between different sensor set-ups, or under different illumination conditions it is necessary to normalize the data cube according to the maximum and minimum reflectance intensity. Therefore the dark image is captured by recording the hypercube with a lid on the camera or a closed shutter. This dark data cube describes the lowest possible sensor signal. Right after this the white reference spectrum is acquired using a spectrally known reference target. Most often highly reflective materials such as barium sulfate (available from such suppliers as SphereOptics or Labsphere)act as a reference. Alternatively the use of materials with a known spectral reflectance across the entire spectral range is established as a standard procedure. Here black, dark, and light gray objects can be measured with a point spectrometer to get a known reflectance value. When sharing datasets the reference spectral characteristics should be provided as metadata to ensure reusability and compatability. For performing the normalization step the object scan, the dark current scan, and the reference panel scan are needed. The normalization step can be described by Equation 1:

(1) \begin{equation*} I_{\mathrm{Norm}} = \frac{\mathrm{cube}_{O} - \mathrm{cube}^{D}_{O}}{\mathrm{cube}^{W}_{R} - \mathrm{cube}^{D}_{R}}. \end{equation*}

Equation 1 has already been described in the literature [3, 4]. The numerator describes the subtraction of the measured object cube cube_*O*_ and the associated dark current $\mathrm{cube}^{D}_{O}$, while the denominator describes the subtraction of the white reference measurement $\mathrm{cube}^{W}_{R}$ and the associated dark reference $\mathrm{cube}^{D}_{R}$. An important feature of Equation 1 is the reduction of non-uniformity caused by either the imaging chip, the illumination, or the measurement situation (e.g., box).

For measurements in a greenhouse with a variable environment such as a change in light conditions, or when measuring time series or measurements that cover a large area, it is recommended to use multiple targets or periodic recalibration of the sensor set-up.

#### Spectral smoothing—dealing with peaks and spectral outliers

Based on the assumption that the plant spectrum is smooth and that peaks covering just 1 or 2 bands within the spectrum are the result of outliers and noise, the use of soft smoothing algorithms is valid. The Savitzky-Golay smoothing algorithm [47] is the most established one for hyperspectral data. Bohnenkamp et al. [48] showed the applicability for use of 15 centered points and a third-degree polynomial for a Specim FX10 camera providing 220 bands within $400\!-\!1,000\, \mathrm{nm}$. Furthermore multiplicative signal correction [49] and standard normal variate [50] are well-established routines for signal correction.

#### 3D Correction—correcting the influence of the sensor-object distance

The measured reflectance on the detector depends on the reflected light intensity and the distance between sensor and reflection point on the object/plant. For measuring a plant with upper and lower leaves, the distance to the sensor is different for each leaf. This results in differences in the measured intensity. Some publications show the normalization of the spatial distance [22, 51]. A prerequisite for this is the integration of a 3D measuring device in the measuring set-up (e.g., laser scanner, ultrasound). Depending on the distance, the corrected cube contains equal reflectance values for similar surfaces although the distance to the camera is different by using pixel-wise distance normalization.

#### Segmentation masking

Image segmentation is used to partition an image into meaningful parts that have similar features and properties [52]. For the demands of plant phenotyping this usually means the separation from plant and background pixels. This is mostly based on simple vegetation indices or thresholds using a specific wavelength [53]. Further segmentation such as the identification of single leaves or the detection of disease symptoms is focused on later in the workflow pipeline as ML methods are used to tackle this problem.

After masking, the transition between foreground and background is very sharp. Pixels at this transition include parts of both classes and are depicted as “mixed pixels.” To overcome the influence of these pixels on the analysis result, these pixels have to be removed. The literature shows that the use of erosion as a binary image processing technique is efficient. A filter element with a size of 3 × 3 pixels is used to shrink the region of the foreground [54]. An adverse effect related to the reduction of foreground data is the possibility of losing important information that can be used to enhance the data quality.

#### Preparation for ML

Up to this point the data cube consists of hundreds of spectral bands. To detect the specific wavelength that includes the biggest impact for the question of interest, ML is needed. This is also important for a later transfer to multispectral cameras with fewer spectral bands but with the opportunity to measure in high throughput on the field scale.

To prepare the data for use in a common ML routine, using supervised classification approaches, the dataset is split into 3 subgroups including the same distribution of groups within the 3 sets. That means the ratio between the included groups is similar. Set 1 is called the training set and is used to calculate the model of the ML method such as support vector machines (SVM) or decision trees (DT). Set 2 is called the validation dataset and is used for model hyper-parameter tuning. The third set is called the test set and is used to evaluate the performance of the developed model and to calculate model accuracy. The size of the groups differs with respect to the number of available samples. A repeated cross-validation using different splits of the dataset (test and training) is recommended. Dimensionality reduction methods can decrease spectral redundancy and reduce data volume within the dataset. Common techniques are principal component analysis [55], feature selection using recursive feature elimination [56], ReliefF [57], or correlation-based feature selection [58].

### Data analysis and interpretation

#### Hyperspectral traits

**Figure 4:**
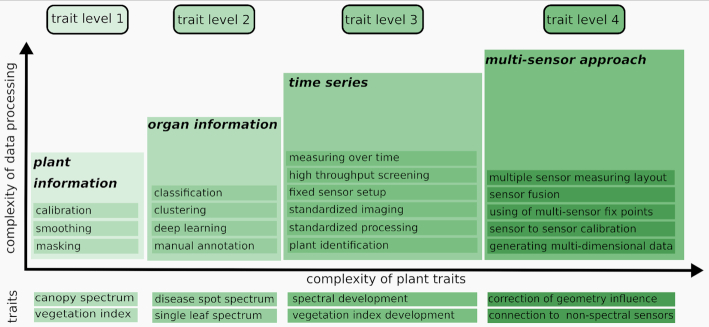
**A general trait visualization**. Plant traits are parameters that describe the hyperspectral properties of the plant tissue. Nevertheless, these traits can be grouped according to the effort that is needed for their extraction. First-level traits (TL 1) describe the spectrum of the whole canopy. By using a classification based on a ML algorithm it is possible to identify spectra of single organs (TL 2). By taking measurements over time the development of these spectra can be visualized (TL 3), and by using further sensors it is possible to reduce geometrical effects based on a particular sensor by means of fusion of sensor data (TL 4).

Hyperspectral traits can be categorized into different groups, depending of the focus of the data. If the data are coming from a single plant (TL 1), the data cube can be used to derive very low resolution information about the plant such as the plant canopy [59]. If the data cube is segmented into regions including single leaves, disease symptoms, or spatially confined areas (ROI, TL 2), these regions can be compared. This is commonly done by classification on a pixel (single spectrum) level [60]. Time-series measurements are essential for accurate capturing of developing disease symptoms. This leads to the development of hyperspectral dynamics over time (TL 3) [27, 48]. Hyperspectral data cubes are affected by distance and the inclination of the measured object, so the hyperspectral information needs to be corrected for these factors. This can be done by modeling the measurement set-up and the errors that occur. It necessitates the use of an accompanying sensor to measure the object geometry such as a 3D laser scanner [61, 62] and fuse the data for a complete 3D-hyperspectral data model that enables detection of plant disease within a corrected spectrum [34]. An overview of these traits, prerequisites, and applications is shown in Fig. 4.

#### Machine learning

For data analysis and ML, the tasks can be divided into supervised methods and unsupervised methods. Supervised methods require a known target value and therefore labelled data to train a model. Within the supervised learning methods, methods can be grouped by their target. If the output is a label as an affiliation to a group and thus is categorical, the method is called classification. Prominent routines for supervised classification are SVM, DT, and neural network (NN) architectures. A similar approach using labelled data is regression, where the output does not predict a group but a numeric value. Known methods for this scenario are support vector regression (SVR), DT, and NN.

A special case of ML is deep learning (DL). DL allows computational models that are composed of multiple processing layers to learn representations of data with multiple levels of abstraction. It also describes an algorithm allowing raw data as input and automatically discovers a representation, consisting of multiple non-linear modules, for detection or classification [63]. In contrast to SVM or DT approaches, DL is based on NN architectures and depends on huge labelled datasets for training. DL approaches have been widely used on RGB images for the demands of plant phenotyping as a classification of root tips, shoots, and leaves [64,65] and can be characterized as state of the art. During recent years, hyperspectral applications have been used more widely. Different types of DL approaches have been used for plant disease [66] or stress detection [67].

Usually the results of a classification are presented by a confusion matrix, which indicates for a specific trained model the resulting classification of the test dataset regarding true-positive, false-positive, false-negative, and true-negative results. This compares the predicted values to the true values.

Unsupervised approaches do not need labelled data and try to detect patterns within the data. Clustering approaches such as *k*-means shift manual work from model generation to cluster interpretation because it becomes the task of the scientist to give a semantic interpretation to the clustered datasets. The clustering of hyperspectral datasets has been successfully shown for the detection of drought exposure in maize [68].

### Challenges and limitations

HSI faces many challenges regarding sensor set-up, illumination, data processing, and plant-specific characteristics. This starts with the measuring set-up, where the sensor, illumination, and the object distance have to be adapted to the plant size to optimize reflectance results. Including both extrema within 1 measuring set-up can cause problems in illumination, image resolution, and chip intensity.

When HSI is extended to the UV range between 200 and 400 nm, plants can be damaged by the harmful properties of illumination in this spectral region [5]. Further evaluation of the effects of light exposure on the study subjects is recommended because plant properties such as architecture, tissue composition, and wax layer differ between species.

Surface geometry has a remarkable effect on the measured spectrum. Behmann et al. [7] found a correlation between normalized difference vegetation index and surface inclination. Thus this effect has to at least be taken into account or ideally be corrected. This emphasizes the need for imaging set-ups to include different sensors for geometry and reflectance.

The workflow proposed is not transferable to field conditions, which necessitate a different experimental set-up to ensure high-quality hyperspectral measurements [69].

High-throughput imaging set-ups [14] combine hyperspectral cameras with high-frequency imaging, which leads to complex datasets independent of the scale [70]. This emphasizes the need for reliable, stable, and efficient algorithms and high-end computational machines to process the data cubes. Image analysis and interpretation is the key plant phenotyping bottleneck [71].

## Conclusion

HSI is a well-established tool for plant phenotyping in greenhouses. However, each laboratory uses a specialized workflow for data assessment, processing, and handling, which makes the data individually valid but difficult to compare.

This study introduces a generalized workflow for handling HSI data for greenhouses and laboratories. It includes calibration, reflectance retrieval, data smoothing, masking, and preparation for use in an ML routine.

This workflow includes hardware-based calibration steps, as well as software-based processing. Furthermore, a general definition for hyperspectral traits is introduced to establish a level system starting from traits for the whole plant, to traits for single organs, traits describing temporal development, and traits that are based on the measurements of different sensors. A literature overview of the use of HSI and ML demonstrates the different application areas for plant measurement in agriculture together with the ML method and plant material used. This review offers a standardized protocol for raw data processing and how plant traits can be categorized owing to their complexity regarding effort in data processing and derivable traits.

## Abbreviations

ANN: artificial neural network; CNN: convolutional neural network; DC: dark current; DT: decision tree; FLDA: Fisher linear discriminant analysis; FNN: fully connected neural network; GAN: generative adversarial network; HSI: hyperspectral imaging; ML: machine learning; NIR: near-infrared; NN: neural network; PLSR: partial least squares regression; QTL: quantitative trait locus; RGB: red, green, blue, digital camera sensor; SAE: stacked auto encoder; SAM: spectral angle mapper; SDA: stepwise discriminant analysis; SNR: signal-to-noise ratio; SiVm: simplex volume maximization; SVDD: support vector data descriptor; SVM: support vector machines; TL: trait level; VNIR: visual + infrared spectrum; WR: white reference.

## Competing Interests

The authors declare that they have no competing interests.

## Funding

This study was partially funded by the Deutsche Forschungsgemeinschaft (DFG, German Research Foundation) under Germany's Excellence Strategy—EXC 2070-390732324. It was also supported by Bayer AG–Crop Science.

## Authors' Contributions

Both authors designed the research. A.K.M. supervised the project. Both authors wrote the manuscript, prepared the figures, studied the literature, and read and approved the final version of the article.

## Supplementary Material

giaa090_GIGA-D-20-00091_Original_Submission

giaa090_GIGA-D-20-00091_Revision_1

giaa090_GIGA-D-20-00091_Revision_2

giaa090_Response_to_Reviewer_Comments_Original_Submission

giaa090_Response_to_Reviewer_Comments_Revision_1

giaa090_Reviewer_1_Report_Original_Submission Yu Jiang -- 4/9/2020 Reviewed

giaa090_Reviewer_2_Report_Original_Submission Yufeng Ge -- 4/15/2020 Reviewed

giaa090_Reviewer_2_Report_Revision_1 Yufeng Ge -- 7/23/2020 Reviewed

giaa090_Reviewer_3_Report_Original_Submission Susan Meerdink -- 4/23/2020 Reviewed

giaa090_Reviewer_3_Report_Revision_1 Susan Meerdink -- 7/6/2020 Reviewed
