# Supplementary material for: Technical workflows for hyperspectral plant image assessment and processing on the greenhouse and laboratory scale
Source: Gigascience. 2020 Aug 20;9(8):giaa090. doi: 10.1093/gigascience/giaa090 (PMC7439585; doi:10.1093/gigascience/giaa090)
Supplement: giaa090_GIGA-D-20-00091_Original_Submission [file giaa090_giga-d-20-00091_original_submission.pdf]

GigaScience
Technical workflows for hyperspectral plant image assessment and processing on the greenhouse and laboratory scale
--Manuscript Draft--

**Manuscript Number:**	GIGA-D-20-00091
**Full Title:**	Technical workflows for hyperspectral plant image assessment and processing on the greenhouse and laboratory scale
**Article Type:**	Review
**Funding Information:**	Deutsche Forschungsgemeinschaft (EXC 2070 - 39073232) — Prof. Dr. Anne-Katrin Mahlein<br>Bayer CropScience — Prof. Dr. Anne-Katrin Mahlein
**Abstract:**	Using hyperspectral cameras is well established in the field of plant phenotyping, especially when using high throughput routines in greenhouses. Nevertheless, the used workflows differ depending on the applied camera, the imaged plants, the experience of the users and the measuring setup.<br>This review describes a general workflow for the assessment and the processing of hyperspectral plant data at the greenhouse scale. Aiming at a detailed description of possible error sources, a comprising literature review of possibilities to overcome these errors and influences is provided. The processing of hyperspectral data of plants starting from the hardware sensor calibration, the software processing steps to overcome sensor inaccuracies and the preparation for machine learning is shown and described in detail. Furthermore, plant traits extracted from spectral hypercubes are categorized to standardize the terms used when describing spectral traits in plant phenotyping. Data is introduced from a scientific view on the data for canopy, single organs, plant development and also combined traits coming from spectral and 3D measuring devices.<br><br>This publications provides a structured overview on implementing hyperspectral imaging into biological studies.<br><br>Workflows have been categorized to define a trait level scale according to their metrological level and the processing complexity. A general workflow is shown to outline procedures and requirements to provide fully calibrated data of highest quality. This is essential for differentiation of tiny changes from spectral reflectance of plants, to track and trace spectral development as an answer to biotic or abiotic stresses.
**Corresponding Author:**	Stefan Paulus, Ph.D.<br>Institute of Sugar Beet Research<br>Göttingen, GERMANY
**Corresponding Author Secondary Information:**	
**Corresponding Author's Institution:**	Institute of Sugar Beet Research
**Corresponding Author's Secondary Institution:**	
**First Author:**	Stefan Paulus, Ph.D.
**First Author Secondary Information:**	
**Order of Authors:**	Stefan Paulus, Ph.D.
	Anne-Katrin Mahlein, Professor
**Order of Authors Secondary Information:**	
**Additional Information:**	
**Question**	**Response**
Are you submitting this manuscript to a special series or article collection?	No

Powered by Editorial Manager® and ProduXion Manager® from Aries Systems Corporation

**Experimental design and statistics**<br><br>Full details of the experimental design and statistical methods used should be given in the Methods section, as detailed in our Minimum Standards Reporting Checklist. Information essential to interpreting the data presented should be made available in the figure legends.<br><br>Have you included all the information requested in your manuscript?	Yes
**Resources**<br><br>A description of all resources used, including antibodies, cell lines, animals and software tools, with enough information to allow them to be uniquely identified, should be included in the Methods section. Authors are strongly encouraged to cite Research Resource Identifiers (RRIDs) for antibodies, model organisms and tools, where possible.<br><br>Have you included the information requested as detailed in our Minimum Standards Reporting Checklist?	Yes
**Availability of data and materials**<br><br>All datasets and code on which the conclusions of the paper rely must be either included in your submission or deposited in publicly available repositories (where available and ethically appropriate), referencing such data using a unique identifier in the references and in the "Availability of Data and Materials" section of your manuscript.<br><br>Have you have met the above requirement as detailed in our Minimum Standards Reporting Checklist?	Yes

Powered by Editorial Manager® and ProduXion Manager® from Aries Systems Corporation

Manuscript
Click here to download LaTeX - Main Document (TeX file) main.tex

Warning. The length \marginparwidth is less than 2cm and will most likely cause issues with the appearance of inserted todonotes. The issue can be solved by adding a line like \setlength{\marginparwidth}{2cm} prior to loading the todonotes package.

GigaScience, 2017, 1–11

doi: xx.xxxx/xxxx
Manuscript in Preparation
Review

REVIEW

Technical workflows for hyperspectral plant image assessment and processing on the greenhouse and laboratory scale

Stefan Paulus[1,*] and Anne-Katrin Mahlein[1,†]

[1]Institute of Sugar Beet Research, Holtenser Landstr. 77, 37079 Göttingen, Germany

[*]paulus@ifz-goettingen.de;
[†]mahlein@ifz-goettingen.de

Abstract

Background: Using hyperspectral cameras is well established in the field of plant phenotyping, especially when using high throughput routines in greenhouses. Nevertheless, the used workflows differ depending on the applied camera, the imaged plants, the experience of the users and the measuring setup.
Results: This review describes a general workflow for the assessment and the processing of hyperspectral plant data at the greenhouse scale. Aiming at a detailed description of possible error sources, a comprising literature review of possibilities to overcome these errors and influences is provided. The processing of hyperspectral data of plants starting from the hardware sensor calibration, the software processing steps to overcome sensor inaccuracies and the preparation for machine learning is shown and described in detail.
Furthermore, plant traits extracted from spectral hypercubes are categorized to standardize the terms used when describing spectral traits in plant phenotyping. Data is introduced from a scientific view on the data for canopy, single organs, plant development and also combined traits coming from spectral and 3D measuring devices. **Conclusions** This publications provides a structured overview on implementing hyperspectral imaging into biological studies. Workflows have been categorized to define a trait level scale according to their metrological level and the processing complexity. A general workflow is shown to outline procedures and requirements to provide fully calibrated data of highest quality. This is essential for differentiation of tiny changes from spectral reflectance of plants, to track and trace spectral development as an answer to biotic or abiotic stresses.

Key words: plant phenotyping, camera calibration, machine learning, spectral signature

Background

During the last years, spectral sensing of plants has developed as a valuable tool for plant phenotyping [1] [2]. The principle of hyperspectral imaging (HSI) is based on the fact that all materials reflect electromagnetic energy in prominent patterns and specific wavelength due to difference of their chemical composition and inner physical structure [3]. Spectroscopy is defined as the method of acquiring and explaining the spectral characteristics of an object regarding light intensity emerging from molecules at different wavelengths to provide a precise fingerprint of an object. Spectral imaging combines spatial and temporal information similar to a digital camera [4].

Spectral cameras have become affordable that increase the visible spectrum (400 – 700*nm*, VIS) of RGB-cameras by the ultra-violet (200 – 400*nm*, UV,[5]), the near infrared spectrum (700 – 1000*nm*,NIR, [6]) or even the short wave infrared spectrum (1000 – 2500*nm*, SWIR, [7]). This is highly interesting for plant science as many plant traits and biophysiological processes can be traced beyond the visible spectral range [8]. Reflectance imaging of plants has been related to plant tissue characteristics [9], to detect abiotic stresses [10] or plant dis-

Compiled on: March 27, 2020.
Draft manuscript prepared by the author.

Key Points

- a literature overview is provided to describe aims and scopes of spectral sensing of plants and the different types of analysis methods
- hyperspectral workflows for plant measuring are highly individual and need to be structured for evaluation
- a general workflow for hyperspectral plant phenotyping including camera calibration, segmentation and machine learning analysis is shown
- a level-based trait definition is introduced for canopy, plant organ, time series and sensor fusion

eases [11].

Typically, laboratory workflows differ in their use of cameras, measuring setups and data handling such as calibration, smoothing and segmentation. There are several hardware calibration steps to understand and execute, starting from the camera pixel position mapping to the proper wavelength, the correction of the camera and lens distortion to the correction of the 3D setup when measuring upper and lower leaves of a plant. Thus, a standardized introduction of a workflow of hyperspectral image processing is needed to enable the comparison of results from different laboratories regarding their hyperspectral analysis.

To introduce HSI as a state-of-the-art tool for plant phenotyping a literature overview is presented showing the different biological objectives what hyperspectral sensors are used for in the laboratory and greenhouse scale starting from stress detection and disease classification to a linking to molecular analysis (QTL analysis) grouped by the introduced level-description.

The following paragraph introduced introduces techniques to overcome different impairments on the measured spectrum coming from the experimental setup, the sensor, the role of illumination and the challenges when measuring complex plants with plant specific optical properties. The complete workflow from sensor adjustment, correction, calibration, segmentation to the extraction of spectral plant traits and to a deeper analysis using routines of machine learning (ML) to extract biological information is described.

The application part describes the different aspects of plant traits based on HSI. Finally, a level-description model is introduced from the perspective of a data scientist. It describes the increase of complexity in data acquisition and data handling, when switching from an averaged spectrum of the plant canopy to an organ-specific spectrum to spectral development in time course to multi-sensor plant models. The latter is needed for the geometrical correction of the spectral data.

HSI a tool for plant screening

A comprehensive literature review shows examples for hyperspectral application from biotic stress detection like disease or virus detection, abiotic stress detection like heavy metal or cold stress and plant trait extraction like biochemical traits or leaf water content. Table 1 emphasizes different use-cases from plant science, where hyperspectral imaging cameras were used to differentiate between different situations.

In table 1 hyperspectral data was grouped by trait level which describes the complexity of the traits. Starting from simple image analysis (level 1), to organ identification (level 2), to time series (level 3) and to a final multi-sensor data acquisition (level 4). It is shown that HSI is used for classification and regression problems across all trait levels (1-4). A closer introduction into these phenotypic trait levels can be found below in the text.

Three main groups can be identified including i) detection and quantification of biotic stress like disease detection [11], ii) detection and quantification of abiotic stress like heavy metal [15] or water stress [26] and iii) extraction of plant traits to describe water content [21] or biochemical traits [28].

Thus, HSI is widely used for different aspects of plant screening and can be depicted to be a state-of-the-art tool for plant phenotyping.

Data acquisition and processing

Spectral systems and resulting data differ in the way the camera is calibrated and the data is processed. This leads to inconsistencies regarding the data quality and the validity of results. This increases the difficulty to compare data from different sensors. Multiple steps are needed to acquire valid physical reflectance data starting from the sensor wavelength calibration, the instrument function, the radiometric calibration and spectral and pixel binning.

The goal of calibration is to standardize the spectral axis, determining if the sensor is working properly, providing the accuracy of the extracted data, validate the credibility and quantify the instrument errors, accuracy and reproducibility under different operating conditions [4].

Four categories of factors that influence the measured spectrum of plants can bedefined (see Figure 1). I) the experimental setup including the optical configuration II) the sensor characteristics including sensor offset, noise and sensitivity behaviour and distortion effects [33] III) the illumination effects from the light source when using active illumination or the surrounding light when using environmental light and IV) object and its properties. This causes plant spectrum variability due to differences in genotypes, plant organs, materials within the image such as pot and background data, inclination influence due to the architecture of plants, absorption, transmission and backscattering as plant tissue properties and temporal effects due to growth.

Camera characteristics and measuring setup

Hyperspectral cameras for plant phenotyping often are line scanners (pushbrooms) as this type of sensor is commonly used in plant science or for high throughput analysis as it, unlike snapshot cameras, provides a very high spatial and spectral resolution. These scanners are either moved over the plant or use a mirror for panning over the plant and to produce a full 3D (2D spatial + spectral dimension) hyperspectral cube. Currently state-of-the-art plant phenotyping centers use line scanners for imaging maize lines [34], to detect genotypic differences [35] or to predict the nitrogen content in wheat [36]. The next step, the transfer of these sensor types to the field scale has already been started for tracking the canopy development in cereals [37] or as an open-source and open data project of Terra-Ref [38].

The measuring setup of a hyperspectral line scanner con-

Table 1. Hyperspectral imaging is widely used for detection of biotic and abiotic stresses as well as for trait description. Traits are categorized by a complexity description starting from trait level 1 (TL1, whole plant), trait level 2 (TL2, organ specific traits), trait level 3 (TL 3, time series) and trait level 4 (TL4, multi sensor traits).

purpose	group	plant	method	trait level	target	reference
detection of impurities in seeds	traits	wheat, spelt, barley	SVM	TL 1	classification	[12]
insect damage detection	biotic stress	soybean	SVDD	TL 1	classification	[13]
cold stress detection	abiotic stress	maize	CNN	TL 1	regression	[14]
heavy metal stress detection	abiotic stress	rice	SVM	TL 1	classification	[15]
germination detection	traits	trees	LDA	TL 1	classification	[16]
virus detection	biotic stress	tomato, tobacco	SVM	TL 1	classification	[17]
weed resistance analysis	traits	amaranth	FLDA	TL 1	classification	[18]
ph-value determination	traits	rice & water hyacinth	PLS & NN	TL 1	regression	[19]
nitrogen concentration	traits	oilseed rape	SAE & FNN	TL 1	regression	[20]
leaf water content	traits	maize	PLSR	TL 1	regression	[21]
disease detection	biotic stress	sugar beet	ANN, DT, SVM	TL 2	classification	[22]
disease resistance & QTL analysis	biotic stress	sugar beet	SAM	TL 2&3	classification	[23]
disease development	biotic stress	wheat	DT	TL 2&3	classification	[24]
biomass & biofuel potential	traits	maize	SDA	TL 3	classification	[25]
water stress detection	abiotic stress	tomato	DT	TL 3	classification	[26]
salt stress detection	abiotic stress	wheat	SiVm	TL 3	classification	[27]
biochemical trait analysis	traits	maize, soybean	PLSR	TL 3	regression	[28]
detection of plant communication	traits	maize	LDA	TL 3	classification	[29]
disease forecast	biotic stress	barley	GAN	TL 3	classification	[30]
disease differentiation	biotic stress	cucumber	SDA	TL 4	classification	[31]
disease detection	biotic stress	sugarbeet	SVM	TL 4	classification	[32]

sists of the the line scanning unit, together with the illumination source. In a moving system, the light moves together with the camera unit [28]. If using a mirror, the illumination is fixed and changes along the scan area. The result is a hyperspectral datacube.

Choosing the right camera for a sensor setup has to take into account the spectral region of interest, signal-to-noise ratio, dynamic range, spectral and spatial resolution, pixel size, frame rate, lenses and operating temperature [39].

To acquire a proper datacube different calibration routines are needed to ensure highly accurate reflectance values. Figure 2 shows a generalized processing pipeline for hyperspectral cubes for the demands of plant imaging in greenhouses and laboratories as it is common for the demands of plant phenotyping.

Wavelength calibration - from pixel to wavelength

When using a pushbroom sensor one dimension of the detector represents the spatial information of the lines of the target. The other dimension represent the full spectrum of a single line of pixels. The wavelength calibration describes the comparison of measured spectral values with known values [40] and consequently, the mapping of the dispersed geometric access to wavelength in *nm*.

A calibration is needed after manufacturing and after any physical changes to the optical path [41]. Wavelength calibration is obtained by exposing the optical system to a calibration light source / sources. Three aspects are critical for obtaining a proper wavelength calibration including (i) the selection of the calibration light, (ii) the determination of the center of characteristic peaks and (iii) a polynomial fitting to the data [42]. The calibration light source / sources should cover the wavelength range to be calibrated. Wavelength calibration light sources emit atomic emission lines of known wavelengths. A polynomial fit of the geometric position of the atomic emission lines on the chip and the known wavelength is conducted. This step is usually performed preliminary by the manufacturer and enables displaying the spectral axis in units of wavelength (*nm*).

Instrument function / point spread function - overcoming spectral distortion

Measurements of any optical device can be described as a convolution of the original data with the appropriate transfer function of the sensor and optical setup. This convolution is characterized as a (spectral & spatial) blurring or smearing of the data [43]. The terms "instrument function" and "point spread function" are both used to describe this convolution. The term "point spread function" typically refers to the spatial convolution. The term "instrument function" is referring to the convolution in the spectral domain. Both terms define the highest possible spectral and spatial resolution. Effects resulting from the point spread function are described in the following paragraphs. In contrast to spatial distortions the (spectral) instrument function is typically not corrected for.

Spatial calibration - overcoming spatial distortion

Similar to 2D-RGB-cameras which come with barrel and pillow distortion [44], the images of a hyperspectral line scanner tend to show similar effects called smile and keystone effects. Smile is the curvature distortion of the horizontal spectra lines[33] or a shift in wavelength in the spectral domain [45]. Keystone is the distortion of the focal plane rectangle into a trapezoid [33] or a band-to-band misregistration [45]. These effects can be corrected using geometric control points (GCP) [33] . A spatial calibration of the hyperspectral cube describes the character of the spatial mapping process. This process results in an rectified image. Not all manufacturers provide this calibration by default.

Radiometric calibration - from counts to a physical unit

Due to differences in quantum efficiency of the detector and varying efficiency of the grating and other optical components (lenses etc.), measurements using different optical systems of the same object under same illumination conditions may not be identical [41]. To correct for such instrument related differences, radiometric calibration of the measurement device or white referencing is needed. Radiometric calibration uses an integrating sphere to measure the calibration coefficients for each wavelength band (pixel) that is measured by the sensor [46]. The camera output is mapped to a physical unit (luminous flux) using a certified spectral test specimen (integrating

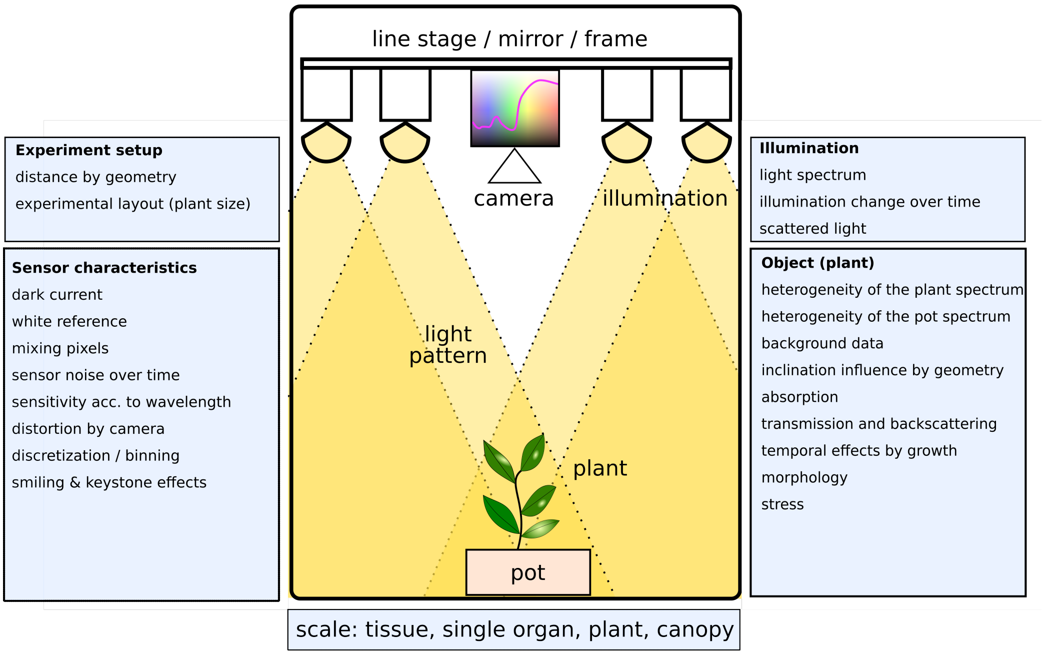

Figure 1. Influences on the measured spectrum of a plant. The four main sources of influence are the experimental setup, the way the camera is mounted, the distance to the plant etc., the light, its spectrum, focus, the object of interest with its absorbing and transmitting properties when imaging plants and the sensor in particular the dark and white referencing, its noise and sensitivity, distortion, discretization and binning.

sphere). Thus, radiometric calibration describes the spectral characterization of lens system, chip and dispersive elements (grating and filter).

In many applications absolute radiometric calibration is not required. Often it is sufficient to use a relative spectral calibration to correct for the spectrally varying system efficiency. A simple white referencing and dark subtraction is sufficient for reflectance measurement.

Spectral and spatial binning - reducing the noise level

To acquire a high retrieval accuracy within the acquired data a high signal to noise ratio (SNR) is required. SNR is the ratio of the radiance measured to the noise created by the detector and instrument electronics [4]. This ratio can be increased by combining spectral image information along the spectral axis (spectral binning) or by integrating the neighbour pixels (spatial binning) [39]. It was shown that binning along the spectral axis using just a few neighbours reduces of the (spectral) image size in favor of an enhanced signal to noise ratio [47].

In general, wavelength next to each other are highly correlated [48]. Thus it can be stated, that a slightly spectral binning will not affect the informative value of the remaining spectrum.

Binning can be performed directly at the camera internal hardware (hardware binning) or by a processing software when loading the datacube (software binning). In general, hardware binning results in less noise than software binning as the sensor signal is directly merged in the camera prior to analog digital conversion. If using hardware binning, this step has to be performed first before any calibration. If using software binning, it is the first step in the pre-processing right after the hardware calibration steps.

Data preprocessing

Pre-processing can be initiated after hardware calibration and measurement validation. A standardized process is needed to compare measurements from different timepoints and from different measuring setups. It includes pre-processing steps where the normalization is performed, the spectral smoothing and 3D correction up to a masking of the object of interest and data splitting, dimension reduction and feature selection for ML.

reflectance calibration / normalizing - overcoming the light source influence

To enable comparable measurements for time series within the same measurement setup, between different sensor setups or under different illumination conditions the normalization of the datacube according to the maximum and minimum reflectance intensity is needed. Therefore the dark image is captured by recording the hypercube with a lid on the camera or a closed shutter. This dark data cube described the lowest possible sensor signal. Right after this the white reference spectrum is acquired using a spectrally known reference target. Most often highly reflective materials like barium sulfate (SphereOptics.com) act as a reference. Alternatively the use of materials with a known spectral reflectance is established as a standard procedure. Performing the object scan right after including the associated dark image, the normalization step can be described by formula 1:

$$I_{Norm} = \frac{cube_O - cube_O^D}{cube_R^W - cube_R^D} \quad (1)$$

Within this formula $cube_O$ depicts the object cube respectively the $cube_O^D$ dark reference whereas $cube_R$ defines the reference object and $cube_R^W$ white reference and $cube_R^D$ dark reference [3] [4].

spectral smoothing - dealing with peaks and spectral outliers

Based on the assumption that the plant spectrum has a smooth spectrum and peaks within the spectrum are results of outliers and noise the use of soft smoothing algorithms is valid. Most established is the Savitzky-Golay smoothing algorithm [49] for hyperspectral data where 15 centered points and a polynomial of degree 3 has shown its applicability [50].

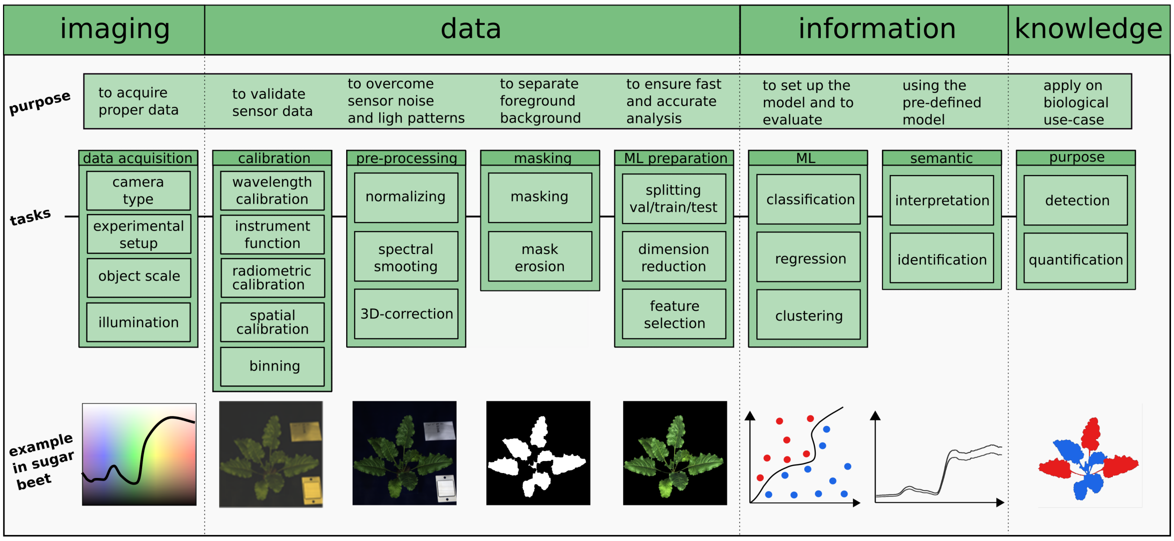

Figure 2. A generalization of a hyperspectral workflow The way to extract information from sensor data and to bring it into a biological context to generate knowledge starts with the data acquisition, the hardware calibration, a proper normalization step, data pre-processing, masking to focus on the object of interest, the plant and to cut out background, plant pot and stabilization sticks etc. Depending on the experiment setup data and the analysis type has to be divided into validation, training and test data set to train a model and to evaluate it on the test data. This is followed by the result interpretation and identification of diseases, stresses or other properties of the plants. Vertical dashed lines describe in a general way the transition between the imaging process, the processing of the data, the generation of information and by interpretation knowledge.

3D correction - correcting the influence of the sensor-object distance

The measured reflectance on the detector is depending on the reflected light intensity and the distance between sensor and reflection point on the object/plant. For measuring a plant with upper and lower leaves, the distance to the sensor is different for both leaves. This results in differences in the measured intensity. Some publications show the normalization of the spatial distance [18] [51]. A prerequisite for this is an integration of a 3D measuring device in the measuring setup (laserscanner, ultrasound etc.). Depending on the distance, the corrected cube contains equal reflectance values for similar surfaces although the distance to the camera is different by using pixelwise distance normalization.

segmentation masking

Image segmentation is used to partition an image into meaningful parts that have similar features and properties [52]. For the demands of plant phenotyping this usually means the separation from plant and background pixels. This is mostly based on simple vegetation indices or thresholds using a specific wavelength [53]. Further segmentation like the identification of single leaves, or the detection of disease symptoms are focused on later in the workflow pipeline as ML methods are used to tackle this problem.

After masking, the transition between fore- and background is very sharp. Pixels at this transition include parts of both classes and are depicted as "mixed pixels". To overcome the influence of these pixels to the analysis result, these pixels have to be removed. Literature shows that an the use of erosion as a binary image processing technique is efficient. A filter element the size of 3x3 pixels is used to shrink the region of the foreground [54]. A negative side effect related to the reduction of foreground data is the possibility of loosing important information which can be used to enhance the data quality.

preparation for ML

To prepare the data for use in a common ML routine, using supervised classification approaches, the dataset is split into three subgroups including the same distribution of groups within the three sets. That means the ratio between the included groups is similar. Set one is called validation dataset and is used for tests on dimension reduction, for feature extraction and for principal component analysis. Set two is called the training set and is used to calculate the model of the ML method like support vector machines (SVM) or decision trees (DT). The third set is called the test set and is used to validate the model and to calculate a model accuracy. The size of the groups differs with respect to the number available samples. A repeated cross-validation using different splits of the dataset (test and training) is recommended. To decrease redundancy within the dataset dimension reduction as it can be performed. State-of-the-art techniques are principal component analysis (PCA, [55]), feature selection using recursive feature elimination (RFE), ReliefF or correlation-based feature selection [56].

Data analysis and interpretation

hyperspectral traits

Hyperspectral traits can be grouped into different groups, depending of the focus of the data. If the data is coming from a single plant (trait level 1) the datacube can be used to derive very rough information about the plant like the plant canopy [57]. If the datacube is segmented into regions including single leaves, disease symptoms or spatially confined areas (ROI, trait level 2), these regions can be compared together. This is commonly done by a classification on pixel (single spectrum) level [58]. Time series measurements are essential for accurate capturing of developing disease symptoms. This leads to the development of spectral dynamics over time (trait level 3) [24] [50]. Hyperspectral datacubes are affected by distance and inclination of the measured object. The correction of thehyperspectral information according to distance and inclination is needed. This can be done by modeling the measuring setup and the occuring errors. It needs the use of an accompanying sensor measuring the object geometry as a 3D laserscanner [59] and fusing the data for a complete 3D-hyperspectral data model that enables detection of plant disease within a corrected spectrum [32]. An overview about these traits, prerequisites and applications is shown in Fig. 3.

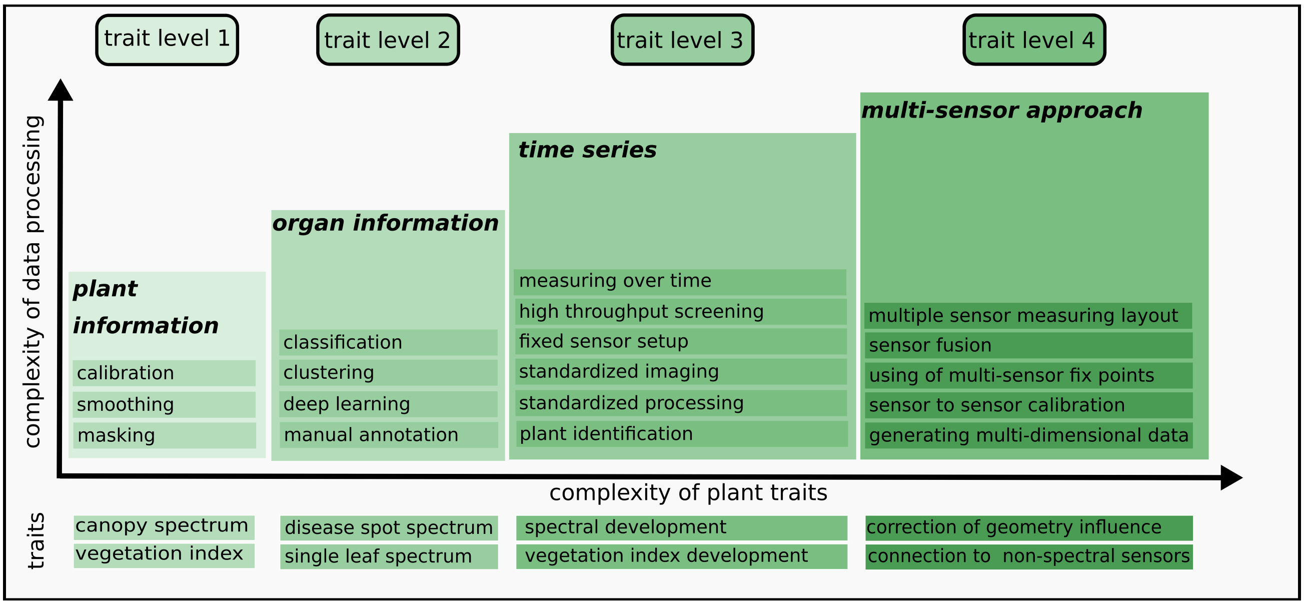

Figure 3. A general trait visualization Plant traits are parameters that describe the hyperspectral properties of the plant tissue. Nevertheless, these traits can be grouped according to the effort that is needed for their extraction. First level (1) traits describe the spectrum of the whole canopy. By using a classification based on ML algorithm it is possible to identify spectra of single leaves (level 2). By measurements over time the development of these spectra can be visualized (level 3) and by using further sensors it is possible to reduce geometrical effects based on a sensor fusion (level 4).

machine learning

For data analysis and ML, the tasks can be divided into supervised methods and unsupervised methods. Supervised methods require a known target value and therefore labeled data to train a model. Within the supervised learning methods, methods can be grouped by their target. If the output is a label as an affiliation to a group and the label is categorial, the method is called classification. Prominent routines for supervised classification are SVM, DT and Neural Network architectures (NN). A similar approach using labeled data is regression, where the output does not predict a group but a numeric value. Known methods for this scenario are Support Vector Regression (SVR), DT and NN.

A special case of ML is Deep Learning (DL). DL allows computational models that are composed of multiple processing layers to learn representations of data with multiple levels of abstraction. It also describes an algorithm allowing raw data as input and automatically discovers a representation, consisting of multiple non-linear modules, for detection or classification [60]. In contrast to SVM or DT approaches, DL is based on N architectures and is based on very huge datasets used for training. DL approaches have been widely used on RGB images for the demands of plant phenotyping as there is a classification of root tips, shoot and leaves [61] [62] and can be depicted to be state of the art. During the last years, hyperspectral applications are raising. Different types of DL approaches have been used for plant disease [63] or stress detection [64].

Unsupervised approaches do not need labeled data and try to detect patterns within the data. Clustering approaches like k-means shift manual work from model generation to cluster interpretation as it is the task of the scientist to give semantic to the clustered datasets. The clustering of hyperspectral datasets has been successfully shown for the detection of drought for maize [65].

Usually the results of a classification are presented by a confusion matrix, which indicates for a specific trained model the resulting classification of the test dataset regarding true positive, false positives, false negative and true negative. It compares the predicted values to the true values.

Challenges and limitations

HSI has to face many challenges regarding sensor setup, illumination, data processing and plant specific characteristics. Starting with the measuring setup where the sensor, illumination and the object distance has to be adapted to the plant size to gain best reflectance results. Thus, the setup has to tailored has to be tailored towards the size of the plantss. Both extrema within one measuring setup causes problems in illumination, image resolution and chip intensity.

When transferring hyperspectral imaging to the UV area between 200–400*nm*, plants can suffer from the harmful properties of illumination in this spectrum [5]. Further evaluation of the effects of light exposure on the study objects is recommended as plant properties such as architecture, tissue composure and wax layer differ between species.

Beside effects of the geometry, like the correlation between normalized difference vegetation index (NDVI) and inclination, have to be taken into account or if possible have to be corrected [7]. This emphasizes the need for imaging setups including different sensors for geometry and reflectance.

When transferring results from the laboratory or greenhouse to the field the workflow for using HSI is different and has to be designed individually [66]. Due to environmental conditions such as overcast and varying angles of sun exposure a specifically illumination normalization with a high frequency or even permanent has to be conducted. Furthermore, the use of masks rise from vegetation indices or wavelength thresholds loose quality due to shadows, focus plane if not measuring directly vertically. Measuring hyperspectral imaging on UAV brings the problem of image fusion especially when using line scanners [67], here the single line scans have to be merged together according to the 3D movement of the carrier.

Especially when using high throughput imaging setups [21] combined with hyperspectral cameras periodical imaging leads to huge datasets independent of the scale [37]. This emphasized the need for reliable, stable and efficient algorithms and high-end computational machines to process the datacubes. Image analysis and interpretation is the key plant phenotyping bottleneck [68].

Conclusion

HSI is a well-established tool for plant phenotyping in greenhouses. But each laboratory is using a specialized workflow for data assessing, processing and handling which makes the data individually valid but difficult to compare. This study introduces a generalized workflow of calibration, normalizing, smoothing and masking of spectral images up to the preparation for use in ML routines in greenhouses and laboratories. This workflow includes hardware-based calibration steps as well as software based processing. Furthermore, a general definition for hyperspectral traits is introduced to establish a level-system starting from traits for the whole plant, to traits for single organs, traits describing temporal development and traits that are based on the measurements of different sensors. An literature overview using hyperspectral imaging and ML is introduced to show the different application areas for plant measuring in agriculture together with the used ML method and the used plant material. Thus a general overview for the application of hyperspectral imaging in plant science is reasonable. This review offers a standardized protocol for raw data processing and how plant traits can be categorized due to their complexity regarding effort in data processing and derivable traits.

Declarations

List of abbreviations

- (A)NN - (artificial) neural network
- CNN - convolutional neural network
- DC - dark current
- DT - decision tree
- (F)LDA - (fishers) linear discriminant analysis
- FNN - fully connected neural network
- GAN - generative adversarial network
- HSI - hyperspectral imaging
- ML - machine learning
- NDVI - normalized difference vegetation index
- NIR - near infrared (700 – 1000*nm*)
- PLS (R) - partial least square (regression)
- RGB - red, green, blue, digital camera sensor
- SAE - stacked auto encoder
- SAM - spectral angle mapper
- SDA - stepwise discriminant analysis
- SNR - signal noise ratio
- SiVm - simplex volume maximization
- SVDD - support vector data descriptor
- SVM - support vector machines
- SWIR - short wave infrared (1000 – 2500*nm*)
- UV - ultra violett spectrum (< 380*nm*)
- VIS - visual spectrum (380 – 700*nm*)
- VNIR - visual + infrared spectrum (380 – 1000*nm*)
- WR - white reference

Ethical Approval (optional)

“Not applicable”

Consent for publication

“Not applicable”

Competing Interests

‘The authors declare that they have no competing interests’.

Funding

This study was partially funded by the Deutsche Forschungsgemeinschaft (DFG, German Research Foundation) under Germanys Excellence Strategy - EXC 2070 - 390732324. Furthermore it was supported by Bayer AG - Crop Science.

Author’s Contributions

SP and AKM designed the research. AKM supervised the project. SP and AKM designed the review manuscript, prepared the figures and studied the literature. All authors read and approved the final version of the article.

Acknowledgements

We would like to thank Oliver Lischtschenko from Ocean Optics B.V. for his helpful comments and suggestions regarding the hyperspectral sensor calibration. Furthermore we would like to thank Patrick Schramowski for proofreading the machine learning part and Abel Barreto for proofreading and his help with the figures.

Authors’ information (optional)

References

1. Mahlein AK, Kuska MT, Thomas S, Wahabzada M, Behmann J, Rascher U, et al. Quantitative and qualitative phenotyping of disease resistance of crops by hyperspectral sensors: seamless interlocking of phytopathology, sensors, and machine learning is needed! Current Opinion in Plant Biology 2019 Aug;50:156–162. https://doi.org/10.1016/j.pbi.2019.06.007.
2. Fiorani F, Rascher U, Jahnke S, Schurr U. Imaging plants dynamics in heterogenic environments. Current Opinion in Biotechnology 2012 Apr;23(2):227–235. https://doi.org/10.1016/j.copbio.2011.12.010.
3. Elmasry G, Kamruzzaman M, Sun DW, Allen P. Principles and Applications of Hyperspectral Imaging in Quality Evaluation of Agro-Food Products: A Review. Critical Reviews in Food Science and Nutrition 2012 Nov;52(11):999–1023. https://doi.org/10.1080/10408398.2010.543495.
4. Elmasry G, Sun DW. Principles of Hyperspectral Imaging Technology. In: Hyperspectral Imaging for Food Quality Analysis and Control Elsevier; 2010.p. 3–43. https://doi.org/10.1016/b978-0-12-374753-2.10001-2.
5. Brugger A, Behmann J, Paulus S, Luigs HG, Kuska MT, Schramowski P, et al. Extending hyperspectral imaging for plant phenotyping to the UV-range. Remote Sensing 2019 Jun;11(12):1401. https://doi.org/10.3390/rs11121401.
6. Behmann J, Acebron K, Emin D, Bennertz S, Matsubara S, Thomas S, et al. Specim IQ: Evaluation of a new, miniaturized handheld hyperspectral camera and its application for plant phenotyping and disease detection. Sensors 2018 Feb;18(2):441. https://doi.org/10.3390/s18020441.
7. Behmann J, Mahlein AK, Paulus S, Dupuis J, Kuhlmann H, Oerke EC, et al. Generation and application of hyperspectral 3D plant models: methods and challenges. Machine Vision and Applications 2015 Oct;27(5):611–624. https://doi.org/10.1007/s00138-015-0716-8.

8. Croft H, Chen JM. Leaf Pigment Content. In: Comprehensive Remote Sensing Elsevier; 2018.p. 117–142. https://doi.org/10.1016/b978-0-12-409548-9.10547-0.
9. Corti M, Gallina PM, Cavalli D, Cabassi G. Hyperspectral imaging of spinach canopy under combined water and nitrogen stress to estimate biomass, water, and nitrogen content. Biosystems Engineering 2017 Jun;158:38–50. https://doi.org/10.1016/j.biosystemseng.2017.03.006.
10. Behmann J, Steinrücken J, Plümer L. Detection of early plant stress responses in hyperspectral images. ISPRS Journal of Photogrammetry and Remote Sensing 2014 Jul;93:98–111. https://doi.org/10.1016/j.isprsjprs.2014.03.016.
11. Mahlein AK, Kuska MT, Behmann J, Polder G, Walter A. Hyperspectral Sensors and Imaging Technologies in Phytopathology: State of the Art. Annual Review of Phytopathology 2018 Aug;56(1):535–558. https://doi.org/10.1146/annurev-phyto-080417-050100.
12. Pierna JAF, Vermeulen P, Amand O, Tossens A, Dardenne P, Baeten V. NIR hyperspectral imaging spectroscopy and chemometrics for the detection of undesirable substances in food and feed. Chemometrics and Intelligent Laboratory Systems 2012 Aug;117:233–239. https://doi.org/10.1016/j.chemolab.2012.02.004.
13. Huang M, Wan X, Zhang M, Zhu Q. Detection of insect-damaged vegetable soybeans using hyperspectral transmittance image. Journal of Food Engineering 2013 May;116(1):45–49. https://doi.org/10.1016/j.jfoodeng.2012.11.014.
14. Yang W, Yang C, Hao Z, Xie C, Li M. Diagnosis of Plant Cold Damage Based on Hyperspectral Imaging and Convolutional Neural Network. IEEE Access 2019;7:118239–118248. https://doi.org/10.1109/access.2019.2936892.
15. Zhang SY, Fei T, Ran YH. Diagnosis of heavy metal cross contamination in leaf of rice based on hyperspectral image: a greenhouse experiment. In: 2018 IEEE International Conference on Advanced Manufacturing (ICAM) IEEE; 2018. p. 159–162. https://doi.org/10.1109/amcon.2018.8614938.
16. Nansen C, Zhao G, Dakin N, Zhao C, Turner SR. Using hyperspectral imaging to determine germination of native Australian plant seeds. Journal of Photochemistry and Photobiology B: Biology 2015 Apr;145:19–24. https://doi.org/10.1016/j.jphotobiol.2015.02.015.
17. Moghadam P, Ward D, Goan E, Jayawardena S, Sikka P, Hernandez E. Plant disease detection using hyperspectral imaging. In: 2017 International Conference on Digital Image Computing: Techniques and Applications (DICTA) IEEE; 2017. p. 1–8. https://doi.org/10.1109/dicta.2017.8227476.
18. Reddy KN, Huang Y, Lee MA, Nandula VK, Fletcher RS, Thomson SJ, et al. Glyphosate-resistant and glyphosate-susceptible palmer amaranth (*amaranthus palmeriS. Wats.*): hyperspectral reflectance properties of plants and potential for classification. Pest Management Science 2014 Mar;70(12):1910–1917. https://doi.org/10.1002/ps.3755.
19. Zhang C, Ye H, Liu F, He Y, Kong W, Sheng K. Determination and Visualization of pH Values in Anaerobic Digestion of Water Hyacinth and Rice Straw Mixtures Using Hyperspectral Imaging with Wavelet Transform Denoising and Variable Selection. Sensors 2016 Feb;16(2):244. https://doi.org/10.3390/s16020244.
20. Yu X, Lu H, Liu Q. Deep-learning-based regression model and hyperspectral imaging for rapid detection of nitrogen concentration in oilseed rape (Brassica napus L.) leaf. Chemometrics and Intelligent Laboratory Systems 2018 Jan;172:188–193. https://doi.org/10.1016/j.chemolab.2017.12.010.
21. Ge Y, Bai G, Stoerger V, Schnable JC. Temporal dynamics of maize plant growth, water use, and leaf water content using automated high throughput RGB and hyperspectral imaging. Computers and Electronics in Agriculture 2016 Sep;127:625–632. https://doi.org/10.1016/j.compag.2016.07.028.
22. Rumpf T, Mahlein AK, Steiner U, Oerke EC, Dehne HW, Plümer L. Early detection and classification of plant diseases with Support Vector Machines based on hyperspectral reflectance. Computers and Electronics in Agriculture 2010 Oct;74(1):91–99. https://doi.org/10.1016/j.compag.2010.06.009.
23. Leucker M, Mahlein AK, Steiner U, Oerke EC. Improvement of lesion phenotyping in *cercospora beticola*–sugar beet interaction by hyperspectral imaging. Phytopathology 2016 Feb;106(2):177–184. https://doi.org/10.1094/phyto-04-15-0100-r.
24. Behmann J, Bohnenkamp D, Paulus S, Mahlein AK. Spatial referencing of hyperspectral images for tracing of plant disease symptoms. Journal of Imaging 2018 Dec;4(12):143. https://doi.org/10.3390/jimaging4120143.
25. Nansen C, Sidumo AJ, Capareda S. Variogram analysis of hyperspectral data to characterize the impact of biotic and abiotic stress of maize plants and to estimate biofuel potential. Applied Spectroscopy 2010 Jun;64(6):627–636. https://doi.org/10.1366/000370210791414272.
26. Elvanidi A, Katsoulas N, Ferentinos KP, Bartzanas T, Kittas C. Hyperspectral machine vision as a tool for water stress severity assessment in soilless tomato crop. Biosystems Engineering 2018 Jan;165:25–35. https://doi.org/10.1016/j.biosystemseng.2017.11.002.
27. Moghimi A, Yang C, Miller ME, Kianian S, Marchetto P. Hyperspectral imaging to identify salt-tolerant wheat lines. In: Thomasson JA, McKee M, Moorhead RJ, editors. Autonomous Air and Ground Sensing Systems for Agricultural Optimization and Phenotyping II SPIE; 2017. p. 1182. https://doi.org/10.1117/12.2262388.
28. Pandey P, Ge Y, Stoerger V, Schnable JC. High throughput in vivo analysis of plant leaf chemical properties using hyperspectral imaging. Frontiers in Plant Science 2017 Aug;8. https://doi.org/10.3389/fpls.2017.01348.
29. do Prado Ribeiro L, Klock ALS, Filho JAW, Tramontin MA, Trapp MA, Mithöfer A, et al. Hyperspectral imaging to characterize plant–plant communication in response to insect herbivory. Plant Methods 2018 Jul;14(1). https://doi.org/10.1186/s13007-018-0322-7.
30. Forster A, Behley J, Behmann J, Roscher R. Hyperspectral Plant Disease Forecasting Using Generative Adversarial Networks. In: IGARSS 2019 - 2019 IEEE International Geoscience and Remote Sensing Symposium IEEE; 2019. p. 1793–1796. https://doi.org/10.1109/igarss.2019.8898749.
31. Berdugo CA, Zito R, Paulus S, Mahlein AK. Fusion of sensor data for the detection and differentiation of plant diseases in cucumber. Plant Pathology 2014 May;63(6):1344–1356. https://doi.org/10.1111/ppa.12219.
32. Roscher R, Behmann J, Mahlein AK, Dupuis J, Kuhlmann H, Plümer L. Detection of disease symptoms on hyperspectral 3D plant models. ISPRS Annals of Photogrammetry, Remote Sensing and Spatial Information Sciences 2016 Jun;III-7:89–96. https://doi.org/10.5194/isprs-annals-iii-7-89-2016.
33. Lawrence KC, Park B, Windham WR, Mao C. Calibration of a pushbroom hyperspectral imaging system for agricultural inspection. Transactions of the ASAE 2003;46(2). https://doi.org/10.13031/2013.12940.
34. Liang Z, Pandey P, Stoerger V, Xu Y, Qiu Y, Ge Y, et al. Conventional and hyperspectral time-series imaging of maize lines widely used in field trials. GigaScience 2017 11;7(2).

https://doi.org/10.1093/gigascience/gix117, gix117.
35. Seiffert U, Bollenbeck F, Mock HP, Matros A. Clustering of crop phenotypes by means of hyperspectral signatures using artificial neural networks. In: 2010 2nd Workshop on Hyperspectral Image and Signal Processing: Evolution in Remote Sensing IEEE; 2010. p. 1–4. https://doi.org/10.1109/whispers.2010.5594947.
36. Bruning B, Liu H, Brien C, Berger B, Lewis M, Garnett T. The Development of Hyperspectral Distribution Maps to Predict the Content and Distribution of Nitrogen and Water in Wheat (Triticum aestivum). Frontiers in Plant Science 2019 Oct;10. https://doi.org/10.3389/fpls.2019.01380.
37. Virlet N, Sabermanesh K, Sadeghi-Tehran P, Hawkesford MJ. Field Scanalyzer: An automated robotic field phenotyping platform for detailed crop monitoring. Functional Plant Biology 2017;44(1):143. https://doi.org/10.1071/fp16163.
38. Burnette M, Sagan V, Andrade-Sanchez P, Shakoor N, Sidike P, Ward R, et al. TERRA-REF Data Processing Infrastructure. In: Proceedings of the Practice and Experience on Advanced Research Computing - PEARC '18 ACM Press; 2018. p. 1–7. https://doi.org/10.1145/3219104.3219152.
39. Rehman SU, Kumar A, Banerjee A. SNR improvement for hyperspectral application using frame and pixel binning. In: Xiong XJ, Kuriakose SA, Kimura T, editors. Earth Observing Missions and Sensors: Development, Implementation, and Characterization IV SPIE; 2016. p. 134 – 139. https://doi.org/10.1117/12.2220599.
40. Gaigalas AK, Wang L, He HJ, DeRose P. Procedures for Wavelength Calibration and Spectral Response Correction of CCD Array Spectrometers. Journal of Research of the National Institute of Standards and Technology 2009 Jul;114(4):215. https://doi.org/10.6028/jres.114.015.
41. Yao H, Lewis D. Spectral Preprocessing and Calibration Techniques. In: Hyperspectral Imaging for Food Quality Analysis and Control Elsevier; 2010.p. 45–78. https://doi.org/10.1016/b978-0-12-374753-2.10002-4.
42. Sun YC, Huang C, Xia G, Jin SQ, Lu HB. Accurate wavelength calibration method for compact CCD spectrometer. Journal of the Optical Society of America A 2017 Mar;34(4):498. https://doi.org/10.1364/josaa.34.000498.
43. Kumar VN, Rao DN. Determination of the instrument function of a grating spectrometer by using white-light interferometry. Applied Optics 1997 Jul;36(19):4535. https://doi.org/10.1364/ao.36.004535.
44. Weng J, Cohen P, Herniou M. Camera calibration with distortion models and accuracy evaluation. IEEE Transactions on Pattern Analysis and Machine Intelligence 1992;14(10):965–980. https://doi.org/10.1109/34.159901.
45. Yokoya N, Miyamura N, Iwasaki A. Preprocessing of hyperspectral imagery with consideration of smile and keystone properties. In: Larar AM, Chung HS, Suzuki M, editors. Multispectral, Hyperspectral, and Ultraspectral Remote Sensing Technology, Techniques, and Applications III SPIE; 2010. p. 73 – 81. https://doi.org/10.1117/12.870437.
46. Hruska R, Mitchell J, Anderson M, Glenn NF. Radiometric and Geometric Analysis of Hyperspectral Imagery Acquired from an Unmanned Aerial Vehicle. Remote Sensing 2012 Sep;4(9):2736–2752. https://doi.org/10.3390/rs4092736.
47. Polder G, van der Heijden GW. Calibration and characterization of spectral imaging systems. In: Tong Q, Zhu Y, Zhu Z, editors. Multispectral and Hyperspectral Image Acquisition and Processing SPIE; 2001. p. 10 – 17. https://doi.org/10.1117/12.441362.
48. Mahlein AK, Rumpf T, Welke P, Dehne HW, Plümer L, Steiner U, et al. Development of spectral indices for detecting and identifying plant diseases. Remote Sensing of Environment 2013 Jan;128:21–30. https://doi.org/10.1016/j.rse.2012.09.019.
49. Savitzky A, Golay MJE. Smoothing and differentiation of data by simplified least squares procedures. Analytical Chemistry 1964 Jul;36(8):1627–1639. https://doi.org/10.1021/ac60214a047.
50. Bohnenkamp D, Kuska MT, Mahlein AK, Behmann J. Hyperspectral signal decomposition and symptom detection of wheat rust disease at the leaf scale using pure fungal spore spectra as reference. Plant Pathology 2019 Apr;68(6):1188–1195. https://doi.org/10.1111/ppa.13020.
51. Huang Y, Lee MA, Nandula VK, Reddy KN. Hyperspectral Imaging for Differentiating Glyphosate-Resistant and Glyphosate-Susceptible Italian Ryegrass. American Journal of Plant Sciences 2018;09(07):1467–1477. https://doi.org/10.4236/ajps.2018.97107.
52. Kaur D, Kaur Y. Various image segmentation techniques: a review. International Journal of Computer Science and Mobile Computing 2014;3(5):809–814.
53. Hillnhütter C, Mahlein AK, Sikora RA, Oerke EC. Use of imaging spectroscopy to discriminate symptoms caused by Heterodera schachtii and Rhizoctonia solani on sugar beet. Precision Agriculture 2011 Jun;13(1):17–32. https://doi.org/10.1007/s11119-011-9237-2.
54. Moghimi A, Yang C, Miller ME, Kianian SF, Marchetto PM. A Novel Approach to Assess Salt Stress Tolerance in Wheat Using Hyperspectral Imaging. Frontiers in Plant Science 2018 Aug;9. https://doi.org/10.3389/fpls.2018.01182.
55. Williams PJ, Kucheryavskiy S. Classification of maize kernels using NIR hyperspectral imaging. Food Chemistry 2016 Oct;209:131–138. https://doi.org/10.1016/j.foodchem.2016.04.044.
56. Moghimi A, Yang C, Marchetto PM. Ensemble feature selection for plant phenotyping: a journey from hyperspectral to multispectral imaging. IEEE Access 2018;6:56870–56884. https://doi.org/10.1109/access.2018.2872801.
57. Cao X, Luo Y, Zhou Y, Duan X, Cheng D. Detection of powdery mildew in two winter wheat cultivars using canopy hyperspectral reflectance. Crop Protection 2013 Mar;45:124–131. https://doi.org/10.1016/j.cropro.2012.12.002.
58. Bergsträsser S, Fanourakis D, Schmittgen S, Cendrero-Mateo M, Jansen M, Scharr H, et al. HyperART: non-invasive quantification of leaf traits using hyperspectral absorption-reflectance-transmittance imaging. Plant Methods 2015;11(1):1. https://doi.org/10.1186/s13007-015-0043-0.
59. Behmann J, Mahlein AK, Paulus S, Kuhlmann H, Oerke EC, Plümer L. Calibration of hyperspectral close-range pushbroom cameras for plant phenotyping. ISPRS Journal of Photogrammetry and Remote Sensing 2015 Aug;106:172–182. https://doi.org/10.1016/j.isprsjprs.2015.05.010.
60. LeCun Y, Bengio Y, Hinton G. Deep learning. Nature 2015 May;521(7553):436–444. https://doi.org/10.1038/nature14539.
61. Pound MP, Atkinson JA, Townsend AJ, Wilson MH, Griffiths M, Jackson AS, et al. Deep machine learning provides state-of-the-art performance in image-based plant phenotyping. GigaScience 2017 Aug;6(10). https://doi.org/10.1093/gigascience/gix083.
62. Mochida K, Koda S, Inoue K, Hirayama T, Tanaka S, Nishii R, et al. Computer vision-based phenotyping for improvement of plant productivity: a machine learning perspective. GigaScience 2018 Dec;8(1). https://doi.org/10.1093/gigascience/giy153.
63. Golhani K, Balasundram SK, Vadamalai G, Pradhan B. A review of neural networks in plant disease detection using hyperspectral data. Information Processing in Agri-

culture 2018 Sep;5(3):354–371. https://doi.org/10.1016/j.inpa.2018.05.002.

64. Singh AK, Ganapathysubramanian B, Sarkar S, Singh A. Deep Learning for Plant Stress Phenotyping: Trends and Future Perspectives. Trends in Plant Science 2018 Oct;23(10):883–898. https://doi.org/10.1016/j.tplants.2018.07.004.
65. Asaari MSM, Mertens S, Dhondt S, Wuyts N, Scheunders P. Detection of plant responses to drought using close-range hyperspectral imaging in a high-throughput phenotyping platform. In: 2018 9th Workshop on Hyperspectral Image and Signal Processing: Evolution in Remote Sensing (WHISPERS) IEEE; 2018. p. 121 – 138. https://doi.org/10.1109/whispers.2018.8747228.
66. Bohnenkamp D, Behmann J, Mahlein AK. In-Field Detection of Yellow Rust in Wheat on the Ground Canopy and UAV Scale. Remote Sensing 2019 Oct;11(21):2495. https://doi.org/10.3390/rs11212495.
67. Horstrand P, Guerra R, Rodriguez A, Diaz M, Lopez S, Lopez JF. A UAV Platform Based on a Hyperspectral Sensor for Image Capturing and On-Board Processing. IEEE Access 2019;7:66919–66938. https://doi.org/10.1109/access.2019.2913957.
68. Campbell ZC, Acosta-Gamboa LM, Nepal N, Lorence A. Engineering plants for tomorrow: how high-throughput phenotyping is contributing to the development of better crops. Phytochemistry Reviews 2018 Jul;17(6):1329–1343. https://doi.org/10.1007/s11101-018-9585-x.

 Click here to download Personal Cover Covering_Letter.docx

Dr. Stefan Paulus

Institute of Sugar Beet Research

Holtenser Landstr. 77

37079 Göttingen

Dear Sir or Madam,

Please find attached our manuscript “Technical workflows for hyperspectral plant image assessment and processing on the greenhouse and laboratory scale“ for submission in GigaScience.

It is a review publication focusing on the technical steps within a hyperspectral imaging workflow for measuring spectral plant traits for lab and greenhouse based research as it is essential for plant phenotyping. We see this publication as the basic step for a generalized and comparable collection of hyperspectral data between different scientists across the borders of plant science. Thus we think it fits very well to the aims and scopes of the GigaScience journal as it describes the technical basis for spectral omics data.

No part of the manuscript has been published before, nor is any part of it under consideration for publication at another journal. There are no conflicts of interest to disclose.

As reviewers we suggest to choose one specialist in spectrometry and one specialist in plant phenotyping.

Kind regards,

Stefan Paulus
