# Supplementary material for: Technical workflows for hyperspectral plant image assessment and processing on the greenhouse and laboratory scale
Source: Gigascience. 2020 Aug 20;9(8):giaa090. doi: 10.1093/gigascience/giaa090 (PMC7439585; doi:10.1093/gigascience/giaa090)
Supplement: giaa090_GIGA-D-20-00091_Revision_1 [file giaa090_giga-d-20-00091_revision_1.pdf]

GigaScience

Technical workflows for hyperspectral plant image assessment and processing on the greenhouse and laboratory scale

--Manuscript Draft--

**Manuscript Number:**	GIGA-D-20-00091R1
**Full Title:**	Technical workflows for hyperspectral plant image assessment and processing on the greenhouse and laboratory scale
**Article Type:**	Review
**Funding Information:**	Deutsche Forschungsgemeinschaft (EXC 2070 - 39073232) — Prof. Dr. Anne-Katrin Mahlein<br>Bayer CropScience — Prof. Dr. Anne-Katrin Mahlein
**Abstract:**	Using hyperspectral cameras is well established in the field of plant phenotyping, especially when using high throughput routines in greenhouses. Nevertheless, the used workflows differ depending on the applied camera, the imaged plants, the experience of the users and the measuring setup.<br>This review describes a general workflow for the assessment and the processing of hyperspectral plant data at the greenhouse scale. Aiming at a detailed description of possible error sources, a comprising literature review of possibilities to overcome these errors and influences is provided. The processing of hyperspectral data of plants starting from the hardware sensor calibration, the software processing steps to overcome sensor inaccuracies and the preparation for machine learning is shown and described in detail.<br>Furthermore, plant traits extracted from spectral hypercubes are categorized to standardize the terms used when describing spectral traits in plant phenotyping. Data is introduced from a scientific view on the data for canopy, single organs, plant development and also combined traits coming from spectral and 3D measuring devices.<br><br>This publications provides a structured overview on implementing hyperspectral imaging into biological studies.<br><br>Workflows have been categorized to define a trait level scale according to their metrological level and the processing complexity. A general workflow is shown to outline procedures and requirements to provide fully calibrated data of highest quality. This is essential for differentiation of tiny changes from spectral reflectance of plants, to track and trace spectral development as an answer to biotic or abiotic stresses.
**Corresponding Author:**	Stefan Paulus, Ph.D.<br>Institute of Sugar Beet Research<br>Göttingen, GERMANY
**Corresponding Author Secondary Information:**	
**Corresponding Author's Institution:**	Institute of Sugar Beet Research
**Corresponding Author's Secondary Institution:**	
**First Author:**	Stefan Paulus, Ph.D.
**First Author Secondary Information:**	
**Order of Authors:**	Stefan Paulus, Ph.D.<br>Anne-Katrin Mahlein, Professor
**Order of Authors Secondary Information:**	
**Response to Reviewers:**	All answers to the reviewers are well formatted added to the covering letter to the editor.

Powered by Editorial Manager® and ProduXion Manager® from Aries Systems Corporation

Reviewer reports:

Reviewer #1: The submitted manuscript reviewed relevant literature and summarized a general workflow for the analysis of plant hyperspectral images collected in controlled environments. This review could have a great impact to the research community: The general workflow could guide researchers to standardize the data acquisition and processing of plant hyperspectral images for controlled environment studies, help accumulate global research efforts, promote the data sharing, and ultimately advance big data analysis for plant spectral responses and therefore biological understanding. Therefore, the manuscript fits well with the journal's scope and could be of great interest to readers. There are some parts need to be further improved or explained.

1.In my opinion, a unique feature of spectral imaging is the combination of spatial and spectral information for objects rather than the combination of spatial and temporal information, which has been stated by the authors in the first paragraph in Background section.
•We appreciate this suggestion, text has been changed accordingly.
2.Details and explanations are needed for the data acquisition section. While line-scan (pushbroom) systems are widely used, many researchers also used area scanning mode (rarely point scanning, aka whiskbroom, mode) for studying plant spectral responses. To the best comprehensiveness, it would be better to briefly introduce all three scanning modes including basic system setup and pros and cons of using each mode. A figure may be added for the best illustration of the system setups.
•We appreciate this suggestion. A figure showing the different techniques for hyperspectral imaging has been added.
3.Data pre-processing (e.g., reflectance calibration or flat field correction)/meta-data information is utmost important for sharing plant hyperspectral images. Authors may consider to emphasize this importance and provide more information on how to select reference targets. For example, Spectralon targets are generally in good quality with known spectral characteristics, so data collected using this type of reference targets could be directly shared as long as the target model number and manufacturer are provided. In case Spectralon targets cannot be used (due to either cost consideration or spatial limitation), inexpensive alternative references can be used but the reference spectral characteristics should be provided as meta-data to ensure the reusability and comparableness of shared datasets.
•The link to the spectralon manufacturer was added, furthermore the sentence: “When sharing datasets the reference spectral characteristics should be provided as meta-data to ensure the reusability and comparableness.”
•Now this point should be emphasized.
4.Authors may consider use "flat field correction" as the name for the section of "reflectance calibration /normalizing …". An important feature of applying Eq.1. to images is to reduce nonuniformity caused by either the imaging chip, illumination, or both.
•This has been changed accordingly.
5.In the section of "preparation for ML", please consider adjusting the description order as "training", "validation", and "testing", which is logically natural and widely used by research communities. Authors may also consider cite a technical-driven review paper on feature selection. This will help readers to further the understanding and knowledge of the techniques can be potentially used.
•This has been changed accordingly.
6.It would be very interesting and useful if authors could provide a table to list some publicly available datasets that were collected by following the general workflow. This will in turn help the technical community to obtain domain datasets for the development of new tools in the future.
•We really appreciate this suggestion. Nevertheless, community is still lacking of hyperspectral datasets of plants with open/free access. This defines a todo for the future. We hope that this study will give a good basis for publishing a technical proper dataset.
7.There are some repeated words and typos to be carefully checked by the authors. For example: "publications" in the abstract and "bedefined" to "be defined" in the Data acquisition and processing section.
•This has been changed accordingly.

Powered by Editorial Manager® and ProduXion Manager® from Aries Systems Corporation

Reviewer #2: This is a review paper focusing on close-range hyperspectral imaging for plant assessment in the greenhouse and laboratory scales. Given the broad interest of using hyperspectral imaging for plant phenotyping research, as well as the complexity of data structure and analysis method, this manuscript is quite timely and relevant. The hyperspectral image is known for its large data volume. The topic thus is appropriate for the journal. The paper covered the topics including camera and measurement setup, data preprocessing, and data analysis/interpretation. The authors' argument is that a standardized workflow for image acquisition, processing and analysis is needed to make the data comparable among various labs, which is a valid point. The paper provides a good technical summary of hyperspectral imaging (such as camera and imaging stage setup, white referencing), and gives a good compilation of its applications on plant assessment that can be useful for the phenotyping research community. My major comments for the authors to consider improving the manuscript are in the following.

Section of spectral smoothing. The authors only discussed Savitzky-Golay method and missed many other methods that are common for spectral preprocessing.
In addition to spectral averaging (binning) that the authors also discussed, other methods like Multiplicative Signal Correction and Standard Normal Variate are also widely used. Other preprocessing such as first and second order derivative are also common. Note Savitzky-Golay can also be used for differentiation. I think you need to mention these methods rather than just Savitzky-Golay.
•We have added these methods together with a literature link.
Preparation for ML. You discussion of calibration set, validation set, and test set are not correct. In machine learning, calibration set is for model calibration (to calibrate model parameters), validation is for model hyper-parameter tuning, and the test set is to evaluate the performance of the developed model. Please make sure you express this correctly. In some implementations, an explicit validation set is not used where model calibration and hyper-parameter tuning are conducted together. In these implementations, test set is also referred to as validation set. I would recommend the authors to read some of the literature on NIRS analysis, as when the images are reduced to the spectrum level, the (pre)processing and analysis share commonalities. There are quite a few publications recently on using VIS-NIR-SWIR for leaf analysis in the context of plant phenotyping. Please study those so you can see calibration/validation schemes and spectral preprocessing.
•The description of the machine learning sets has been changed accordingly.
The explanation following Equation 1 was poor. I cannot understand it. Please revise.
•The explanation has been changed.
There is significant room for the authors to improve the writing and presentation of the manuscript. There are quite a few places where the wording and phrases can be improved. Please see my comments on the attached document.
•Comments in the PDF version of the draft have been inserted and the text changed accordingly.

Reviewer #3: This paper presents a workflow for researchers using hyperspectral imaging for phenotyping applications, specifically based in greenhouses and laboratory settings. This paper is very timely and quite necessary, in my opinion. Overall I think the paper is well organized and presents information that will be very useful for researchers as they design their experimental setups. My background is remote sensing, specifically hyperspectral, so many of my comments and suggestions are based on lining up the language in this manuscript with the language used in the existing remote sensing literature base. Since remote sensing researchers have been working with hyperspectral since the 1980s, I believe this will allow readers to find established and published methods that can directly apply to plant phenotyping without having to 'reinvent the wheel'. I am also assuming that most of your readers may not be familiar with hyperspectral. Especially since if I were new to hyperspectral for phenotyping, I would start with reading this paper!

General Comments:
*To be technically correct, use the term hyperspectral instead of spectral. RGB imagery is also spectral, but it just happens to be broadband and only three bands.
•This has been changed for the plant imaging sections. For the technical sections we

focused on the spectral calibration and the techniques, thus we think the term spectral is here appropriate.
*The camera characteristics and measuring setup section should be broken up into two sections. One for camera characteristics and one for the measuring setup. The camera characteristics description is thorough, but I would like to see more details (or more explicitly stated) on the experimental design or measuring setup. Specifically, the authors could elaborate on the following topics:
•As the authors mention, illumination is a significant factor in collecting high-quality data. Not all bulbs will work appropriately - what things do researchers need to know not to have illumination issues? Why should any fluorescent lights be turned off before collection?
•We clearly see that illumination is an highly important facor. Thus an extra section “Using illumination for measuring” has been added to the text.
•Side-view versus nadir image collection - why would you choose one over the other? Why will side view not translate to outdoor image collections?
•This has been added accordingly in the section “Measuring setup”.
•The inclusion of a reference panel (briefly mentioned in a different section) in the scene. Should it be all scenes or a preferred location within a scene?
•This has been added accordingly in the section “Measuring setup”.
•A discussion on the field of view of the camera and how to determine camera height based on the sample being collected and desired spatial resolution.
•This has been added accordingly in the section “Measuring setup”.
•Pushbroom versus integrating cameras
•This has been added accordingly in the section “Camera characteristics”.
•There needs to be a better description of each of the remote sensing data levels. At the moment, the terminology isn't quite correct, and the clarity is missing (Radiometric calibration section). Specifically, it would be important to define digital numbers, radiance, and reflectance data levels. They each have very different factors that influence them and require different corrections.
•This has been added accordingly in the section “Radiometric calibration”.
•Do not use the term normalization for reflectance retrieval. Reflectance calibration is ok, but to match the remote sensing literature, reflectance retrieval would be more accurate.
•This has been changed accordingly.

Specific Comments:
Abstract > Results: "This review describes a general workflow for the assessment and the processing of hyperspectral plant data at the greenhouse scale." I would add greenhouse and laboratory scale since this is the first mention of the measurement scale and it will match the title

•This has been changed accordingly.

Abstract > Conclusions: I would have this start on a new line like Background and Results.
•This has been changed accordingly.

"This publications provides a structured overview on implementing hyperspectral imaging into biological studies."
Publication should be singular. I would also add at the end "at the greenhouse and laboratory scale". This paper would not be useful for outdoor collections with UAV or airborne sensors.
•This has been changed accordingly.

Key Words: Make sure to include hyperspectral.
•This has been changed accordingly.

Key Points: hyperspectral not spectral, needs to be structure for evaluation of what?
•This has been changed accordingly.
"During the last years, spectral sensing of plants has developed as a valuable tool for plant phenotyping [1] [2]."
Rewording - "During recent years, hyperspectral sensing…." I think it is important to say hyperspectral instead of spectral. RGB is also spectral, but it just happens to be

broadband.
•This has been changed accordingly.

"The principle of hyperspectral imaging (HSI) is based on the fact that all materials reflect electromagnetic energy in prominent patterns and specific wavelength due to difference of their chemical composition and inner physical structure [3]. Spectroscopy is defined as the method of acquiring and explaining the spectral characteristics of an object regarding light intensity emerging from molecules at different wavelengths to provide a precise fingerprint of an object."

This sentence needs some rewording. This is not the principle of hyperspectral imaging but remote sensing in general. The difference between hyperspectral and other remote sensing is that hyperspectral is characterized by measuring hundreds of narrow bands in the electromagnetic spectrum. For any remote sensing sensor, the measured signature is the result of a material's chemical composition and inner/outer physical structure. It is important to note that the spectral signature is not just the inner leaf, especially since that depends on the part of the electromagnetic spectrum that is measured. Additionally, it is important to specify HOW hyperspectral is different than multi-spectral sensors (specifically RGB cameras are mentioned). In the paper, a lot of great examples are shown using hyperspectral. Still, I think the introduction could use one sentence saying why someone would invest the extra time/money/effort into using hyperspectral over an RGB camera. Lastly, spectroscopy can also be collected with a point spectrometer instead of an imager. There is a whole literature base that uses point spectroscopy for phenotyping, which is not the focus on this paper. I would add a single sentence acknowledging this difference. Also, it may not be apparent to readers that spectroscopy equals hyperspectral, and I would say that hyperspectral is more commonly used in the plant sciences literature.
•This has been changed accordingly. We hope that it now fulfills the claims of the reviewer.

"Spectral cameras have become affordable that increase the visible spectrum (400 - 700nm, VIS) of RGB-cameras by the ultra-violet (200 - 400nm, UV,[5]), the near infrared spectrum (700 - 1000nm,NIR, [6]) or even the short wave infrared spectrum (1000 - 2500nm, SWIR, [7])."
This sentence needs rewording. Hyperspectral cameras have become more affordable and as a result, more commonly used? Compared to RGB cameras, they increase the spectral resolution and spectral range?
•This has been changed accordingly.

Reflectance imaging of plants has been related to plant tissue characteristics [9], to detect abiotic stresses [10] or plant diseases [11].
This is the first time the term reflectance is used, and it might be easier for readers who are not familiar with this data type to use hyperspectral instead (until you get a chance to define reflectance in the Data Acquisition and Processing section). This list, as written, suggests these are the only applications of hyperspectral imaging of plants. I would add at the end "among others" to give some flexibility.
•This has been changed accordingly.

"To introduce HSI as a state-of-the-art tool for plant phenotyping a literature overview is presented showing the different biological objectives what hyperspectral sensors are used for in the laboratory and greenhouse scale starting from stress detection and disease classi¬fication to a linking to molecular analysis (QTL analysis) grouped by the introduced level-description."
Suggested rewording - "To introduce HSI as a state-of-the-art tool for plant phenotyping, a literature overview is presented showing the different biological objectives can be achieved with hyperspectral sensors in the laboratory and greenhouse settings including stress detection, disease classi¬fication, and molecular analysis (QTL analysis)."
•This has been changed accordingly and by suggestion of an other reviewer.

"The following paragraph introduced introduces techniques to overcome different ..."
Typos: "The following section introduces techniques ..."
•This has been changed accordingly.

Powered by Editorial Manager® and ProduXion Manager® from Aries Systems Corporation

	"A comprehensive literature review shows examples for hyperspectral application from biotic stress detection like disease or virus detection, abiotic stress detection like heavy metal or cold stress and plant trait extraction like biochemical traits or leaf water content."<br>Since this is the start of a paragraph, please include again this is at the greenhouse/laboratory scale.<br>•This has been changed accordingly.<br><br><br>Table 1: Please include in the caption this is for the greenhouse/laboratory scale. I'm not as familiar with hyperspectral greenhouse studies, but there is only one citation for each of these?<br>•A describing sentence has been added to the table caption.<br><br>"Spectral systems and resulting data differ in the way the camera is calibrated and the data is processed."<br>These are not the only ways hyperspectral systems differ. As mentioned in the following sections, there are many other factors. Perhaps a more generalized sentence? "Hyperspectral systems and resulting data will vary due to many factors, including camera characteristics, experimental setup, calibration, and data processing.<br><br>•This has been changed accordingly.<br><br>"… sensor wavelength calibration, the instrument function, the radiometric calibration and spectral and pixel binning."<br>What is "the instrument function"?<br>•Instrument function and point spread function need a detailed introduction which has been given in the section “Instrument function / point spread function - overcoming spectral distortion”<br><br><br>"Four categories of factors that influence the measured spectrum of plants can bedefi¬ned."<br>Add space between be and defined.<br>•This has been changed.<br>Also, these four factors are HUGE when collecting hyperspectral data and often result in the most errors or incorrectly interpreted data. I love the figure and that these factors are mentioned, but I think they could use a little more elaboration. How might each factor impact your data? The last sentence starts to address this, but in my opinion, it is too much of a summary of all of them. For example, spectra variability due to differences in genotypes is not caused by the optical configuration but the plant's properties.<br><br>•We clearly see this point and its importance. Nevertheless a quantification of the influence of the single error sources is rather complicated and needs test series with high quality calibrated recordings. Thus we hope that the summary approach is sufficient for publication.<br><br>Camera characteristics and measuring setup As mentioned in general comments, I believe this section should be split into two, which would allow authors to go into detail about how the measurement setup is critical for high-quality measurements. As I progress through specific comments, I will highlight sections that could be expanded on or moved to the measurement set up section.<br><br>•The section has been split and changed accordingly.<br><br><br>"Hyperspectral cameras for plant phenotyping often are line scanners (pushbrooms) as this type of sensor is commonly used in plant science or for high throughput analysis as it, unlike snapshot cameras, provides a very high spatial and spectral resolution."<br>This sentence is awkward and could use rewording. While they are often line scanners there are other hyperspectral camera systems. Since this is a literature review, mention those scanners and how they are different. In the measurement setup section, the pros/cons of each could be explained.

•This has been changed. Furthermore a complete new figure has been added.

"The next step, the transfer of these sensor types to the field scale has already been started for tracking the canopy development in cereals [37] or as an open-source and open data project of Terra-Ref [38]."
My remote sensing background has significant issues with this sentence. Hyperspectral data collection has been happening for decades with airborne sensors or point spectrometers for plant applications. Including predicting nitrogen content and canopy development. Since this sentence doesn't add to the camera characteristics section, I would remove it or reword so that it doesn't exclude a whole body of research (which is outside the scope of this paper).

•This has been changed. The sentence has completely been removed.

Wavelength calibration: I'm quite confused by this section. The wavelengths that sensor measures should be set by the manufacturer. Are there enough people creating their own hyperspectral sensors for this section to be applicable? In my experience, wavelengths rarely drift, and if they do, the manufacturer would prefer to do the correction. The sentence "The wavelength calibration describes the comparison of measured spectral values with known values [40] and consequently, the mapping of the dispersed geometric access to wavelength in nm." sounds like it is discussing reflectance retrieval, but that is a different section. The sentences "A polynomial ¬fit of the geometric position of the atomic emission lines on the chip and the known wavelength is conducted. This step is usually performed preliminary by the manufacturer and enables displaying the spectral axis in units of wavelength (nm)." Sounds like you are discussing the conversion of digital numbers to radiance, but that is also another section. I've also never heard the term dispersed geometric access, so it would probably be good to define? Now, it is important to know that each band has a spectral response function (again generally provided by sensor manufacturer or estimate by Gaussian function). This information is critical to resample a camera to another camera spectral resolution.

•We agree with this comment. A wavelength calibration should be performed by the manufacturer. The scope of this publication is to introduce all aspects of hyperspectral calibration. After a longer discussion with a specialist of a worldwide spectrometer manufacturer we can say that the majority of the users, especially scientists, build hyperspectral cameras, especially push broom and whisk broom systems themselves and thus need to perform their sensor wavelength calibration by themselves.
•The sentence has been changed access axis. The confusion should be solved now.
•We deeply re-discussed this text section and think that we could improve the quality and readability. This passage only focusses on mapping of pixel position on the camera to wavelength and not about reflectance or radiance measuring.

"Due to differences in quantum efficiency of the detector and varying efficiency of the grating and other optical components (lenses etc.), measurements using different optical systems of the same object under same illumination conditions may not be identical [41]."
A sentence needs to follow this one that spells out to the reader that this data level is called digital numbers. This data-level is influenced by sensor characteristics, atmospheric conditions, and surface properties (in this example plants). This will emphasize the reason why sensors at this data type level are not comparable.

This has been changed accordingly.

"To correct for such instrument related differences, radiometric calibration of the measurement device or white referencing is needed."
*White reference is NOT used for radiometric calibration. Many software programs will incorporate the radiance to reflectance step into one which would use the white reference. However, the term white referencing is specifically for converting to reflectance. This is a critical difference when making measurements outdoors, but it worth separating here.
This has been changed accordingly.

*It is also important to tell the reader what the radiance is, especially since there are many plant applications (such as photosynthetic studies) that require radiance values, not reflectance. This data product is influenced by the light source, atmospheric absorptions, and surface properties, but it does remove camera factors.
Now it should be more clear and more explicit.
*To convert from DNs to radiance, a gain and offset per band are applied to the data which are provided by the sensor designers or engineers. Software provided by the manufacturer should have those values automatically provided. IF they don't, then you have to develop them yourself, which is the description actually provided in this section.
"In many applications absolute radiometric calibration is not required. Often it is sufficient to use a relative spectral calibration to correct for the spectrally varying system efficiency. A simple white referencing and dark subtraction is sufficient for reflectance measurement."
This needs to be reworded. Right now, it jumps from radiometric calibration to a reflectance measurement - which has not been defined. Also, this depends on the camera system. Often it is possible to 'skip' the radiance conversion because it a linear regression with DNs, but this is not the case with every camera (depending on the camera characteristics it can be non-linear spectrally and spatially).
This has been changed accordingly. And a few more explicit sentences have been added.

Spectral and spatial binning: Yes, SNR can be increased when data is binned, but many new users will do this incorrectly. For example, many hyperspectral sensors have 'bad bands' towards the upper and lower range of the sensor. Bad bands are those defined with having very high noise and unreliable measurements. These bad bands are lower SNR ratio than other bands because they are at the upper limits of the sensor's capabilities. There can also be bad bands due to atmospheric conditions, which in a greenhouse with high water vapor could be strong. I also feel like this is not necessary for all cameras and really depends on the SNR of the camera used. My suggestion would be to word this as an optional step and explain when a user should consider these methods. Especially since there are sections on dimensionality reduction and spectral smoothing which also impacts the spectral data.

This has been changed. A new sentence dealing with the lower and upper spectral area and how to handle it has been added.

"Thus it can be stated, that a slightly spectral binning will not affect the informative value of the remaining spectrum."
Slightly? I think a different word might more appropriate.

•This has been changed to “limited” we hope that this is fine now.

"It includes pre-processing steps where the normalization is performed, the spectral smoothing and 3D correction up to a masking of the object of interest and data splitting, dimension reduction and feature selection for ML."
This sentence is awkward and could use rewording.
•The sentence has been changed.

Reflectance Calibration: Do not use the term normalization for reflectance retrieval. Define what the reflectance data level is and what the units are. It is important for the readers to know that this data level removes camera effects, atmospheric conditions, and lighting effects, so only the surface properties remain. THIS data source is comparable across camera systems, whereas the other data levels are not.
•The term normalization has been changed in the complete study article. Furthermore a more appropriate definition has been added.

"Most often highly reflective materials like barium sulfate (SphereOptics.com) act as a reference."
In my opinion, the reference panel is one of the most critical components of making high-quality hyperspectral measurements. I would love to see this in an experimental setup section with a lot more details. For example, the material does need to be highly reflective but also highly reflective across the entire spectral range of the camera

Powered by Editorial Manager® and ProduXion Manager® from Aries Systems Corporation

	measures. Also, probably the most commonly used panel (but of course more expensive) is a spectralon panel made by Labsphere. White paint for camera measuring 400-1000 nm can also be sufficient.<br>•Spectralon has been added as well as “across the entire spectral range”<br><br>"Alternatively the use of materials with a known spectral reflectance is established as a standard procedure."<br>Yes! I always recommend a black, light gray, and dark gray target also. These can be measured with a point spectrometer to get a known reflectance value.<br>•This sentence has been added to the text.<br><br>"Performing the object scan right after including the associated dark image, the normalization step can be described by formula 1:"<br>This formula is the most basic way of converting from radiance to reflectance, using only a single target. In the remote sensing literature, it is referred to as the empirical line correction method. However, if you have a variable atmosphere (such as a greenhouse with fluctuating values) or are covering a large area, a single target may not be sufficient for good data. This is also true if the lighting conditions change or if the data set is a time series. A more advanced empirical line correction method incorporates multiple targets, which can make it robust to these changes. Conversion to reflectance from radiance generally results in the largest data errors, so in my opinion is worth elaborating.<br>•To emphasize this point a new sentence was added.<br>•“For measurements in a greenhouse with a variable environment like a change in light condition, or when measuring time series or measurements that cover a large area it it recommended to use multiple targets or periodical re-calibration of the sensor setup.”<br><br>"Based on the assumption that the plant spectrum has a smooth spectrum and peaks within the spectrum are results of outliers and noise the use of soft smoothing algorithms is valid."<br>This sentence needs to be clarified. Plant spectra can have peaks or valleys that are due to biophysical or structural conditions that people may be interested in. Very sharp peaks that only span one or two wavelengths are definitely noise. This is where a discussion of 'bad bands' that I mentioned before would be useful. Again, this may not apply to all cameras and it may not apply to the whole spectrum depending on the SNR.<br>•We see this point and appreciate this indication. Thus the part “covering just one or two bands” was added to clarify this.<br><br>"Most established is the Savitzky-Golay smoothing algorithm [49] for hyperspectral data where 15 centered points and a polynomial of degree 3 has shown its applicability [50]."<br>This is highly dependent on the camera's spectral resolution.<br>•That is right. The sentence has been changed and the camera of the example has been added.<br><br>"Literature shows that an the use of erosion as a binary image processing technique is efficient."<br>Typo shown in italics. There should be a citation with this statement or is it the same as the following sentence? Might be worth mentioning that some machine learning algorithms are robust to them anyway. Also, I'm sure you are aware there is a whole literature for working with mixed pixels which might be helpful for readers to know if their spatial resolutions are coarse.<br>•The authors think that Moghimi 2018 should be enough as a citation. The fact that some but not all methods are robust to mixed pixels is right, but we think that this will be beyond the scope of this study as we do not want to focus on ML techniques. But we are thankful for this comment.<br><br>Preparation for ML: I love that the authors chose to focus on machine learning techniques. I have found too many phenotyping papers that rely on a vegetation index to retrieve their trait of interest. Why do we have cameras that measure hundreds of bands if we are going to reduce them down to one value? I would love to see one sentence on why researchers should use ML approaches rather than a vegetation index.

	•This has been changed. A short paragraph has been added in the beginning of “preparation for ML”<br><br>"To decrease redundancy within the dataset dimension reduction as it can be performed."<br>I would like to suggest a clarification for this sentence - "Dimensionality reduction methods can decrease spectral redundancy and reduce data volume within the dataset."<br>•This has been changed accordingly.<br><br>"State-of-the-art techniques are principal component analysis (PCA, [55]), feature selection using recursive feature elimination (RFE), ReliefF or correlation-based feature selection [56]."<br>I would change "state-of-the-art" to common since PCA was one of the very first dimensionality reduction techniques. Or split it into two sentences - one with common and another with new algorithms.<br>•This has been changed.<br><br>"If the data is coming from a single plant (trait level 1) the datacube can be used to derive very rough information about the plant like the plant canopy [57]."<br>Very rough information? What does this mean? Instead of like, I would suggest "such as"<br>•This has been changed. Rought -> low resolved<br><br>"The correction of thehyperspectral information according to distance and inclination is needed."<br>Space needed between the and hyperspectral.<br>•This has been changed.<br><br>"In contrast to SVM or DT approaches, DL is based on N architectures and is based on very huge datasets used for training."<br>Consider rewording to remove duplicate "is based on"<br>•This has been changed.<br><br>"DL approaches have been widely used on RGB images for the demands of plant phenotyping as there is a classi¬fication of root tips, shoot and leaves [61] [62] and can be depicted to be state of the art."<br>Remove there is.<br>•This has been changed.<br><br><br>"Usually the results of a classi¬fication are presented by a confusion matrix, which indicates…"<br>Since the previous sentence said no labeled data was needed, it might be worth mentioning that the confusion matrix does need labeled data.<br>•This is right, nevertheless, the labeled data is needed for evaluation. To clarify this, this paragraph was moved to the supervised section.<br><br>"Thus, the setup has to tailored has to be tailored towards the size of the plantss."<br>Remove duplicate s on plants.<br>•This has been changed.<br><br>"Beside effects of the geometry, like the correlation between normalized difference vegetation index (NDVI) and inclination, have to be taken into account or if possible have to be corrected [7]."<br>NDVI is not only influenced by leaf inclination but also more broadly canopy structure.<br>•The text clearly says that the datacube is affected by distance and inclination which includes effects of the canopy structure. The authors think an additional emphasizing of this aspect is not necessary. Thus, the text was not changed .<br><br>"When transferring results from the laboratory or greenhouse to the ¬field the work ow for using HSI is different and has to be designed individually [66]."<br>I think this paragraph should be condensed significantly since it is definitely out of the scope of the paper and a single paragraph would not be sufficient to explain how this

Powered by Editorial Manager® and ProduXion Manager® from Aries Systems Corporation

	workflow would be transferrable to field settings. The reflectance retrieval process (referred to as normalization here) is completely different for field collections. As mentioned most everything is different. I would summarize by saying "The workflow proposed is not transferrable to field conditions which requires a very different experimental set up to ensure high quality hyperspectral measurements."<br>•This has been changed.<br><br>"Especially when using high throughput imaging setups [21] combined with hyperspectral cameras periodical imaging leads to huge datasets independent of the scale [37]."<br>This sentence is should be reworded.<br>•This has been changed.
Additional Information:	
Question	Response
Are you submitting this manuscript to a special series or article collection?	No
**Experimental design and statistics**<br><br>Full details of the experimental design and statistical methods used should be given in the Methods section, as detailed in our Minimum Standards Reporting Checklist. Information essential to interpreting the data presented should be made available in the figure legends.<br><br>Have you included all the information requested in your manuscript?	Yes
**Resources**<br><br>A description of all resources used, including antibodies, cell lines, animals and software tools, with enough information to allow them to be uniquely identified, should be included in the Methods section. Authors are strongly encouraged to cite Research Resource Identifiers (RRIDs) for antibodies, model organisms and tools, where possible.<br><br>Have you included the information requested as detailed in our Minimum Standards Reporting Checklist?	Yes
**Availability of data and materials**	Yes

Powered by Editorial Manager® and ProduXion Manager® from Aries Systems Corporation

All datasets and code on which the conclusions of the paper rely must be either included in your submission or deposited in publicly available repositories (where available and ethically appropriate), referencing such data using a unique identifier in the references and in the “Availability of Data and Materials” section of your manuscript.<br><br>Have you have met the above requirement as detailed in our Minimum Standards Reporting Checklist?	

Powered by Editorial Manager® and ProduXion Manager® from Aries Systems Corporation

Main Document Click here to access/download;LaTeX - Main Document (TeX file);main.tex

Warning. The length \marginparwidth is less than 2cm and will most likely cause issues with the appearance of inserted todonotes. The issue can be solved by adding a line like \setlength{\marginparwidth}{2cm} prior to loading the todonotes package.

GigaScience, 2017, 1–12

doi: xx.xxxx/xxxx
Manuscript in Preparation
Review

REVIEW

Technical workflows for hyperspectral plant image assessment and processing on the greenhouse and laboratory scale

Stefan Paulus[1,*] and Anne-Katrin Mahlein[1,†]

[1]Institute of Sugar Beet Research, Holtenser Landstr. 77, 37079 Göttingen, Germany

[*]paulus@ifz-goettingen.de;
[†]mahlein@ifz-goettingen.de

Abstract

Background: Using hyperspectral cameras is well established in the field of plant phenotyping, especially when using high throughput routines in greenhouses. Nevertheless, the used workflows differ depending on the applied camera, the imaged plants, the experience of the users and the measuring setup.
Results: This review describes a general workflow for the assessment and the processing of hyperspectral plant data at the greenhouse and laboratory scale. Aiming at a detailed description of possible error sources, a comprising literature review of possibilities to overcome these errors and influences is provided. The processing of hyperspectral data of plants starting from the hardware sensor calibration, the software processing steps to overcome sensor inaccuracies and the preparation for machine learning is shown and described in detail.
Furthermore, plant traits extracted from spectral hypercubes are categorized to standardize the terms used when describing hyperspectral traits in plant phenotyping. Data is introduced from a scientific view on the data for canopy, single organs, plant development and also combined traits coming from spectral and 3D measuring devices.
Conclusions This publication provides a structured overview on implementing hyperspectral imaging into biological studies at the greenhouse and laboratory scale. Workflows have been categorized to define a trait level scale according to their metrological level and the processing complexity. A general workflow is shown to outline procedures and requirements to provide fully calibrated data of highest quality. This is essential for differentiation of tiny changes from hyperspectral reflectance of plants, to track and trace hyperspectral development as an answer to biotic or abiotic stresses.

Key words: plant phenotyping, camera calibration, machine learning, hyperspectral signature, hyperspectral

Background

During recent years, hyperspectral sensing of plants has developed as a valuable tool for plant phenotyping [1] [2]. The principle of hyperspectral imaging (HSI) is based on the fact that all materials reflect electromagnetic energy in prominent patterns and specific wavelength due to difference of their chemical composition, inner physical structure and surface properties. This signal is characterized by measuring hundreds of narrow bands within the electromagnetic spectrum [3]. Spectroscopy is defined as the method of acquiring and explaining the hyperspectral characteristics of an object regarding light intensity emitted, reflected or transmitted from molecules at different wavelengths to provide a precise fingerprint of an object. Hyperspectral imaging combines spectral and spatial information similar to a digital camera [4]. Hyperspectral Imaging extends the measurable spectral range from the visible (RGB camera) to the NIR range and sample the spectrum in many narrow bands (> 20 bands). If only a few (< 20) spectral bands were samples literature depicts this as multispectral. Compared to spectroscopy, which measures the same spectral area HSI is able to measure spectral and spatial information in

Compiled on: June 25, 2020.
Draft manuscript prepared by the author.

Key Points

- a literature overview is provided to describe aims and scopes of hyperspectral sensing of plants and the different types of analysis methods
- hyperspectral workflows for plant measuring are highly individual and need to be structured for result comparison and evaluation
- a general workflow for hyperspectral plant phenotyping including camera calibration, segmentation and machine learning analysis is shown
- a level-based trait definition is introduced for canopy, plant organ, time series and sensor fusion

an images which enables a more detailed analysis of the object.

Spectral cameras have become affordable during the last years. Unlike RGB cameras imaging the visible spectrum (400 – 700*nm*, VIS) this area is extended by the ultra-violet (200 – 400*nm*, UV,[5]), the near infrared (700 – 1000*nm*, NIR, [6]) or even the short wave infrared spectrum (1000 – 2500*nm*, SWIR, [7]). This is highly interesting for plant science as many plant traits and biophysiological processes can be traced beyond the visible spectral range [8]. Hyperspectral imaging of plants has been related to plant tissue characteristics [9], to detect abiotic stresses [10] or plant diseases [11] among others.

Typically, laboratory workflows differ in their use of cameras, measuring setups and data handling such as calibration, smoothing and segmentation. There are several hardware calibration steps to understand and execute, starting from the camera pixel position mapping to the proper wavelength, the correction of the camera and lens distortion to the correction of the 3D setup when measuring upper and lower leaves of a plant. Thus, a standardized introduction of a workflow of hyperspectral image processing is needed to enable the comparison of results from different laboratories regarding their hyperspectral analysis.

To introduce HSI as a state-of-the-art tool for plant phenotyping, a literature overview is presented showing the different biological objectives what hyperspectral sensors are used for in the laboratory and greenhouse scale. The overview comprises stress detection, disease classification and linking to molecular analysis (QTL analysis). All found use-cases were grouped by the introduced level-description.

The following section introduces techniques to overcome different impairments on the measured spectrum coming from the experimental setup, the sensor, the role of illumination and the challenges when measuring complex plants with plant specific optical properties. The complete workflow from sensor adjustment, correction, calibration, segmentation to the extraction of hyperspectral plant traits and to a deeper analysis using routines of machine learning (ML) to extract biological information is described.

The application part describes the different aspects of plant traits based on HSI. Finally, a level-description model is introduced from the perspective of a data scientist. It describes the increase of complexity in data acquisition and data handling, when switching from an averaged spectrum of the plant canopy to an organ-specific spectrum to spectral development in time course to multi-sensor plant models. The latter is needed for the geometrical correction of the spectral data.

HSI a tool for plant screening

A comprehensive literature review shows examples for hyperspectral application from biotic stress detection like disease or virus detection, abiotic stress detection like heavy metal or cold stress and plant trait extraction like biochemical traits or leaf water content at the greenhouse and laboratory scale. Table 1 emphasizes different use-cases from plant science, where hyperspectral imaging cameras were used to differentiate between different situations.

In Table 1 hyperspectral data was grouped by trait level which describes the complexity of the traits. Starting from simple image analysis (level 1), to organ identification (level 2), to time series (level 3) and to a final multi-sensor data acquisition (level 4). It is shown that HSI is used for classification and regression problems across all trait levels (1-4). A closer introduction into these phenotypic trait levels can be found below in the text.

Three main groups can be identified including i) detection and quantification of biotic stress like disease detection [11], ii) detection and quantification of abiotic stress like heavy metal [15] or water stress [26] and iii) extraction of plant traits to describe water content [21] or biochemical traits [28].

Thus, HSI is widely used for different aspects of plant screening and can be depicted to be a state-of-the-art tool for plant phenotyping.

Data acquisition and processing

Hyperspectral systems and resulting data will vary due to many factors, including camera characteristics, experimental setup, calibration, environmental characteristics and data processing. This leads to inconsistencies regarding the data quality and the validity of results. This increases the difficulty to compare data from different sensors. Multiple steps are needed to acquire valid physical reflectance data starting from the sensor wavelength calibration, the instrument function, the radiometric calibration and spectral and pixel binning.

The goal of calibration is to standardize the spectral axis, determining if the sensor is working properly, providing the accuracy of the extracted data, validate the credibility and quantify the instrument errors, accuracy and reproducibility under different operating conditions [4].

Four categories of factors that influence the measured spectrum of plants can be defined (see Figure 1). I) the experimental setup including the optical configuration II) the sensor characteristics including sensor offset, noise and sensitivity behaviour and distortion effects [34] III) the illumination effects from the light source when using active illumination or the surrounding light when using environmental light and IV) object and its properties. Plant object properties means spectral variability due to differences in genotypes, plant organs, materials within the image such as pot and background data, inclination influence due to the architecture of plants, absorption, transmission and backscattering as plant tissue properties and temporal effects due to growth.

Camera characteristics

HSI can be performed using three different sensor types the

Table 1. Hyperspectral imaging is widely used for detection of biotic and abiotic stresses as well as for trait description. Traits are categorized by a complexity description starting from trait level 1 (TL1, whole plant), trait level 2 (TL2, organ specific traits), trait level 3 (TL 3, time series) and trait level 4 (TL4, multi sensor traits). The following overview describes a representative selection for the greenhouse and laboratory scale

purpose	group	plant	method	trait level	target	reference
detection of impurities in seeds	traits	wheat, spelt, barley	SVM	TL 1	classification	[12]
insect damage detection	biotic stress	soybean	SVDD	TL 1	classification	[13]
cold stress detection	abiotic stress	maize	CNN	TL 1	regression	[14]
heavy metal stress detection	abiotic stress	rice	SVM	TL 1	classification	[15]
germination detection	traits	trees	LDA	TL 1	classification	[16]
virus detection	biotic stress	tomato, tobacco	SVM	TL 1	classification	[17]
weed resistance analysis	traits	amaranth	FLDA	TL 1	classification	[18]
ph-value determination	traits	rice & water hyacinth	PLS & NN	TL 1	regression	[19]
nitrogen concentration	traits	oilseed rape	SAE & FNN	TL 1	regression	[20]
leaf water content	traits	maize	PLSR	TL 1	regression	[21]
disease detection	biotic stress	sugar beet	ANN, DT, SVM	TL 2	classification	[22]
disease resistance & QTL analysis	biotic stress	sugar beet	SAM	TL 2&3	classification	[23]
disease development	biotic stress	wheat	DT	TL 2&3	classification	[24]
biomass & biofuel potential	traits	maize	SDA	TL 3	classification	[25]
water stress detection	abiotic stress	tomato	DT	TL 3	classification	[26]
salt stress detection	abiotic stress	wheat	SiVm	TL 3	classification	[27]
biochemical trait analysis	traits	maize, soybean	PLSR	TL 3	regression	[28]
detection of plant communication	traits	maize	LDA	TL 3	classification	[29]
disease forecast	biotic stress	barley	GAN	TL 3	classification	[30]
disease early detection	biotic stress	sugar beet	SVM, PLS, DT	TL 3	classification	[31]
disease differentiation	biotic stress	cucumber	SDA	TL 4	classification	[32]
disease detection	biotic stress	sugarbeet	SVM	TL 4	classification	[33]

push broom / line scanner, the filter-based sensor setup and a whisk broom setup (see Figure 2). Push broom cameras scan the region below the sensor in lines and complete the full scan by either moving the sensor [28] or by using a mirror that is panned over the object of interest. A filter based system is measuring the complete region of interest using different filters either by splitting the scan ray using prisma or by using a combined filter pattern. Whisk broom sensors measure the full spectral range pixel by pixel similar to a spectrometer that is moved over the region of interest. All three setups result in a three-dimensional hypercube showing two spatial axis and one spectral axis.

Whisk broom sensors have more moving parts and thus are likely to wear out. Push broom cameras have less moving parts but need a high quality calibration as the different regions of the chip can show different sensitivity which can result in stripes within the datacube. Filter based systems are commonly restricted by the number of filters and provide less spectral resolution. Currently state-of-the-art plant phenotyping centers use mostly push broom line scanners.

Measuring setup

Choosing the right camera for a sensor setup has to take into account the point of interest side-view or from top-view setup, depending on if one single image from top is sufficient or if multiple images by rotating the plant are needed. Furthermore the spectral region of interest which is different depending on the camera chip (silicon for 380 – 1000*nm*, indium-gallium-arsenide for 1000 – 2500*nm*), the focal length, the minimum working distance, the maximum resolution resulting from sensor height and plant height, the focused signal-to-noise ratio, dynamic range, spectral and spatial resolution, pixel size, frame rate, lenses and operating temperature [35]. In general the field of view should cover the complete plant from small seedlings to the bigger plants in a timeseries experiment. This is accompanied by a periodical adaption of the focal plane as the plant height is changing due to plant development. Here the desired resolution has to be considered as the ratio between plant pixels and background pixels is changing permanently. For reference panels the options are a permanent reference measuring after each plant if using a box design, referencing within the measurable volume at the same height as the majority of the plant pixels or a periodical referencing along the scan axis when using a measuring setup at a longer line stage.

Illumination for measuring

Illumination is essential for HSI, but not every light source can be used. The use of passive light like sunlight which is available outdoors and in greenhouses is valid. Active light sources need a closer consideration. Tungsten halogen lamps are broad band emitter (400 – 2600*nm*) can be used, are economically affordable and technically easy to setup. Whereas gas discharge tubes (fluorescent tubes or uncoated tubes) are not usable as these tubes emit high narrow lines in the spectrum. Nevertheles, deuterium gas discharge can be used for UV measuring applications and arc sources like xenon lamps can be used for snapshot cameras. LED lamps can be used depending on the implemented technology and use case according to the measuring scenario and emitted wavebands [36]

To acquire a proper datacube different calibration routines are needed to ensure highly accurate reflectance values. Figure 3 shows a generalized processing pipeline for hyperspectral cubes for the demands of plant imaging in greenhouses and laboratories as it is common for the demands of plant phenotyping.

Wavelength calibration - from pixel to wavelength

When using a pushbroom sensor one dimension of the detector represents the spatial information of the lines of the target. The other dimension represent the full spectrum of a single line of pixels. The wavelength calibration describes the comparison of measured spectral values with known values [37] and consequently, the mapping of the dispersed geometric axis to wavelength in *nm*.

A calibration is needed after manufacturing and after any physical changes to the optical path [38]. Wavelength calibration is obtained by exposing the optical system to a calibration light source / sources. Three aspects are critical for obtaining a proper wavelength calibration including (i) the selection of the

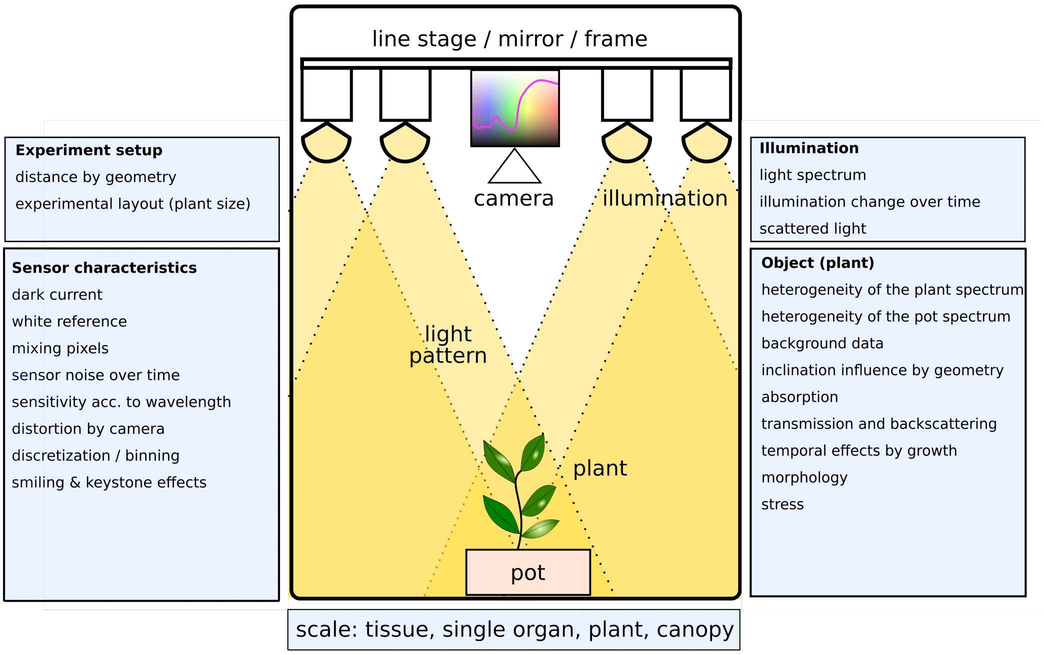

Figure 1. Influences on the measured spectrum of a plant. The four main sources of influence are the experimental setup, the way the camera is mounted, the distance to the plant etc., the light, its spectrum, focus, the object of interest with its absorbing and transmitting properties when imaging plants and the sensor in particular the dark and white referencing, its noise and sensitivity, distortion, discretization and binning.

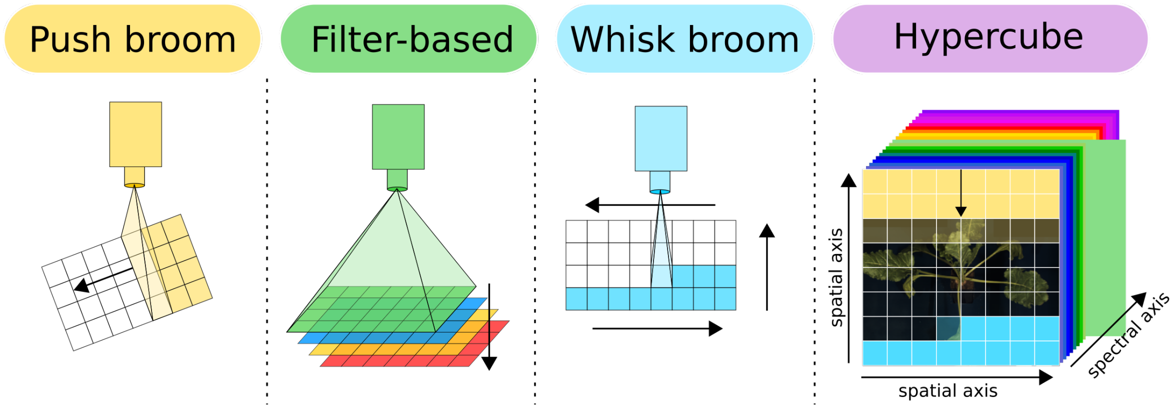

Figure 2. Overview of common HSI techniques: Three different HSI setups are commonly used. Push broom cameras (yellow) are line scanners that were moved over the object or alternatively use a mirror, filter-based systems (green) scan single wavelengths according to the filters one after each other, whisk broom cameras (blue) scan the full spectrum pixel by pixel. All setups result in a 3D hypercube (purple) showing two spatial axis and one spectral axis.

calibration light, (ii) the determination of the center of characteristic peaks and (iii) a polynomial fitting to the data [39]. The calibration light source / sources should cover the wavelength range to be calibrated. Wavelength calibration light sources emit atomic emission lines of known wavelengths. A polynomial fit of the geometric position of the atomic emission lines on the chip and the known wavelength is conducted. This step is usually performed primarily by the manufacturer and enables displaying the spectral axis in units of wavelength (*nm*).

Instrument function / point spread function - overcoming spectral distortion

Measurements of any optical device can be described as a convolution of the original data with the appropriate transfer function of the sensor and optical setup. This convolution is characterized as a (spectral & spatial) blurring or smearing of the data [40]. The terms "instrument function" and "point spread function" are both used to describe this convolution. The term "point spread function" typically refers to the spatial convolution. The term "instrument function" is referring to the convolution in the spectral domain. Both terms define the highest possible spectral and spatial resolution. Effects resulting from the point spread function are described in the following paragraphs. In contrast to spatial distortions the (spectral) instrument function is typically not corrected for.

Spatial calibration - overcoming spatial distortion

Similar to 2D-RGB-cameras which come with barrel and pillow distortion [41], the images of a hyperspectral line scanner tend to show similar effects called smile and keystone effects. Smile is the curvature distortion of the horizontal spectra lines[34] or a shift in wavelength in the spectral domain [42]. Keystone is the distortion of the focal plane rectangle into a trapezoid [34] or a band-to-band misregistration [42]. These effects can be corrected using geometric control points (GCP) [34]. A spatial calibration of the hyperspectral cube describes the character of the spatial mapping process. This process results in an rectified image. Not all manufacturers provide this calibration by default.

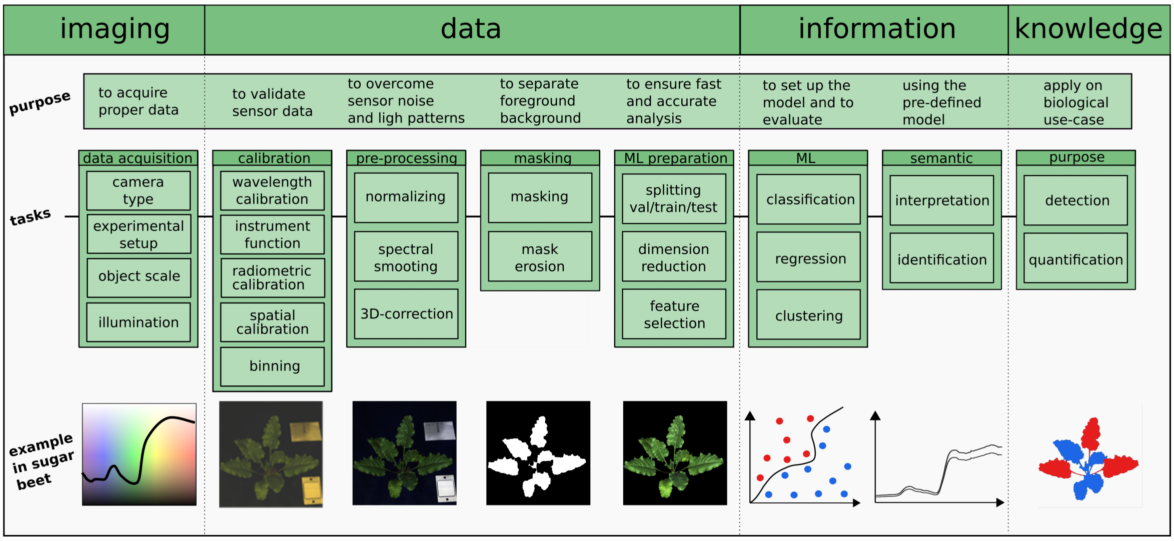

Figure 3. A generalization of a hyperspectral workflow The way to extract information from sensor data and to bring it into a biological context to generate knowledge starts with the data acquisition, the hardware calibration, a proper normalization step, data pre-processing, masking to focus on the object of interest, the plant and to cut out background, plant pot and stabilization sticks etc. Depending on the experiment setup data and the analysis type has to be divided into validation, training and test data set to train a model and to evaluate it on the test data. This is followed by the result interpretation and identification of diseases, stresses or other properties of the plants. Vertical dashed lines describe in a general way the transition between the imaging process, the processing of the data, the generation of information and by interpretation knowledge.

Radiometric calibration - from counts to a physical unit

Due to differences in quantum efficiency of the detector and varying efficiency of the grating and other optical components (lenses etc.), measurements using different optical systems of the same object under same illumination conditions may not be identical [38]. Data-level is influenced by sensor characteristics, atmospheric conditions, and surface properties of the plants. On the most basic level cameras return their measurement values as digital numbers. To correct for such instrument related differences of these returned digital numbers, radiometric calibration of the measurement device or white referencing is needed. Radiometric calibration transforms these digital numbers to radiance values. Radiance depicts the physical measurement of the spectral power flux emitted, received, transmitted or reflected by an object per unit solid angle and projected area. It uses an integrating sphere to measure the calibration coefficients for each wavelength band (pixel) [43].

The camera digital output is mapped to a physical quantity (radiance) using a certified spectral transfer standard (integrating sphere plus calibrated emitter). Thus, radiometric calibration accounts for the spectral variation of the external lens system, internal optics, sensor and dispersive elements (grating and filter). Radiance values are typically used in high altitude / long distance measurement scenarios (plane or satellite based measurements). Radiometric calibration does not account for a potential active illumination light source, atmospheric absorption between the object under study and the camera system as well as surface properties of the specimen. It corrects for the camera and optics spectrally varying efficiency.

Radiance data can be converted to reflectance data if the irradiation source is known or measured [44] In many applications absolute radiometric calibration and the corresponding radiance data is not required. Often, it is sufficient to use reflectance data rather than radiance data. In contrast to radiance data which involves an absolute calibration, reflectance data does not require absolute calibration. A relative spectral calibration to correct for the spectrally varying system efficiency using a simple white reference and dark offset subtraction is sufficient for reflectance measurements. Reflectance data is corrected for camera effects, atmospheric conditions and lighting effects, so only the surface properties of the measured object remain.

Spectral and spatial binning - reducing the noise level

To acquire a high retrieval accuracy within the acquired data a high signal to noise ratio (SNR) is required. SNR is the ratio of the radiance measured to the noise created by the detector and instrument electronics [4]. This ratio can be increased by combining spectral image information along the spectral axis (spectral binning) or by integrating the neighbour pixels (spatial binning) [35]. It was shown that binning along the spectral axis using just a few neighbours reduces of the (spectral) image size in favor of an enhanced signal to noise ratio [45]. Nevertheless, lowest SNR ratio is usually found at the beginning and end of the measurable range of a sensor. A common step to deal with this area is simply cutting the first and last few spectral bands of the sensor [36].

In general, wavelength next to each other are highly correlated [46]. Thus it can be stated, that a limited spectral binning will not affect the informative value of the remaining spectrum.

Binning can be performed directly at the camera internal hardware (hardware binning) or by a processing software when loading the datacube (software binning). In general, hardware binning results in less noise than software binning as the sensor signal is directly merged in the camera prior to analog digital conversion. If using hardware binning, this step has to be performed first before any calibration. If using software binning, it is the first step in the pre-processing right after the hardware calibration steps.

Data preprocessing

Pre-processing can be initiated after hardware calibration and measurement validation. A standardized process is needed to compare measurements from different timepoints and from different measuring setups. The pre-procesing steps include the normalization, the spectral smoothing and 3D correction, masking of the object of interest, data splitting, dimension reduction and feature selection for ML.

reflectance retrieval - overcoming the light source influence

To enable comparable measurements for time series within the same measurement setup, between different sensor setups or under different illumination conditions the normalization of the datacube according to the maximum and minimum reflectance intensity is needed. Therefore the dark image is captured by recording the hypercube with a lid on the camera or a closed shutter. This dark data cube described the lowest possible sensor signal. Right after this the white reference spectrum is acquired using a spectrally known reference target. Most often highly reflective materials like barium sulfate (www.SphereOptics.de, www.labsphere.com) act as a reference. Alternatively the use of materials with a known spectral reflectance across the entire spectral range is established as a standard procedure. Here black, dark and light gray objects can be measured with a point spectrometer to get a known reflectance value. When sharing datasets the reference spectral characteristics should be provided as meta-data to ensure the reusability and comparableness. Performing the object scan right after including the associated dark image, the normalization step can be described by formula 1:

$$I_{Norm} = \frac{cube_O - cube_O^D}{cube_R^W - cube_R^D} \quad (1)$$

Equation 1 follows literature [3] [4] and describes. The numerator describes the subtraction of the measured object cube $cube_O$ and the associated dark current $cube_O^D$, the denominator describes the subtraction of the white reference measurement $cube_R^W$ and the associated dark reference $cube_R^D$. An important feature of Equation 1 is the reduction of nonuniformity caused by either the imaging chip, the illumination or the measuring situaten (box etc.).

For measurements in a greenhouse with a variable environment like a change in light condition, or when measuring time series or measurements that cover a large area it is recommended to use multiple targets or periodical re-calibration of the sensor setup.

spectral smoothing - dealing with peaks and spectral outliers

Based on the assumption that the plant spectrum has a smooth spectrum and peaks covering just one or two bands within the spectrum are the result of outliers and noise the use of soft smoothing algorithms is valid. Most established is the Savitzky-Golay smoothing algorithm [47] for hyperspectral data. [48] showed the applicability for use of 15 centered points and a polynomial of degree 3 for a Specim FX10 camera providing 220 bands within 400 − 1000nm. Furthermore multiplicative signal correction [49] and standard normal variate [50] are well established routines for signal correction.

3D correction - correcting the influence of the sensor-object distance

The measured reflectance on the detector is depending on the reflected light intensity and the distance between sensor and reflection point on the object/plant. For measuring a plant with upper and lower leaves, the distance to the sensor is different for both leaves. This results in differences in the measured intensity. Some publications show the normalization of the spatial distance [18] [51]. A prerequisite for this is an integration of a 3D measuring device in the measuring setup (laserscanner, ultrasound etc.). Depending on the distance, the corrected cube contains equal reflectance values for similar surfaces although the distance to the camera is different by using pixelwise distance normalization.

segmentation masking

Image segmentation is used to partition an image into meaningful parts that have similar features and properties [52]. For the demands of plant phenotyping this usually means the separation from plant and background pixels. This is mostly based on simple vegetation indices or thresholds using a specific wavelength [53]. Further segmentation like the identification of single leaves, or the detection of disease symptoms are focused on later in the workflow pipeline as ML methods are used to tackle this problem.

After masking, the transition between fore- and background is very sharp. Pixels at this transition include parts of both classes and are depicted as "mixed pixels". To overcome the influence of these pixels to the analysis result, these pixels have to be removed. Literature shows that the use of erosion as a binary image processing technique is efficient. A filter element the size of 3x3 pixels is used to shrink the region of the foreground [54]. A negative side effect related to the reduction of foreground data is the possibility of loosing important information which can be used to enhance the data quality.

preparation for ML

Up to this point the datacube consists of hundreds of spectral bands. To detect the specific wavelength that include the biggest impact for the question of interest machine learning is needed. This is also important for a later transfer to multispectral cameras with less spectral bands but with the opportunity to measure in high throughput on the field scale.

To prepare the data for use in a common ML routine, using supervised classification approaches, the dataset is split into three subgroups including the same distribution of groups within the three sets. That means the ratio between the included groups is similar. Set one is called the training set and is used to calculate the model of the ML method like support vector machines (SVM) or decision trees (DT). Set two is called validation dataset and is used for model hyper-parameter tuning. The third set is called the test set and is used to evaluate the performance of the developed model and to calculate a model accuracy. The size of the groups differs with respect to the number available samples. A repeated cross-validation using different splits of the dataset (test and training) is recommended. Dimensionality reduction methods can decrease spectral redundancy and reduce data volume within the dataset. Common techniques are principal component analysis (PCA) [55], feature selection using recursive feature elimination (RFE) [56], ReliefF [57] or correlation-based feature selection [58].

Data analysis and interpretation

hyperspectral traits

Hyperspectral traits can be grouped into different groups, depending of the focus of the data. If the data is coming from a single plant (trait level 1) the datacube can be used to derive very lowly resolved information about the plant such as the plant canopy [59]. If the datacube is segmented into regions including single leaves, disease symptoms or spatially confined areas (ROI, trait level 2), these regions can be compared together. This is commonly done by a classification on pixel (single spectrum) level [60]. Time series measurements are essential for accurate capturing of developing disease symptoms. This leads to the development of hyperspectral dynamics over time (trait level 3) [24] [48]. Hyperspectral datacubes are affected by distance and inclination of the measured object. The correction of the hyperspectral information according to distance and inclination is needed. This can be done by modeling the measuring setup and the occuring errors. It needs the

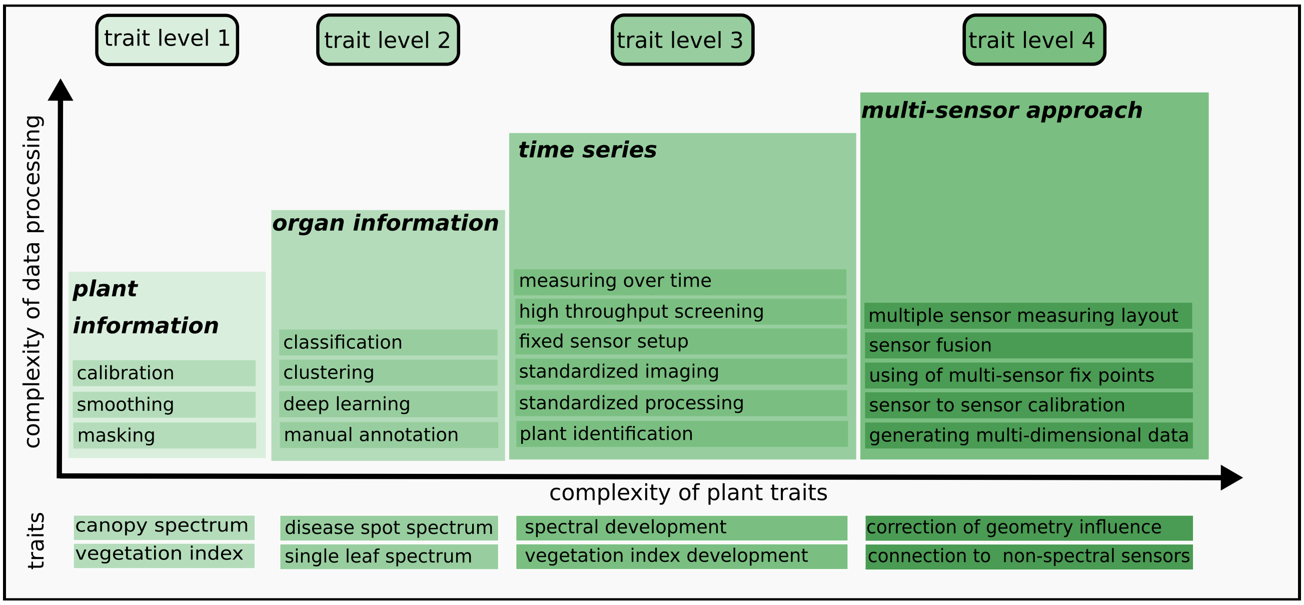

Figure 4. A general trait visualization Plant traits are parameters that describe the hyperspectral properties of the plant tissue. Nevertheless, these traits can be grouped according to the effort that is needed for their extraction. First level (1) traits describe the spectrum of the whole canopy. By using a classification based on ML algorithm it is possible to identify spectra of single leaves (level 2). By measurements over time the development of these spectra can be visualized (level 3) and by using further sensors it is possible to reduce geometrical effects based on a sensor fusion (level 4).

use of an accompanying sensor measuring the object geometry such as a 3D laserscanner [61] [62] and fusing the data for a complete 3D-hyperspectral data model that enables detection of plant disease within a corrected spectrum [33]. An overview about these traits, prerequisites and applications is shown in Fig. 4.

machine learning

For data analysis and ML, the tasks can be divided into supervised methods and unsupervised methods. Supervised methods require a known target value and therefore labeled data to train a model. Within the supervised learning methods, methods can be grouped by their target. If the output is a label as an affiliation to a group and the label is categorial, the method is called classification. Prominent routines for supervised classification are SVM, DT and Neural Network architectures (NN). A similar approach using labeled data is regression, where the output does not predict a group but a numeric value. Known methods for this scenario are Support Vector Regression (SVR), DT and NN.

A special case of ML is Deep Learning (DL). DL allows computational models that are composed of multiple processing layers to learn representations of data with multiple levels of abstraction. It also describes an algorithm allowing raw data as input and automatically discovers a representation, consisting of multiple non-linear modules, for detection or classification [63]. In contrast to SVM or DT approaches, DL is based on NN architectures and depends on huge labelled datasets for training. DL approaches have been widely used on RGB images for the demands of plant phenotyping as a classification of root tips, shoot and leaves [64] [65] and can be depicted to be state of the art. During the last years, hyperspectral applications are raising. Different types of DL approaches have been used for plant disease [66] or stress detection [67].

Usually the results of a classification are presented by a confusion matrix, which indicates for a specific trained model the resulting classification of the test dataset regarding true positive, false positives, false negative and true negative. It compares the predicted values to the true values.

Unsupervised approaches do not need labeled data and try to detect patterns within the data. Clustering approaches like k-means shift manual work from model generation to cluster interpretation as it is the task of the scientist to give semantic to the clustered datasets. The clustering of hyperspectral datasets has been successfully shown for the detection of drought for maize [68].

Challenges and limitations

HSI has to face many challenges regarding sensor setup, illumination, data processing and plant specific characteristics. Starting with the measuring setup where the sensor, illumination and the object distance has to be adapted to the plant size to gain best reflectance results. Thus, the setup has to tailored has to be tailored towards the size of the plants. Both extrema within one measuring setup causes problems in illumination, image resolution and chip intensity.

When extending hyperspectral imaging to the UV area between 200–400*nm*, plants can suffer from the harmful properties of illumination in this spectral region [5]. Further evaluation of the effects of light exposure on the study objects is recommended as plant properties such as architecture, tissue composure and wax layer differ between species.

Surface geometry has a remarkable effect on the measured spectrum. [7] found a correlation between normalized difference vegetation index (NDVI) and surface inclination. Thus this effect has to be taken into account or if possible has to be corrected. This emphasizes the need for imaging setups including different sensors for geometry and reflectance.

The workflow proposed is not transferable to field conditions which requires a very different experimental set up to ensure high quality hyperspectral measurements [69].

High throughput imaging setups [21] combine hyperspectral cameras with high frequent imaging this leads to complex datasets independent of the scale [70]. This emphasized the need for reliable, stable and efficient algorithms and high-end computational machines to process the datacubes. Image analysis and interpretation is the key plant phenotyping bottleneck [71].

Conclusion

HSI is a well-established tool for plant phenotyping in greenhouses. But each laboratory is using a specialized workflow for data assessing, processing and handling which makes the data individually valid but difficult to compare.

This study introduces a generalized workflow for handling hyperspectral image data for greenhouses and laboratories. It includes calibration, reflectance retrieval, data smoothing, masking and preparation for use in a machine learning routine.

This workflow includes hardware-based calibration steps as well as software based processing. Furthermore, a general definition for hyperspectral traits is introduced to establish a level-system starting from traits for the whole plant, to traits for single organs, traits describing temporal development and traits that are based on the measurements of different sensors. An literature overview using hyperspectral imaging and ML is introduced to show the different application areas for plant measuring in agriculture together with the used ML method and the used plant material. Thus a general overview for the application of hyperspectral imaging in plant science is reasonable. This review offers a standardized protocol for raw data processing and how plant traits can be categorized due to their complexity regarding effort in data processing and derivable traits.

Declarations

List of abbreviations

- (A)NN – (artificial) neural network
- CNN – convolutional neural network
- DC – dark current
- DT – decision tree
- (F)LDA – (fishers) linear discriminant analysis
- FNN – fully connected neural network
- GAN – generative adversarial network
- HSI – hyperspectral imaging
- ML – machine learning
- NDVI – normalized difference vegetation index
- NIR – near infrared (700 – 1000*nm*)
- PLS (R) – partial least square (regression)
- RGB – red, green, blue, digital camera sensor
- SAE – stacked auto encoder
- SAM – spectral angle mapper
- SDA – stepwise discriminant analysis
- SNR – signal noise ratio
- SiVm – simplex volume maximization
- SVDD – support vector data descriptor
- SVM – support vector machines
- SWIR – short wave infrared (1000 – 2500*nm*)
- UV – ultra violett spectrum (< 380*nm*)
- VIS – visual spectrum (380 – 700*nm*)
- VNIR – visual + infrared spectrum (380 – 1000*nm*)
- WR – white reference

Ethical Approval (optional)

"Not applicable"

Consent for publication

"Not applicable"

Competing Interests

'The authors declare that they have no competing interests'.

Funding

This study was partially funded by the Deutsche Forschungsgemeinschaft (DFG, German Research Foundation) under Germany's Excellence Strategy - EXC 2070 - 390732324. Furthermore it was supported by Bayer AG - Crop Science.

Author's Contributions

SP and AKM designed the research. AKM supervised the project. SP and AKM designed the review manuscript, prepared the figures and studied the literature. All authors read and approved the final version of the article.

Acknowledgements

We would like to thank Oliver Lischtschenko from Ocean Optics B.V. for his helpful comments and suggestions regarding the hyperspectral sensor calibration. Furthermore we would like to thank Patrick Schramowski for proofreading the machine learning part and Abel Barreto and Anita Kuepper for proofreading and help with the figures.

Authors' information (optional)

References

1. Mahlein AK, Kuska MT, Thomas S, Wahabzada M, Behmann J, Rascher U, et al. Quantitative and qualitative phenotyping of disease resistance of crops by hyperspectral sensors: seamless interlocking of phytopathology, sensors, and machine learning is needed! Current Opinion in Plant Biology 2019 Aug;50:156–162. https://doi.org/10.1016/j.pbi.2019.06.007.
2. Fiorani F, Rascher U, Jahnke S, Schurr U. Imaging plants dynamics in heterogenic environments. Current Opinion in Biotechnology 2012 Apr;23(2):227–235. https://doi.org/10.1016/j.copbio.2011.12.010.
3. Elmasry G, Kamruzzaman M, Sun DW, Allen P. Principles and Applications of Hyperspectral Imaging in Quality Evaluation of Agro-Food Products: A Review. Critical Reviews in Food Science and Nutrition 2012 Nov;52(11):999–1023. https://doi.org/10.1080/10408398.2010.543495.
4. Elmasry G, Sun DW. Principles of Hyperspectral Imaging Technology. In: Hyperspectral Imaging for Food Quality Analysis and Control Elsevier; 2010.p. 3–43. https://doi.org/10.1016/b978-0-12-374753-2.10001-2.
5. Brugger A, Behmann J, Paulus S, Luigs HG, Kuska MT, Schramowski P, et al. Extending hyperspectral imaging for plant phenotyping to the UV-range. Remote Sensing 2019 Jun;11(12):1401. https://doi.org/10.3390/rs11121401.
6. Behmann J, Acebron K, Emin D, Bennertz S, Matsubara S, Thomas S, et al. Specim IQ: Evaluation of a new, miniaturized handheld hyperspectral camera and its application for plant phenotyping and disease detection. Sensors 2018 Feb;18(2):441. https://doi.org/10.3390/s18020441.
7. Behmann J, Mahlein AK, Paulus S, Dupuis J, Kuhlmann H, Oerke EC, et al. Generation and application of hyperspectral 3D plant models: methods and challenges. Machine Vision and Applications 2015 Oct;27(5):611–624. https://doi.org/10.1007/s00138-015-0716-8.

8. Croft H, Chen JM. Leaf Pigment Content. In: Comprehensive Remote Sensing Elsevier; 2018.p. 117–142. https://doi.org/10.1016/b978-0-12-409548-9.10547-0.
9. Corti M, Gallina PM, Cavalli D, Cabassi G. Hyperspectral imaging of spinach canopy under combined water and nitrogen stress to estimate biomass, water, and nitrogen content. Biosystems Engineering 2017 Jun;158:38–50. https://doi.org/10.1016/j.biosystemseng.2017.03.006.
10. Behmann J, Steinrücken J, Plümer L. Detection of early plant stress responses in hyperspectral images. ISPRS Journal of Photogrammetry and Remote Sensing 2014 Jul;93:98–111. https://doi.org/10.1016/j.isprsjprs.2014.03.016.
11. Mahlein AK, Kuska MT, Behmann J, Polder G, Walter A. Hyperspectral Sensors and Imaging Technologies in Phytopathology: State of the Art. Annual Review of Phytopathology 2018 Aug;56(1):535–558. https://doi.org/10.1146/annurev-phyto-080417-050100.
12. Pierna JAF, Vermeulen P, Amand O, Tossens A, Dardenne P, Baeten V. NIR hyperspectral imaging spectroscopy and chemometrics for the detection of undesirable substances in food and feed. Chemometrics and Intelligent Laboratory Systems 2012 Aug;117:233–239. https://doi.org/10.1016/j.chemolab.2012.02.004.
13. Huang M, Wan X, Zhang M, Zhu Q. Detection of insect-damaged vegetable soybeans using hyperspectral transmittance image. Journal of Food Engineering 2013 May;116(1):45–49. https://doi.org/10.1016/j.jfoodeng.2012.11.014.
14. Yang W, Yang C, Hao Z, Xie C, Li M. Diagnosis of Plant Cold Damage Based on Hyperspectral Imaging and Convolutional Neural Network. IEEE Access 2019;7:118239–118248. https://doi.org/10.1109/access.2019.2936892.
15. Zhang SY, Fei T, Ran YH. Diagnosis of heavy metal cross contamination in leaf of rice based on hyperspectral image: a greenhouse experiment. In: 2018 IEEE International Conference on Advanced Manufacturing (ICAM) IEEE; 2018. p. 159–162. https://doi.org/10.1109/amcon.2018.8614938.
16. Nansen C, Zhao G, Dakin N, Zhao C, Turner SR. Using hyperspectral imaging to determine germination of native Australian plant seeds. Journal of Photochemistry and Photobiology B: Biology 2015 Apr;145:19–24. https://doi.org/10.1016/j.jphotobiol.2015.02.015.
17. Moghadam P, Ward D, Goan E, Jayawardena S, Sikka P, Hernandez E. Plant disease detection using hyperspectral imaging. In: 2017 International Conference on Digital Image Computing: Techniques and Applications (DICTA) IEEE; 2017. p. 1–8. https://doi.org/10.1109/dicta.2017.8227476.
18. Reddy KN, Huang Y, Lee MA, Nandula VK, Fletcher RS, Thomson SJ, et al. Glyphosate-resistant and glyphosate-susceptible palmer amaranth (*amaranthus palmeriS. Wats.*): hyperspectral reflectance properties of plants and potential for classification. Pest Management Science 2014 Mar;70(12):1910–1917. https://doi.org/10.1002/ps.3755.
19. Zhang C, Ye H, Liu F, He Y, Kong W, Sheng K. Determination and Visualization of pH Values in Anaerobic Digestion of Water Hyacinth and Rice Straw Mixtures Using Hyperspectral Imaging with Wavelet Transform Denoising and Variable Selection. Sensors 2016 Feb;16(2):244. https://doi.org/10.3390/s16020244.
20. Yu X, Lu H, Liu Q. Deep-learning-based regression model and hyperspectral imaging for rapid detection of nitrogen concentration in oilseed rape (Brassica napus L.) leaf. Chemometrics and Intelligent Laboratory Systems 2018 Jan;172:188–193. https://doi.org/10.1016/j.chemolab.2017.12.010.
21. Ge Y, Bai G, Stoerger V, Schnable JC. Temporal dynamics of maize plant growth, water use, and leaf water content using automated high throughput RGB and hyperspectral imaging. Computers and Electronics in Agriculture 2016 Sep;127:625–632. https://doi.org/10.1016/j.compag.2016.07.028.
22. Rumpf T, Mahlein AK, Steiner U, Oerke EC, Dehne HW, Plümer L. Early detection and classification of plant diseases with Support Vector Machines based on hyperspectral reflectance. Computers and Electronics in Agriculture 2010 Oct;74(1):91–99. https://doi.org/10.1016/j.compag.2010.06.009.
23. Leucker M, Mahlein AK, Steiner U, Oerke EC. Improvement of lesion phenotyping in *cercospora beticola*–sugar beet interaction by hyperspectral imaging. Phytopathology 2016 Feb;106(2):177–184. https://doi.org/10.1094/phyto-04-15-0100-r.
24. Behmann J, Bohnenkamp D, Paulus S, Mahlein AK. Spatial referencing of hyperspectral images for tracing of plant disease symptoms. Journal of Imaging 2018 Dec;4(12):143. https://doi.org/10.3390/jimaging4120143.
25. Nansen C, Sidumo AJ, Capareda S. Variogram analysis of hyperspectral data to characterize the impact of biotic and abiotic stress of maize plants and to estimate biofuel potential. Applied Spectroscopy 2010 Jun;64(6):627–636. https://doi.org/10.1366/000370210791414272.
26. Elvanidi A, Katsoulas N, Ferentinos KP, Bartzanas T, Kittas C. Hyperspectral machine vision as a tool for water stress severity assessment in soilless tomato crop. Biosystems Engineering 2018 Jan;165:25–35. https://doi.org/10.1016/j.biosystemseng.2017.11.002.
27. Moghimi A, Yang C, Miller ME, Kianian S, Marchetto P. Hyperspectral imaging to identify salt-tolerant wheat lines. In: Thomasson JA, McKee M, Moorhead RJ, editors. Autonomous Air and Ground Sensing Systems for Agricultural Optimization and Phenotyping II SPIE; 2017. p. 1182. https://doi.org/10.1117/12.2262388.
28. Pandey P, Ge Y, Stoerger V, Schnable JC. High throughput in vivo analysis of plant leaf chemical properties using hyperspectral imaging. Frontiers in Plant Science 2017 Aug;8. https://doi.org/10.3389/fpls.2017.01348.
29. do Prado Ribeiro L, Klock ALS, Filho JAW, Tramontin MA, Trapp MA, Mithöfer A, et al. Hyperspectral imaging to characterize plant–plant communication in response to insect herbivory. Plant Methods 2018 Jul;14(1). https://doi.org/10.1186/s13007-018-0322-7.
30. Forster A, Behley J, Behmann J, Roscher R. Hyperspectral Plant Disease Forecasting Using Generative Adversarial Networks. In: IGARSS 2019 - 2019 IEEE International Geoscience and Remote Sensing Symposium IEEE; 2019. p. 1793–1796. https://doi.org/10.1109/igarss.2019.8898749.
31. Barreto A, Paulus S, Varrelmann M, Mahlein AK. Hyperspectral imaging of symptoms induced by Rhizoctonia solani in sugar beet: comparison of input data and different machine learning algorithms. Journal of Plant Diseases and Protection 2020;.
32. Berdugo CA, Zito R, Paulus S, Mahlein AK. Fusion of sensor data for the detection and differentiation of plant diseases in cucumber. Plant Pathology 2014 May;63(6):1344–1356. https://doi.org/10.1111/ppa.12219.
33. Roscher R, Behmann J, Mahlein AK, Dupuis J, Kuhlmann H, Plümer L. Detection of disease symptoms on hyperspectral 3D plant models. ISPRS Annals of Photogrammetry, Remote Sensing and Spatial Information Sciences 2016 Jun;III-7:89–96. https://doi.org/10.5194/isprs-annals-iii-7-89-2016.
34. Lawrence KC, Park B, Windham WR, Mao C. Calibration of a pushbroom hyperspectral imaging system for agricultural inspection. Transactions of the ASAE 2003;46(2). https:

//doi.org/10.13031/2013.12940.
35. Rehman SU, Kumar A, Banerjee A. SNR improvement for hyperspectral application using frame and pixel binning. In: Xiong XJ, Kuriakose SA, Kimura T, editors. Earth Observing Missions and Sensors: Development, Implementation, and Characterization IV SPIE; 2016. p. 134 – 139. https://doi.org/10.1117/12.2220599.
36. Mahlein AK, Hammersley S, Oerke EC, Dehne HW, Goldbach H, Grieve B. Supplemental Blue LED Lighting Array to Improve the Signal Quality in Hyperspectral Imaging of Plants. Sensors 2015 Jun;15(6):12834–12840. https://doi.org/10.3390/s150612834.
37. Gaigalas AK, Wang L, He HJ, DeRose P. Procedures for Wavelength Calibration and Spectral Response Correction of CCD Array Spectrometers. Journal of Research of the National Institute of Standards and Technology 2009 Jul;114(4):215. https://doi.org/10.6028/jres.114.015.
38. Yao H, Lewis D. Spectral Preprocessing and Calibration Techniques. In: Hyperspectral Imaging for Food Quality Analysis and Control Elsevier; 2010.p. 45–78. https://doi.org/10.1016/b978-0-12-374753-2.10002-4.
39. Sun YC, Huang C, Xia G, Jin SQ, Lu HB. Accurate wavelength calibration method for compact CCD spectrometer. Journal of the Optical Society of America A 2017 Mar;34(4):498. https://doi.org/10.1364/josaa.34.000498.
40. Kumar VN, Rao DN. Determination of the instrument function of a grating spectrometer by using white-light interferometry. Applied Optics 1997 Jul;36(19):4535. https://doi.org/10.1364/ao.36.004535.
41. Weng J, Cohen P, Herniou M. Camera calibration with distortion models and accuracy evaluation. IEEE Transactions on Pattern Analysis and Machine Intelligence 1992;14(10):965–980. https://doi.org/10.1109/34.159901.
42. Yokoya N, Miyamura N, Iwasaki A. Preprocessing of hyperspectral imagery with consideration of smile and keystone properties. In: Larar AM, Chung HS, Suzuki M, editors. Multispectral, Hyperspectral, and Ultraspectral Remote Sensing Technology, Techniques, and Applications III SPIE; 2010. p. 73 – 81. https://doi.org/10.1117/12.870437.
43. Hruska R, Mitchell J, Anderson M, Glenn NF. Radiometric and Geometric Analysis of Hyperspectral Imagery Acquired from an Unmanned Aerial Vehicle. Remote Sensing 2012 Sep;4(9):2736–2752. https://doi.org/10.3390/rs4092736.
44. Durell C. White paper: Top-of-Atmosphere reflectance calibration of satellite and airborne sensor systems using FLARE vicarious calibration network. North Sutton, NH 03260 USA: Labsphere, Inc; 2016.
45. Polder G, van der Heijden GW. Calibration and characterization of spectral imaging systems. In: Tong Q, Zhu Y, Zhu Z, editors. Multispectral and Hyperspectral Image Acquisition and Processing SPIE; 2001. p. 10 – 17. https://doi.org/10.1117/12.441362.
46. Mahlein AK, Rumpf T, Welke P, Dehne HW, Plümer L, Steiner U, et al. Development of spectral indices for detecting and identifying plant diseases. Remote Sensing of Environment 2013 Jan;128:21–30. https://doi.org/10.1016/j.rse.2012.09.019.
47. Savitzky A, Golay MJE. Smoothing and differentiation of data by simplified least squares procedures. Analytical Chemistry 1964 Jul;36(8):1627–1639. https://doi.org/10.1021/ac60214a047.
48. Bohnenkamp D, Kuska MT, Mahlein AK, Behmann J. Hyperspectral signal decomposition and symptom detection of wheat rust disease at the leaf scale using pure fungal spore spectra as reference. Plant Pathology 2019 Apr;68(6):1188–1195. https://doi.org/10.1111/ppa.13020.
49. Bodner G, Nakhforoosh A, Arnold T, Leitner D. Hyperspectral imaging: a novel approach for plant root phenotyping. Plant Methods 2018 Oct;14(1). https://doi.org/10.1186/s13007-018-0352-1.
50. Asaari MSM, Mishra P, Mertens S, Dhondt S, Inzé D, Wuyts N, et al. Close-range hyperspectral image analysis for the early detection of stress responses in individual plants in a high-throughput phenotyping platform. ISPRS Journal of Photogrammetry and Remote Sensing 2018 Apr;138:121–138. https://doi.org/10.1016/j.isprsjprs.2018.02.003.
51. Huang Y, Lee MA, Nandula VK, Reddy KN. Hyperspectral Imaging for Differentiating Glyphosate-Resistant and Glyphosate-Susceptible Italian Ryegrass. American Journal of Plant Sciences 2018;09(07):1467–1477. https://doi.org/10.4236/ajps.2018.97107.
52. Kaur D, Kaur Y. Various image segmentation techniques: a review. International Journal of Computer Science and Mobile Computing 2014;3(5):809–814.
53. Hillnhütter C, Mahlein AK, Sikora RA, Oerke EC. Use of imaging spectroscopy to discriminate symptoms caused by Heterodera schachtii and Rhizoctonia solani on sugar beet. Precision Agriculture 2011 Jun;13(1):17–32. https://doi.org/10.1007/s11119-011-9237-2.
54. Moghimi A, Yang C, Miller ME, Kianian SF, Marchetto PM. A Novel Approach to Assess Salt Stress Tolerance in Wheat Using Hyperspectral Imaging. Frontiers in Plant Science 2018 Aug;9. https://doi.org/10.3389/fpls.2018.01182.
55. Williams PJ, Kucheryavskiy S. Classification of maize kernels using NIR hyperspectral imaging. Food Chemistry 2016 Oct;209:131–138. https://doi.org/10.1016/j.foodchem.2016.04.044.
56. Guyon I, Weston J, Barnhill S, Vapnik V. Gene Selection for Cancer Classification using Support Vector Machines. Machine Learning 2002;46(1/3):389–422. https://doi.org/10.1023/a:1012487302797.
57. Robnik-Šikonja M, Kononenko I. Theoretical and Empirical Analysis of ReliefF and RReliefF. Machine Learning 2003;53(1/2):23–69. https://doi.org/10.1023/a:1025667309714.
58. Moghimi A, Yang C, Marchetto PM. Ensemble feature selection for plant phenotyping: a journey from hyperspectral to multispectral imaging. IEEE Access 2018;6:56870–56884. https://doi.org/10.1109/access.2018.2872801.
59. Cao X, Luo Y, Zhou Y, Duan X, Cheng D. Detection of powdery mildew in two winter wheat cultivars using canopy hyperspectral reflectance. Crop Protection 2013 Mar;45:124–131. https://doi.org/10.1016/j.cropro.2012.12.002.
60. Bergsträsser S, Fanourakis D, Schmittgen S, Cendrero-Mateo M, Jansen M, Scharr H, et al. HyperART: non-invasive quantification of leaf traits using hyperspectral absorption-reflectance-transmittance imaging. Plant Methods 2015;11(1):1. https://doi.org/10.1186/s13007-015-0043-0.
61. Behmann J, Mahlein AK, Paulus S, Kuhlmann H, Oerke EC, Plümer L. Calibration of hyperspectral close-range pushbroom cameras for plant phenotyping. ISPRS Journal of Photogrammetry and Remote Sensing 2015 Aug;106:172–182. https://doi.org/10.1016/j.isprsjprs.2015.05.010.
62. Paulus S. Measuring crops in 3D: using geometry for plant phenotyping. Plant Methods 2019 Sep;15(1). https://doi.org/10.1186/s13007-019-0490-0.
63. LeCun Y, Bengio Y, Hinton G. Deep learning. Nature 2015 May;521(7553):436–444. https://doi.org/10.1038/nature14539.
64. Pound MP, Atkinson JA, Townsend AJ, Wilson MH, Griffiths M, Jackson AS, et al. Deep machine learning provides state-of-the-art performance in image-based plant phenotyping. GigaScience 2017 Aug;6(10). https://doi.org/10.1093/gigascience/gix083.

65. Mochida K, Koda S, Inoue K, Hirayama T, Tanaka S, Nishii R, et al. Computer vision-based phenotyping for improvement of plant productivity: a machine learning perspective. GigaScience 2018 Dec;8(1). https://doi.org/10.1093/gigascience/giy153.
66. Golhani K, Balasundram SK, Vadamalai G, Pradhan B. A review of neural networks in plant disease detection using hyperspectral data. Information Processing in Agriculture 2018 Sep;5(3):354–371. https://doi.org/10.1016/j.inpa.2018.05.002.
67. Singh AK, Ganapathysubramanian B, Sarkar S, Singh A. Deep Learning for Plant Stress Phenotyping: Trends and Future Perspectives. Trends in Plant Science 2018 Oct;23(10):883–898. https://doi.org/10.1016/j.tplants.2018.07.004.
68. Asaari MSM, Mertens S, Dhondt S, Wuyts N, Scheunders P. Detection of plant responses to drought using close-range hyperspectral imaging in a high-throughput phenotyping platform. In: 2018 9th Workshop on Hyperspectral Image and Signal Processing: Evolution in Remote Sensing (WHISPERS) IEEE; 2018. p. 121 – 138. https://doi.org/10.1109/whispers.2018.8747228.
69. Bohnenkamp D, Behmann J, Mahlein AK. In-Field Detection of Yellow Rust in Wheat on the Ground Canopy and UAV Scale. Remote Sensing 2019 Oct;11(21):2495. https://doi.org/10.3390/rs11212495.
70. Virlet N, Sabermanesh K, Sadeghi-Tehran P, Hawkesford MJ. Field Scanalyzer: An automated robotic field phenotyping platform for detailed crop monitoring. Functional Plant Biology 2017;44(1):143. https://doi.org/10.1071/fp16163.
71. Campbell ZC, Acosta-Gamboa LM, Nepal N, Lorence A. Engineering plants for tomorrow: how high-throughput phenotyping is contributing to the development of better crops. Phytochemistry Reviews 2018 Jul;17(6):1329–1343. https://doi.org/10.1007/s11101-018-9585-x.

Click here to access/download;Personal Cover;CoveringLetter.docx

Dr. Stefan Paulus

Institute of Sugar Beet Research

Holtenser Landstr. 77

37079 Göttingen

Dear Sir or Madam,

Please find attached our revised manuscript "Technical workflows for hyperspectral plant image assessment and processing on the greenhouse and laboratory scale" for submission in GigaScience.

The comments of the reviewers have been very helpful for the authors to understand the problems of possible readers and to improve the quality of the publication. We are very thankful for that. All aspects and concerns have been taken into account. Please find below the answered questions and suggestions.

We hope that the study now is acceptable for publication in your journal.

Kind regards,

Stefan Paulus

Reviewer reports:

Reviewer #1: The submitted manuscript reviewed relevant literature and summarized a general workflow for the analysis of plant hyperspectral images collected in controlled environments. This review could have a great impact to the research community: The general workflow could guide researchers to standardize the data acquisition and processing of plant hyperspectral images for controlled environment studies, help accumulate global research efforts, promote the data sharing, and ultimately advance big data analysis for plant spectral responses and therefore biological understanding. Therefore, the manuscript fits well with the journal's scope and could be of great interest to readers. There are some parts need to be further improved or explained.

1. In my opinion, a unique feature of spectral imaging is the combination of spatial and spectral information for objects rather than the combination of spatial and temporal information, which has been stated by the authors in the first paragraph in Background section.

- We appreciate this suggestion, text has been changed accordingly.

2. Details and explanations are needed for the data acquisition section. While line-scan (pushbroom) systems are widely used, many researchers also used area scanning mode (rarely point scanning, aka whiskbroom, mode) for studying plant spectral responses. To the best comprehensiveness, it would be better to briefly introduce all three scanning modes including basic system setup and pros and cons of using each mode. A figure may be added for the best illustration of the system setups.

- We appreciate this suggestion. A figure showing the different techniques for hyperspectral imaging has been added.

3. Data pre-processing (e.g., reflectance calibration or flat field correction)/meta-data information is utmost important for sharing plant hyperspectral images. Authors may consider to emphasize this importance and provide more information on how to select reference targets. For example, Spectralon targets are generally in good quality with known spectral characteristics, so data collected using this type of reference targets could be directly shared as long as the target model number and manufacturer are provided. In case Spectralon targets cannot be used (due to either cost consideration or spatial limitation), inexpensive alternative references can be used but the reference spectral characteristics should be provided as meta-data to ensure the reusability and comparableness of shared datasets.

- The link to the spectralon manufacturer was added, furthermore the sentence: “When sharing datasets the reference spectral characteristics should be provided as meta-data to ensure the reusability and comparableness.”
- Now this point should be emphasized.

4. Authors may consider use "flat field correction" as the name for the section of "reflectance calibration /normalizing …". An important feature of applying Eq.1. to images is to reduce nonuniformity caused by either the imaging chip, illumination, or both.

- This has been changed accordingly.

5. In the section of "preparation for ML", please consider adjusting the description order as "training", "validation", and "testing", which is logically natural and widely used by research communities. Authors may also consider cite a technical-driven review paper on feature selection. This will help readers to further the understanding and knowledge of the techniques can be potentially used.

- This has been changed accordingly.

6. It would be very interesting and useful if authors could provide a table to list some publicly available datasets that were collected by following the general workflow. This will in turn help the technical community to obtain domain datasets for the development of new tools in the future.

- We really appreciate this suggestion. Nevertheless, community is still lacking of hyperspectral datasets of plants with open/free access. This defines a todo for the future. We hope that this study will give a good basis for publishing a technical proper dataset.

7. There are some repeated words and typos to be carefully checked by the authors. For example: "publications" in the abstract and "bedefined" to "be defined" in the Data acquisition and processing section.

- This has been changed accordingly.

Reviewer #2: This is a review paper focusing on close-range hyperspectral imaging for plant assessment in the greenhouse and laboratory scales. Given the broad interest of using hyperspectral imaging for plant phenotyping research, as well as the complexity of data structure and analysis method, this manuscript is quite timely and relevant. The hyperspectral image is known for its large data volume. The topic thus is appropriate for the journal. The paper covered the topics including camera and measurement setup, data preprocessing, and data analysis/interpretation. The authors' argument is that a standardized workflow for image acquisition, processing and analysis is needed to make the data comparable among various labs, which is a valid point. The paper provides a good technical summary of hyperspectral imaging (such as camera and imaging stage setup, white referencing), and gives a good compilation of its applications on plant assessment that can be useful for the phenotyping research community. My major comments for the authors to consider improving the manuscript are in the following.

Section of spectral smoothing. The authors only discussed Savitzky-Golay method and missed many other methods that are common for spectral preprocessing.
In addition to spectral averaging (binning) that the authors also discussed, other methods like Multiplicative Signal Correction and Standard Normal Variate are also widely used. Other preprocessing such as first and second order derivative are also common. Note Savitzky-Golay can also be used for differentiation. I think you need to mention these methods rather than just Savitzky-Golay.

- We have added these methods together with a literature link.

Preparation for ML. You discussion of calibration set, validation set, and test set are not correct. In machine learning, calibration set is for model calibration (to calibrate model parameters), validation is for model hyper-parameter tuning, and the test set is to evaluate the performance of the developed model. Please make sure you express this correctly. In some implementations, an explicit validation set is not used where model calibration and hyper-parameter tuning are conducted together. In these implementations, test set is also referred to as validation set. I would recommend the authors to read some of the literature on NIRS analysis, as when the images are reduced to the spectrum level, the (pre)processing and analysis share commonalities. There are quite a few publications recently on using VIS-NIR-SWIR for leaf analysis in the context of plant phenotyping. Please study those so you can see calibration/validation schemes and spectral preprocessing.

- The description of the machine learning sets has been changed accordingly.

The explanation following Equation 1 was poor. I cannot understand it. Please revise.

- The explanation has been changed.

There is significant room for the authors to improve the writing and presentation of the manuscript. There are quite a few places where the wording and phrases can be improved. Please see my comments on the attached document.

- Comments in the PDF version of the draft have been inserted and the text changed accordingly.

Reviewer #3: This paper presents a workflow for researchers using hyperspectral imaging for phenotyping applications, specifically based in greenhouses and laboratory settings. This paper is very timely and quite necessary, in my opinion. Overall I think the paper is well organized and presents information that will be very useful for researchers as they design their experimental setups. My background is remote sensing, specifically hyperspectral, so many of my comments and

suggestions are based on lining up the language in this manuscript with the language used in the existing remote sensing literature base. Since remote sensing researchers have been working with hyperspectral since the 1980s, I believe this will allow readers to find established and published methods that can directly apply to plant phenotyping without having to 'reinvent the wheel'. I am also assuming that most of your readers may not be familiar with hyperspectral. Especially since if I were new to hyperspectral for phenotyping, I would start with reading this paper!

General Comments:
* To be technically correct, use the term hyperspectral instead of spectral. RGB imagery is also spectral, but it just happens to be broadband and only three bands.

- This has been changed for the plant imaging sections. For the technical sections we focused on the spectral calibration and the techniques, thus we think the term spectral is here appropriate.

* The camera characteristics and measuring setup section should be broken up into two sections. One for camera characteristics and one for the measuring setup. The camera characteristics description is thorough, but I would like to see more details (or more explicitly stated) on the experimental design or measuring setup. Specifically, the authors could elaborate on the following topics:

- As the authors mention, **illumination** is a significant factor in collecting high-quality data. Not all bulbs will work appropriately - what things do researchers need to know not to have illumination issues? Why should any fluorescent lights be turned off before collection?
- We clearly see that illumination is an highly important facor. Thus an extra section “Using illumination for measuring” has been added to the text.
- Side-view versus nadir image collection - why would you choose one over the other? Why will side view not translate to outdoor image collections?
- This has been added accordingly in the section “Measuring setup”.
- The inclusion of a reference panel (briefly mentioned in a different section) in the scene. Should it be all scenes or a preferred location within a scene?
- This has been added accordingly in the section “Measuring setup”.
- A discussion on the field of view of the camera and how to determine camera height based on the sample being collected and desired spatial resolution.
- This has been added accordingly in the section “Measuring setup”.
- Pushbroom versus integrating cameras
- This has been added accordingly in the section “Camera characteristics”.
- There needs to be a better description of each of the remote sensing data levels. At the moment, the terminology isn't quite correct, and the clarity is missing (Radiometric calibration section). Specifically, it would be important to define digital numbers, radiance, and reflectance data levels. They each have very different factors that influence them and require different corrections.
- This has been added accordingly in the section “Radiometric calibration”.
- Do not use the term normalization for reflectance retrieval. Reflectance calibration is ok, but to match the remote sensing literature, reflectance retrieval would be more accurate.
- This has been changed accordingly.

Specific Comments:
Abstract > Results: "This review describes a general workflow for the assessment and the processing of hyperspectral plant data at the greenhouse scale." I would add greenhouse and laboratory scale since this is the first mention of the measurement scale and it will match the title

- This has been changed accordingly.

Abstract > Conclusions: I would have this start on a new line like Background and Results.

- This has been changed accordingly.

"This publications provides a structured overview on implementing hyperspectral imaging into biological studies."
Publication should be singular. I would also add at the end "at the greenhouse and laboratory scale". This paper would not be useful for outdoor collections with UAV or airborne sensors.

- This has been changed accordingly.

Key Words: Make sure to include hyperspectral.

- This has been changed accordingly.

Key Points: hyperspectral not spectral, needs to be structure for evaluation of what?

- This has been changed accordingly.

"During the last years, spectral sensing of plants has developed as a valuable tool for plant phenotyping [1] [2]."
Rewording - "During recent years, hyperspectral sensing…." I think it is important to say hyperspectral instead of spectral. RGB is also spectral, but it just happens to be broadband.

- This has been changed accordingly.

"The principle of hyperspectral imaging (HSI) is based on the fact that all materials reflect electromagnetic energy in prominent patterns and specific wavelength due to difference of their chemical composition and inner physical structure [3]. Spectroscopy is defined as the method of acquiring and explaining the spectral characteristics of an object regarding light intensity emerging from molecules at different wavelengths to provide a precise fingerprint of an object."

This sentence needs some rewording. This is not the principle of hyperspectral imaging but remote sensing in general. **The difference between hyperspectral and other remote sensing is that hyperspectral is characterized by measuring hundreds of narrow bands in the electromagnetic spectrum.** For any remote sensing sensor, the measured signature is the result of a material's chemical composition and inner/outer physical structure. **It is important to note that the spectral signature is not just the inner leaf, especially since that depends on the part of the electromagnetic spectrum that is measured**. Additionally, it is **important to specify HOW hyperspectral is different than multi-spectral sensors (specifically RGB cameras are mentioned)**. In the paper, a lot of great examples are shown using hyperspectral. Still**, I think the introduction could use one sentence saying why someone would invest the extra time/money/effort into using hyperspectral over an RGB camera.** Lastly, spectroscopy can also be collected with a point spectrometer instead of an imager. There is a whole literature base that uses point spectroscopy for phenotyping, which is not the focus on this paper**. I would add a single sentence acknowledging this difference**. Also, it may not be apparent to readers that spectroscopy equals hyperspectral, and I would say that hyperspectral is more commonly used in the plant sciences literature.

- This has been changed accordingly. We hope that it now fulfills the claims of the reviewer.

"Spectral cameras have become affordable that increase the visible spectrum (400 - 700nm, VIS) of RGB-cameras by the ultra-violet (200 - 400nm, UV,[5]), the near infrared spectrum (700 - 1000nm,NIR, [6]) or even the short wave infrared spectrum (1000 - 2500nm, SWIR, [7])."
This sentence needs rewording. Hyperspectral cameras have become more affordable and as a result, more commonly used? Compared to RGB cameras, they increase the spectral resolution and spectral range?

- This has been changed accordingly.

Reflectance imaging of plants has been related to plant tissue characteristics [9], to detect abiotic stresses [10] or plant diseases [11].
This is the first time the term reflectance is used, and it might be easier for readers who are not familiar with this data type to use hyperspectral instead (until you get a chance to define reflectance in the Data Acquisition and Processing section). This list, as written, suggests these are the only applications of hyperspectral imaging of plants. I would add at the end "among others" to give some flexibility.

- This has been changed accordingly.

"To introduce HSI as a state-of-the-art tool for plant phenotyping a literature overview is presented showing the different biological objectives what hyperspectral sensors are used for in the laboratory and greenhouse scale starting from stress detection and disease classi¬fication to a linking to molecular analysis (QTL analysis) grouped by the introduced level-description."
Suggested rewording - "To introduce HSI as a state-of-the-art tool for plant phenotyping, a literature overview is presented showing the different biological objectives can be achieved with hyperspectral sensors in the laboratory and greenhouse settings including stress detection, disease classi¬fication, and molecular analysis (QTL analysis)."

- This has been changed accordingly and by suggestion of an other reviewer.

"The following paragraph introduced introduces techniques to overcome different ..."
Typos: "The following section introduces techniques ..."

- This has been changed accordingly.

"A comprehensive literature review shows examples for hyperspectral application from biotic stress detection like disease or virus detection, abiotic stress detection like heavy metal or cold stress and plant trait extraction like biochemical traits or leaf water content."
Since this is the start of a paragraph, please include again this is at the greenhouse/laboratory scale.

- This has been changed accordingly.

Table 1: Please include in the caption this is for the greenhouse/laboratory scale. I'm not as familiar with hyperspectral greenhouse studies, but there is only one citation for each of these?

- A describing sentence has been added to the table caption.

"Spectral systems and resulting data differ in the way the camera is calibrated and the data is processed."
These are not the only ways hyperspectral systems differ. As mentioned in the following sections, there are many other factors. Perhaps a more generalized sentence? "Hyperspectral systems and resulting data will vary due to many factors, including camera characteristics, experimental setup, calibration, and data processing.

- This has been changed accordingly.

"… sensor wavelength calibration, the instrument function, the radiometric calibration and spectral and pixel binning."
What is "the instrument function"?

- Instrument function and point spread function need a detailed introduction which has been given in the section “Instrument function / point spread function - overcoming spectral distortion”

"Four categories of factors that influence the measured spectrum of plants can bedefi¬ned."

Add space between be and defined.

- This has been changed.

Also, these four factors are HUGE when collecting hyperspectral data and often result in the most errors or incorrectly interpreted data. I love the figure and that these factors are mentioned, but I think they could use a little more elaboration. How might each factor impact your data? The last sentence starts to address this, but in my opinion, it is too much of a summary of all of them. For example, spectra variability due to differences in genotypes is not caused by the optical configuration but the plant's properties.

- We clearly see this point and its importance. Nevertheless a quantification of the influence of the single error sources is rather complicated and needs test series with high quality calibrated recordings. Thus we hope that the summary approach is sufficient for publication.

Camera characteristics and measuring setup As mentioned in general comments, I believe this section should be split into two, which would allow authors to go into detail about how the measurement setup is critical for high-quality measurements. As I progress through specific comments, I will highlight sections that could be expanded on or moved to the measurement set up section.

- The section has been split and changed accordingly.

"Hyperspectral cameras for plant phenotyping often are line scanners (pushbrooms) as this type of sensor is commonly used in plant science or for high throughput analysis as it, unlike snapshot cameras, provides a very high spatial and spectral resolution."
This sentence is awkward and could use rewording. While they are often line scanners there are other hyperspectral camera systems. Since this is a literature review, mention those scanners and how they are different. In the measurement setup section, the pros/cons of each could be explained.

- This has been changed. Furthermore a complete new figure has been added.

"The next step, the transfer of these sensor types to the field scale has already been started for tracking the canopy development in cereals [37] or as an open-source and open data project of Terra-Ref [38]."
My remote sensing background has significant issues with this sentence. Hyperspectral data collection has been happening for decades with airborne sensors or point spectrometers for plant applications. Including predicting nitrogen content and canopy development. Since this sentence doesn't add to the camera characteristics section, I would remove it or reword so that it doesn't exclude a whole body of research (which is outside the scope of this paper).

- This has been changed. The sentence has completely been removed.

Wavelength calibration: I'm quite confused by this section. The wavelengths that sensor measures should be set by the manufacturer. Are there enough people creating their own hyperspectral sensors for this section to be applicable? In my experience, wavelengths rarely drift, and if they do, the manufacturer would prefer to do the correction. The sentence "The wavelength calibration describes the comparison of measured spectral values with known values [40] and consequently, the mapping of the dispersed geometric access to wavelength in nm." sounds like it is discussing reflectance retrieval, but that is a different section. The sentences "A polynomial ¬fit of the geometric position of the atomic emission lines on the chip and the known wavelength is conducted. This step is usually performed preliminary by the manufacturer and enables displaying the spectral

axis in units of wavelength (nm)." Sounds like you are discussing the conversion of digital numbers to radiance, but that is also another section. I've also never heard the term dispersed geometric access, so it would probably be good to define? Now, it is important to know that each band has a spectral response function (again generally provided by sensor manufacturer or estimate by Gaussian function). This information is critical to resample a camera to another camera spectral resolution.

- We agree with this comment. A wavelength calibration should be performed by the manufacturer. The scope of this publication is to introduce all aspects of hyperspectral calibration. After a longer discussion with a specialist of a worldwide spectrometer manufacturer we can say that the majority of the users, especially scientists, build hyperspectral cameras, especially push broom and whisk broom systems themselves and thus need to perform their sensor wavelength calibration by themselves.
- The sentence has been changed access → axis. The confusion should be solved now.
- We deeply re-discussed this text section and think that we could improve the quality and readability. This passage only focusses on mapping of pixel position on the camera to wavelength and not about reflectance or radiance measuring.

"Due to differences in quantum efficiency of the detector and varying efficiency of the grating and other optical components (lenses etc.), measurements using different optical systems of the same object under same illumination conditions may not be identical [41]."
A sentence needs to follow this one that spells out to the reader that this data level is called digital numbers. This data-level is influenced by sensor characteristics, atmospheric conditions, and surface properties (in this example plants). This will emphasize the reason why sensors at this data type level are not comparable.

This has been changed accordingly.

"To correct for such instrument related differences, radiometric calibration of the measurement device or white referencing is needed."
* White reference is NOT used for radiometric calibration. Many software programs will incorporate the radiance to reflectance step into one which would use the white reference. However, the term white referencing is specifically for converting to reflectance. This is a critical difference when making measurements outdoors, but it worth separating here.
This has been changed accordingly.
* It is also important to tell the reader what the radiance is, especially since there are many plant applications (such as photosynthetic studies) that require radiance values, not reflectance. This data product is influenced by the light source, atmospheric absorptions, and surface properties, but it does remove camera factors.
Now it should be more clear and more explicit.
* To convert from DNs to radiance, a gain and offset per band are applied to the data which are provided by the sensor designers or engineers. Software provided by the manufacturer should have those values automatically provided. IF they don't, then you have to develop them yourself, which is the description actually provided in this section.
"In many applications absolute radiometric calibration is not required. Often it is sufficient to use a relative spectral calibration to correct for the spectrally varying system efficiency. A simple white referencing and dark subtraction is sufficient for reflectance measurement."
This needs to be reworded. Right now, it jumps from radiometric calibration to a reflectance measurement - which has not been defined. Also, this depends on the camera system. Often it is possible to 'skip' the radiance conversion because it a linear regression with DNs, but this is not the case with every camera (depending on the camera characteristics it can be non-linear spectrally and spatially).
This has been changed accordingly. And a few more explicit sentences have been added.

Spectral and spatial binning: Yes, SNR can be increased when data is binned, but many new users will do this incorrectly. For example, many hyperspectral sensors have 'bad bands' towards the upper and lower range of the sensor. Bad bands are those defined with having very high noise and unreliable measurements. These bad bands are lower SNR ratio than other bands because they are at the upper limits of the sensor's capabilities. There can also be bad bands due to atmospheric conditions, which in a greenhouse with high water vapor could be strong. I also feel like this is not necessary for all cameras and really depends on the SNR of the camera used. My suggestion would be to word this as an optional step and explain when a user should consider these methods. Especially since there are sections on dimensionality reduction and spectral smoothing which also impacts the spectral data.

This has been changed. A new sentence dealing with the lower and upper spectral area and how to handle it has been added.

"Thus it can be stated, that a slightly spectral binning will not affect the informative value of the remaining spectrum."

Slightly? I think a different word might more appropriate.

- This has been changed to “limited” we hope that this is fine now.

"It includes pre-processing steps where the normalization is performed, the spectral smoothing and 3D correction up to a masking of the object of interest and data splitting, dimension reduction and feature selection for ML."

This sentence is awkward and could use rewording.

- The sentence has been changed.

Reflectance Calibration: Do not use the term normalization for reflectance retrieval. Define what the reflectance data level is and what the units are. It is important for the readers to know that this data level removes camera effects, atmospheric conditions, and lighting effects, so only the surface properties remain. THIS data source is comparable across camera systems, whereas the other data levels are not.

- The term normalization has been changed in the complete study article. Furthermore a more appropriate definition has been added.

"Most often highly reflective materials like barium sulfate (SphereOptics.com) act as a reference." In my opinion, the reference panel is one of the most critical components of making high-quality hyperspectral measurements. I would love to see this in an experimental setup section with a lot more details. For example, the material does need to be highly reflective but also highly reflective across the entire spectral range of the camera measures. Also, probably the most commonly used panel (but of course more expensive) is a spectralon panel made by Labsphere. White paint for camera measuring 400-1000 nm can also be sufficient.

- Spectralon has been added as well as “across the entire spectral range”

"Alternatively the use of materials with a known spectral reflectance is established as a standard procedure."

Yes! I always recommend a black, light gray, and dark gray target also. These can be measured with a point spectrometer to get a known reflectance value.

- This sentence has been added to the text.

"Performing the object scan right after including the associated dark image, the normalization step can be described by formula 1:"

This formula is the most basic way of converting from radiance to reflectance, using only a single target. In the remote sensing literature, it is referred to as the empirical line correction method. However, if you have a variable atmosphere (such as a greenhouse with fluctuating values) or are covering a large area, a single target may not be sufficient for good data. This is also true if the lighting conditions change or if the data set is a time series. A more advanced empirical line correction method incorporates multiple targets, which can make it robust to these changes. Conversion to reflectance from radiance generally results in the largest data errors, so in my opinion is worth elaborating.

- To emphasize this point a new sentence was added.
- "For measurements in a greenhouse with a variable environment like a change in light condition, or when measuring time series or measurements that cover a large area it it recommended to use multiple targets or periodical re-calibration of the sensor setup."

"Based on the assumption that the plant spectrum has a smooth spectrum and peaks within the spectrum are results of outliers and noise the use of soft smoothing algorithms is valid."
This sentence needs to be clarified. Plant spectra can have peaks or valleys that are due to biophysical or structural conditions that people may be interested in. Very sharp peaks that only span one or two wavelengths are definitely noise. This is where a discussion of 'bad bands' that I mentioned before would be useful. Again, this may not apply to all cameras and it may not apply to the whole spectrum depending on the SNR.

- We see this point and appreciate this indication. Thus the part "covering just one or two bands" was added to clarify this.

"Most established is the Savitzky-Golay smoothing algorithm [49] for hyperspectral data where 15 centered points and a polynomial of degree 3 has shown its applicability [50]."

This is highly dependent on the camera's spectral resolution.

- That is right. The sentence has been changed and the camera of the example has been added.

"Literature shows that an the use of erosion as a binary image processing technique is efficient."
Typo shown in italics. There should be a citation with this statement or is it the same as the following sentence? Might be worth mentioning that some machine learning algorithms are robust to them anyway. Also, I'm sure you are aware there is a whole literature for working with mixed pixels which might be helpful for readers to know if their spatial resolutions are coarse.

- The authors think that Moghimi 2018 should be enough as a citation. The fact that some but not all methods are robust to mixed pixels is right, but we think that this will be beyond the scope of this study as we do not want to focus on ML techniques. But we are thankful for this comment.

Preparation for ML: I love that the authors chose to focus on machine learning techniques. I have found too many phenotyping papers that rely on a vegetation index to retrieve their trait of interest. Why do we have cameras that measure hundreds of bands if we are going to reduce them down to one value? I would love to see one sentence on why researchers should use ML approaches rather than a vegetation index.

- This has been changed. A short paragraph has been added in the beginning of "preparation for ML"

"To decrease redundancy within the dataset dimension reduction as it can be performed."

I would like to suggest a clarification for this sentence - "Dimensionality reduction methods can decrease spectral redundancy and reduce data volume within the dataset."

- This has been changed accordingly.

"State-of-the-art techniques are principal component analysis (PCA, [55]), feature selection using recursive feature elimination (RFE), ReliefF or correlation-based feature selection [56]."

I would change "state-of-the-art" to common since PCA was one of the very first dimensionality reduction techniques. Or split it into two sentences - one with common and another with new algorithms.

- This has been changed.

"If the data is coming from a single plant (trait level 1) the datacube can be used to derive very rough information about the plant like the plant canopy [57]."

Very rough information? What does this mean? Instead of like, I would suggest "such as"

- This has been changed. Rought -> low resolved

"The correction of thehyperspectral information according to distance and inclination is needed."

Space needed between the and hyperspectral.

- This has been changed.

"In contrast to SVM or DT approaches, DL is based on N architectures and is based on very huge datasets used for training."

Consider rewording to remove duplicate "is based on"

- This has been changed.

"DL approaches have been widely used on RGB images for the demands of plant phenotyping as there is a classi¬fication of root tips, shoot and leaves [61] [62] and can be depicted to be state of the art."

Remove there is.

- This has been changed.

"Usually the results of a classi¬fication are presented by a confusion matrix, which indicates…"
Since the previous sentence said no labeled data was needed, it might be worth mentioning that the confusion matrix does need labeled data.

- This is right, nevertheless, the labeled data is needed for evaluation. To clarify this, this paragraph was moved to the supervised section.

"Thus, the setup has to tailored has to be tailored towards the size of the plantss."

Remove duplicate s on plants.

- This has been changed.

"Beside effects of the geometry, like the correlation between normalized difference vegetation index (NDVI) and inclination, have to be taken into account or if possible have to be corrected [7]."
NDVI is not only influenced by leaf inclination but also more broadly canopy structure.

- The text clearly says that the datacube is affected by distance and inclination which includes effects of the canopy structure. The authors think an additional emphasizing of this aspect is not necessary. Thus, the text was not changed .

"When transferring results from the laboratory or greenhouse to the ¬field the work ow for using HSI is different and has to be designed individually [66]."
I think this paragraph should be condensed significantly since it is definitely out of the scope of the paper and a single paragraph would not be sufficient to explain how this workflow would be transferrable to field settings. The reflectance retrieval process (referred to as normalization here) is completely different for field collections. As mentioned most everything is different. I would

summarize by saying "The workflow proposed is not transferrable to field conditions which requires a very different experimental set up to ensure high quality hyperspectral measurements."

- This has been changed.

"Especially when using high throughput imaging setups [21] combined with hyperspectral cameras periodical imaging leads to huge datasets independent of the scale [37]."

This sentence is should be reworded.

- This has been changed.
