# Supplementary material for: Technical workflows for hyperspectral plant image assessment and processing on the greenhouse and laboratory scale
Source: Gigascience. 2020 Aug 20;9(8):giaa090. doi: 10.1093/gigascience/giaa090 (PMC7439585; doi:10.1093/gigascience/giaa090)
Supplement: giaa090_GIGA-D-20-00091_Revision_2 [file giaa090_giga-d-20-00091_revision_2.pdf]

GigaScience

Technical workflows for hyperspectral plant image assessment and processing on the greenhouse and laboratory scale

--Manuscript Draft--

**Manuscript Number:**	GIGA-D-20-00091R2
**Full Title:**	Technical workflows for hyperspectral plant image assessment and processing on the greenhouse and laboratory scale
**Article Type:**	Review
**Funding Information:**	Deutsche Forschungsgemeinschaft (EXC 2070 - 39073232) — Prof. Dr. Anne-Katrin Mahlein<br>Bayer CropScience — Prof. Dr. Anne-Katrin Mahlein
**Abstract:**	Using hyperspectral cameras is well established in the field of plant phenotyping, especially when using high throughput routines in greenhouses. Nevertheless, the used workflows differ depending on the applied camera, the imaged plants, the experience of the users and the measuring setup.<br>This review describes a general workflow for the assessment and processing of hyperspectral plant data at greenhouse and laboratory scale. Aiming at a detailed description of possible error sources, a comprising literature review of possibilities to overcome these errors and influences is provided. The processing of hyperspectral data of plants starting from the hardware sensor calibration, the software processing steps to overcome sensor inaccuracies and the preparation for machine learning is shown and described in detail.<br>Furthermore, plant traits extracted from spectral hypercubes are categorized to standardize the terms used when describing hyperspectral traits in plant phenotyping. A scientific data perspective is introduced covering information for canopy, single organs, plant development and also combined traits coming from spectral and 3D measuring devices.<br>This publication provides a structured overview on implementing hyperspectral imaging into biological studies at greenhouse and laboratory scale. Workflows have been categorized to define a trait level scale according to their metrological level and the processing complexity.<br>A general workflow is shown to outline procedures and requirements to provide fully calibrated data of highest quality. This is essential for differentiation of smallest changes from hyperspectral reflectance of plants, to track and trace hyperspectral development as an answer to biotic or abiotic stresses.
**Corresponding Author:**	Stefan Paulus, Ph.D.<br>Institute of Sugar Beet Research<br>Göttingen, GERMANY
**Corresponding Author Secondary Information:**	
**Corresponding Author's Institution:**	Institute of Sugar Beet Research
**Corresponding Author's Secondary Institution:**	
**First Author:**	Stefan Paulus, Ph.D.
**First Author Secondary Information:**	
**Order of Authors:**	Stefan Paulus, Ph.D.<br>Anne-Katrin Mahlein, Professor
**Order of Authors Secondary Information:**	
**Response to Reviewers:**	The comments of the reviewers have been very helpful and have been completely included into the manuscript. Furthermore the language has been checked by a native speaker.
**Additional Information:**	

Powered by Editorial Manager® and ProduXion Manager® from Aries Systems Corporation

Question	Response
Are you submitting this manuscript to a special series or article collection?	No
**Experimental design and statistics**<br><br>Full details of the experimental design and statistical methods used should be given in the Methods section, as detailed in our Minimum Standards Reporting Checklist. Information essential to interpreting the data presented should be made available in the figure legends.<br><br>Have you included all the information requested in your manuscript?	Yes
**Resources**<br><br>A description of all resources used, including antibodies, cell lines, animals and software tools, with enough information to allow them to be uniquely identified, should be included in the Methods section. Authors are strongly encouraged to cite Research Resource Identifiers (RRIDs) for antibodies, model organisms and tools, where possible.<br><br>Have you included the information requested as detailed in our Minimum Standards Reporting Checklist?	Yes
**Availability of data and materials**<br><br>All datasets and code on which the conclusions of the paper rely must be either included in your submission or deposited in publicly available repositories (where available and ethically appropriate), referencing such data using a unique identifier in the references and in the "Availability of Data and Materials" section of your manuscript.	Yes

Powered by Editorial Manager® and ProduXion Manager® from Aries Systems Corporation

Have you have met the above requirement as detailed in our Minimum Standards Reporting Checklist?	

Powered by Editorial Manager® and ProduXion Manager® from Aries Systems Corporation

Manuscript Click here to access/download;Manuscript;main.tex

Warning. The length \marginparwidth is less than 2cm and will most likely cause issues with the appearance of inserted todonotes. The issue can be solved by adding a line like \setlength{\marginparwidth}{2cm} prior to loading the todonotes package.

GigaScience, 2017, 1–12

doi: xx.xxxx/xxxx
Manuscript in Preparation
Review

REVIEW

Technical workflows for hyperspectral plant image assessment and processing on the greenhouse and laboratory scale

Stefan Paulus[1,*] and Anne-Katrin Mahlein[1,†]

[1]Institute of Sugar Beet Research, Holtenser Landstr. 77, 37079 Göttingen, Germany

[*]paulus@ifz-goettingen.de;
[†]mahlein@ifz-goettingen.de

Abstract

Background: Using hyperspectral cameras is well established in the field of plant phenotyping, especially when using high throughput routines in greenhouses. Nevertheless, the used workflows differ depending on the applied camera, the imaged plants, the experience of the users and the measuring setup.
Results: This review describes a general workflow for the assessment and processing of hyperspectral plant data at greenhouse and laboratory scale. Aiming at a detailed description of possible error sources, a comprising literature review of possibilities to overcome these errors and influences is provided. The processing of hyperspectral data of plants starting from the hardware sensor calibration, the software processing steps to overcome sensor inaccuracies and the preparation for machine learning is shown and described in detail.
Furthermore, plant traits extracted from spectral hypercubes are categorized to standardize the terms used when describing hyperspectral traits in plant phenotyping. A scientific data perspective is introduced covering information for canopy, single organs, plant development and also combined traits coming from spectral and 3D measuring devices.
Conclusions This publication provides a structured overview on implementing hyperspectral imaging into biological studies at greenhouse and laboratory scale. Workflows have been categorized to define a trait level scale according to their metrological level and the processing complexity. A general workflow is shown to outline procedures and requirements to provide fully calibrated data of highest quality. This is essential for differentiation of smallest changes from hyperspectral reflectance of plants, to track and trace hyperspectral development as an answer to biotic or abiotic stresses.

Key words: plant phenotyping, camera calibration, machine learning, hyperspectral signature, hyperspectral

Background

During recent years, hyperspectral sensing of plants has developed as a valuable tool for plant phenotyping [1] [2]. The principle of hyperspectral imaging (HSI) is based on the fact that all materials reflect electromagnetic energy in prominent patterns and specific wavelength due to difference of their chemical composition, inner physical structure and surface properties. This signal is characterized by measuring hundreds of narrow bands within the electromagnetic spectrum [3]. Spectroscopy is defined as the method of acquiring and explaining the hyperspectral characteristics of an object regarding light intensity emitted, reflected or transmitted from molecules at different wavelengths to provide a precise fingerprint of an object. Hyperspectral imaging combines spectral and spatial information similar to a digital camera [4]. Hyperspectral Imaging extends the measurable spectral range from the visible (RGB camera) to the NIR range and sample the spectrum in a large number of narrow bands (> 20 bands). If only a few (< 20) spectral bands were sampled literature depicts this as multispectral. Compared to spectroscopy, which measures the same spectral area, HSI is able to measure spectral and spatial infor-

Compiled on: July 30, 2020.
Draft manuscript prepared by the author.

Key Points

- a literature overview is provided to describe aims and scopes of hyperspectral sensing of plants and the different types of analysis methods
- hyperspectral workflows for plant measuring are highly specific and need to be structured for result comparison and evaluation
- a general workflow for hyperspectral plant phenotyping including camera calibration, segmentation and machine learning analysis is shown
- a level-based trait definition is introduced for canopy, plant organ, time series and sensor fusion

mation in an image which enables a more detailed analysis of the object.

Hyperspectral cameras have become affordable during the last years. Unlike RGB cameras that image the visible spectrum (400 – 700*nm*, VIS) this area is extended by the ultra-violet (200 – 400*nm*, UV,[5]), the near infrared (700 – 1000*nm*, NIR, [6]) or even the short wave infrared spectrum (1000 – 2500*nm*, SWIR, [7]). This is highly interesting for plant science as many plant traits and biophysiological processes can be traced beyond the visible spectral range [8]. Hyperspectral imaging of plants has been use to measure plant tissue characteristics [9], to detect abiotic stresses [10] or plant diseases [11] among others.

Typically, laboratory workflows differ in their use of cameras, measuring setups and data handling such as calibration, smoothing and segmentation. There are several hardware calibration steps to understand and execute, starting from the camera pixel position mapping to the proper wavelength, the correction of the camera and lens distortion to the correction of the 3D setup when measuring upper and lower leaves of a plant. Thus, an introduction of a standardized workflow of hyperspectral image processing is needed to enable the comparison of results from different laboratories regarding their hyperspectral analysis.

To introduce HSI as a state-of-the-art tool for plant phenotyping, a literature overview is presented showing the different biological objectives what hyperspectral sensors are used for in the laboratory and greenhouse scale. The overview comprises stress detection, disease classification and a link to molecular analysis (QTL analysis). All found use-cases were grouped by the introduced level description.

The following section introduces techniques to overcome different impairments on the measured spectrum coming from the experimental setup, the sensor, the role of illumination and the challenges when measuring complex plants with plant specific optical properties. The complete workflow from sensor adjustment, correction, calibration, segmentation to the extraction of hyperspectral plant traits and to a deeper analysis using routines of machine learning (ML) to extract biological information is described.

The application part describes the different aspects of plant traits based on HSI. Finally, a level-description model is introduced from the perspective of a data scientist. It describes the increase of complexity in data acquisition and data handling, when switching from an averaged spectrum of the plant canopy to an organ-specific spectrum to spectral development in time course to multi-sensor plant models. The latter is needed for the geometrical correction of the spectral data.

HSI a tool for plant screening

A comprehensive literature review shows examples for hyperspectral application from biotic stress detection like disease or virus detection, abiotic stress detection like heavy metal or cold stress and plant trait extraction like biochemical traits or leaf water content at greenhouse and laboratory scale. Table 1 emphasizes different use-cases from plant science, where hyperspectral imaging cameras were used to differentiate between different situations.

In Table 1 hyperspectral data was grouped by trait level which describes the complexity of the traits. Starting from simple image analysis (level 1), to organ identification (level 2), to time series (level 3) and to a final multi-sensor data acquisition (level 4). It is shown that HSI is used for classification and regression problems across all trait levels (1-4). A closer introduction into these phenotypic trait levels can be found below in the text.

Three main groups can be identified including i) detection and quantification of biotic stress like disease detection [11], ii) detection and quantification of abiotic stress like heavy metal [15] or water stress [26] and iii) extraction of plant traits to describe water content [21] or biochemical traits [28].

Thus, HSI is widely used for different aspects of plant screening and can be depicted to be a state-of-the-art tool for plant phenotyping.

Data acquisition and processing

Hyperspectral systems and resulting data will vary due to many factors, including camera characteristics, experimental setup, calibration, environmental characteristics and data processing. This leads to inconsistencies regarding the data quality and the validity of results. This increases the difficulty to compare data from different sensors. Multiple steps are needed to acquire valid physical reflectance data starting from the sensor wavelength calibration, the instrument function, the radiometric calibration and spectral and pixel binning.

The goal of calibration is to standardize the spectral axis, to determine if the sensor is working properly, to provide the accuracy of the extracted data, to validate the credibility and to quantify the instrument errors, accuracy and reproducibility under different operating conditions [4].

Four categories of factors that influence the measured spectrum of plants can be defined (see Figure 1). I) the experimental setup including the optical configuration II) the sensor characteristics including sensor offset, noise and sensitivity behaviour and distortion effects [34] III) the illumination effects from the light source when using active illumination or the surrounding light when using environmental light and IV) the object and its properties. Plant object properties means spectral variability due to differences in genotypes, plant organs, materials within the image such as pot and background data, inclination influence due to the architecture of plants, absorption, transmission and backscattering due to plant tissue properties and temporal variation due to growth.

Table 1. Hyperspectral imaging is widely used for detection of biotic and abiotic stresses as well as for trait description. Traits are categorized by a complexity description starting from trait level 1 (TL1, whole plant), trait level 2 (TL2, organ specific traits), trait level 3 (TL 3, time series) and trait level 4 (TL4, multi sensor traits). The following overview describes a representative selection for the greenhouse and laboratory scale

purpose	group	plant	method	trait level	target	reference
detection of impurities in seeds	traits	wheat, spelt, barley	SVM	TL 1	classification	[12]
insect damage detection	biotic stress	soybean	SVDD	TL 1	classification	[13]
cold stress detection	abiotic stress	maize	CNN	TL 1	regression	[14]
heavy metal stress detection	abiotic stress	rice	SVM	TL 1	classification	[15]
germination detection	traits	trees	LDA	TL 1	classification	[16]
virus detection	biotic stress	tomato, tobacco	SVM	TL 1	classification	[17]
weed resistance analysis	traits	amaranth	FLDA	TL 1	classification	[18]
ph-value determination	traits	rice & water hyacinth	PLS & NN	TL 1	regression	[19]
nitrogen concentration	traits	oilseed rape	SAE & FNN	TL 1	regression	[20]
leaf water content	traits	maize	PLSR	TL 1	regression	[21]
disease detection	biotic stress	sugar beet	ANN, DT, SVM	TL 2	classification	[22]
disease resistance & QTL analysis	biotic stress	sugar beet	SAM	TL 2&3	classification	[23]
disease development	biotic stress	wheat	DT	TL 2&3	classification	[24]
biomass & biofuel potential	traits	maize	SDA	TL 3	classification	[25]
water stress detection	abiotic stress	tomato	DT	TL 3	classification	[26]
salt stress detection	abiotic stress	wheat	SiVm	TL 3	classification	[27]
biochemical trait analysis	traits	maize, soybean	PLSR	TL 3	regression	[28]
detection of plant communication	traits	maize	LDA	TL 3	classification	[29]
disease forecast	biotic stress	barley	GAN	TL 3	classification	[30]
disease early detection	biotic stress	sugar beet	SVM, PLS, DT	TL 3	classification	[31]
disease differentiation	biotic stress	cucumber	SDA	TL 4	classification	[32]
disease detection	biotic stress	sugarbeet	SVM	TL 4	classification	[33]

Camera characteristics

HSI can be performed using three different sensor types: the push broom / line scanner, the filter-based sensor setup and a whisk broom setup (see Figure 2). Push broom cameras scan the region below the sensor in lines and complete the full scan by either moving the sensor [28] or by using a mirror that is panned over the object of interest. A filter based system is measuring the complete region of interest using different filters either by splitting the scan ray using prisma or by using a combined filter pattern. Whisk broom sensors measure the full spectral range pixel by pixel similar to a spectrometer that is moved over the region of interest. All three setups result in a three-dimensional hypercube showing two spatial axis and one spectral axis.

Whisk broom sensors have more moving parts and thus are likely to wear out. Push broom cameras have less moving parts but need a high quality calibration as the different regions of the chip can show different sensitivity which can result in stripes within the datacube. Filter based systems are commonly restricted by the number of filters and provide less spectral resolution. Currently state-of-the-art plant phenotyping centers use mostly push broom line scanners.

Measuring setup

Choosing the right camera for a sensor setup has to take into account the point of interest, side-view or from top-view setup, depending on if one single image from top is sufficient or if multiple images by rotating the plant are needed. Furthermore the spectral region of interest depending on the camera chip (silicon for 380 – 1000*nm*, indium-gallium-arsenide for 1000 – 2500*nm*), the focal length, the minimum working distance, the maximum resolution resulting from sensor height and plant height, the focused signal-to-noise ratio, dynamic range, spectral and spatial resolution, pixel size, frame rate, lenses and operating temperature [35]. In general, the field of view should cover the complete plant from small seedlings to the bigger plants in a time series experiment. This is accompanied by a periodical adaption of the focal plane as the plant height is changing due to plant development. Here the desired resolution has to be considered as the ratio between plant pixels and background pixels is changing continuously. For reference panels the options are a permanent reference measurement after each plant if using a box design, referencing within the measurable volume at the same height as the majority of the plant pixels or a periodical referencing along the scan axis when using a measuring setup at a longer line stage. More information about reference panels can be found in Section "Data preprocessing – reflectance retrieval"

Illumination for measuring

Illumination is essential for HSI, but not every light source can be used. The use of passive light like sunlight which is available outdoors and in greenhouses is preferred. Some types of greenhouse glass can alter the sunlight spectrum if any coatings or special glass is used. Active light sources need a closer consideration. Tungsten halogen lamps are broad band emitter (400 – 2600*nm*), are economically affordable and technically easy to setup. Whereas gas discharge tubes (fluorescent tubes or uncoated tubes) are not usable as these tubes emit high narrow lines in the spectrum. Nevertheless, deuterium gas discharge can be used for UV measuring applications and arc sources like xenon lamps can be used for snapshot cameras. LED lamps can be used depending on the implemented technology and use case according to the measuring scenario and emitted wavebands [36]

To acquire a proper datacube different calibration routines are needed to ensure highly accurate reflectance values. Figure 3 shows a generalized processing pipeline for hyperspectral cubes for the demands of plant imaging in greenhouses and laboratories as it is common for plant phenotyping.

Wavelength calibration - from pixel to wavelength

When using a pushbroom sensor one dimension of the detector represents the spatial information of the lines of the target. The other dimension represent the full spectrum of a single line of pixels. The wavelength calibration describes the comparison of measured spectral values with known values [37] and consequently, the mapping of the dispersed geometric axis to wavelength in *nm*.

A calibration is needed after manufacturing and after any

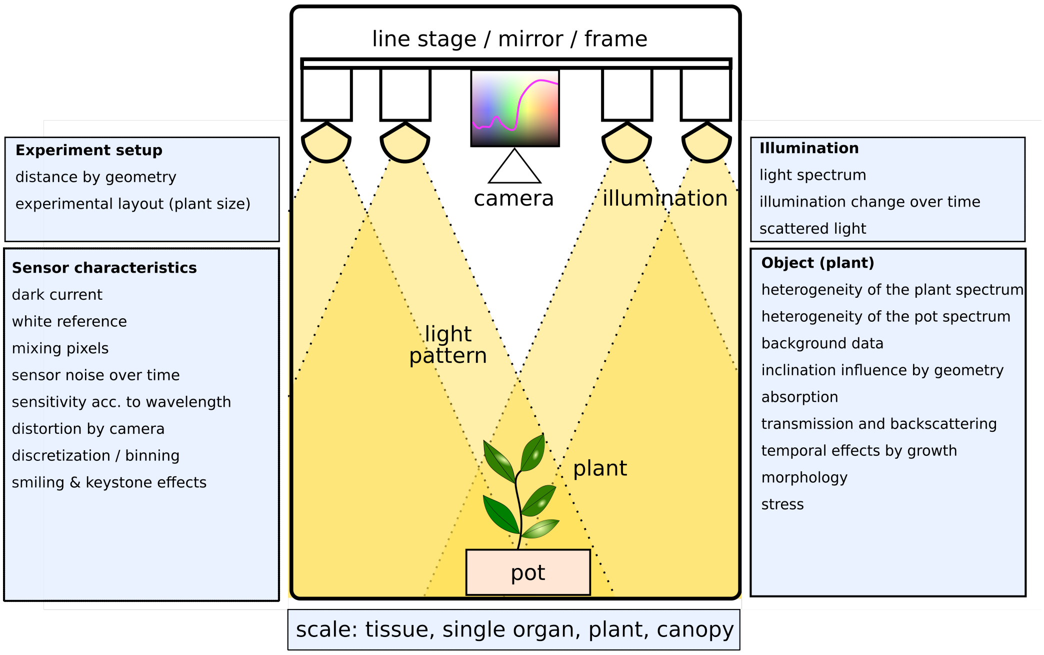

Figure 1. Influences on the measured spectrum of a plant. The four main sources of influence are the experimental setup, the way the camera is mounted, the distance to the plant etc., the light, its spectrum, focus, the object of interest with its absorbing and transmitting properties when imaging plants and the sensor in particular the dark and white referencing, its noise and sensitivity, distortion, discretization and binning.

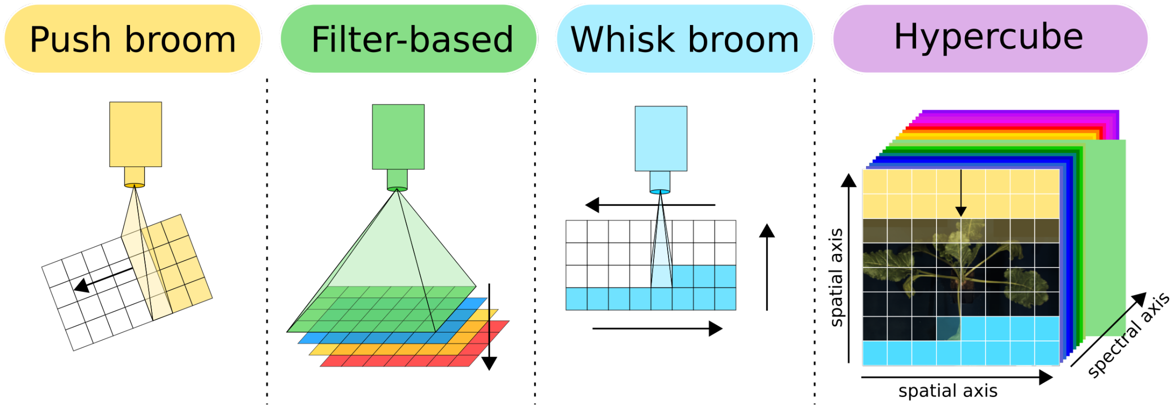

Figure 2. Overview of common HSI techniques: Three different HSI setups are commonly used. Push broom cameras (yellow) are line scanners that were moved over the object or alternatively use a mirror, filter-based systems (green) scan single wavelengths according to the filters one after each other, whisk broom cameras (blue) scan the full spectrum pixel by pixel. All setups result in a 3D hypercube (purple) showing two spatial axis and one spectral axis.

physical changes to the optical path [38]. Wavelength calibration is obtained by exposing the optical system to a calibration light source / sources. Three aspects are critical for obtaining a proper wavelength calibration including (i) the selection of the calibration light, (ii) the determination of the center of characteristic peaks and (iii) a polynomial fitting to the data [39]. The calibration light source / sources should cover the wavelength range to be calibrated. Wavelength calibration light sources emit atomic emission lines of known wavelengths. A polynomial fit of the geometric position of the atomic emission lines on the chip and the known wavelength is conducted. This step is usually performed primarily by the manufacturer and enables displaying the spectral axis in units of wavelength (*nm*).

Instrument function / point spread function - overcoming spectral distortion

Measurements of any optical device can be described as a convolution of the original data with the appropriate transfer function of the sensor and optical setup. This convolution is characterized as a (spectral & spatial) blurring or smearing of the data [40]. The terms "instrument function" and "point spread function" are both used to describe this convolution. The term "point spread function" typically refers to the spatial convolution. The term "instrument function" is referring to the convolution in the spectral domain. Both terms define the highest possible spectral and spatial resolution. Effects resulting from the point spread function are described in the following paragraphs. In contrast to spatial distortions the (spectral) instrument function is typically not corrected.

Spatial calibration - overcoming spatial distortion

Similar to 2D-RGB-cameras which come with barrel and pillow distortion [41], the images of a hyperspectral line scanner tend to show similar effects called smile and keystone effects. Smile is the curvature distortion of the horizontal spectra lines[34] or a shift in wavelength in the spectral domain [42]. Keystone is the distortion of the focal plane rectangle into a trapezoid [34] or a band-to-band mis-registration [42]. These effects can be corrected using geometric control points (GCP) [34]. A spatial calibration of the hyperspectral cube describes the character of

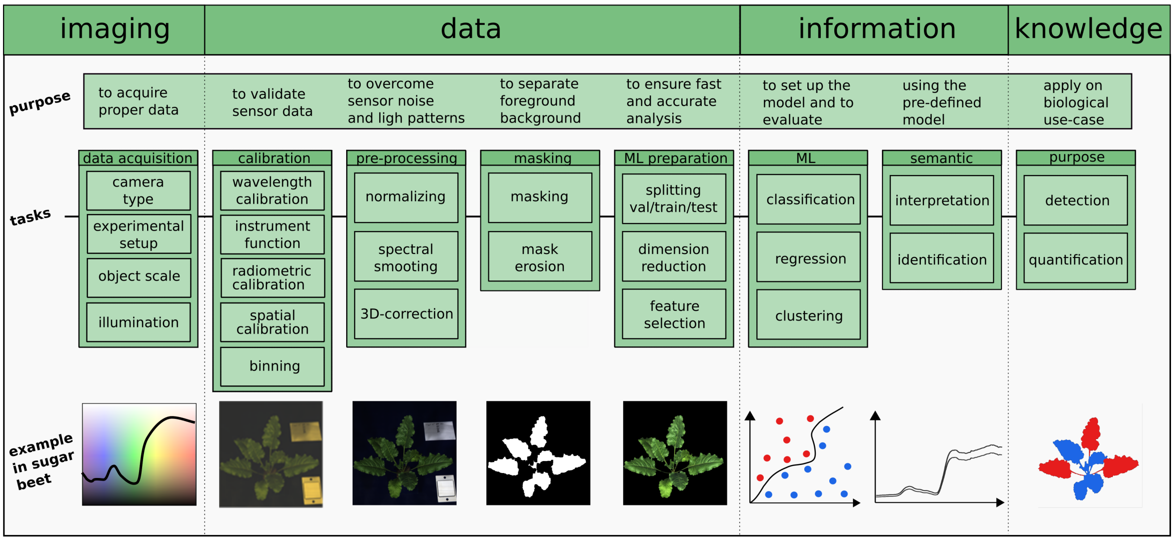

Figure 3. A generalization of a hyperspectral workflow The workflow to extract information from sensor data and to bring it into a biological context to generate knowledge starts with the data acquisition, the hardware calibration, a proper normalization step, data pre-processing, masking to focus on the object of interest, the plant and to cut out background, plant pot and stabilization sticks etc. Depending on the experiment setup data and the analysis type has to be divided into validation, training and test data set to train a model and then to evaluate it on the test data. This is followed by the result interpretation and identification of diseases, stresses or other properties of the plants. Vertical dashed lines describe in a general way the transition between the imaging process, the processing of the data, the generation of information and by interpretation knowledge.

the spatial mapping process. This process results in an rectified image. Not all manufacturers provide this calibration by default.

Radiometric calibration - from counts to a physical unit

Due to differences in quantum efficiency of the detector and varying efficiency of the grating and other optical components (lenses etc.), measurements using different optical systems of the same object under same illumination conditions may not be identical [38]. Data-level is influenced by sensor characteristics, atmospheric conditions, and surface properties of the plants. On the most basic level cameras return their measurement values as digital numbers. To correct for such instrument related variability within these returned digital numbers, radiometric calibration of the measurement device or white referencing is needed. Radiometric calibration transforms these digital numbers to radiance values. Radiance depicts the physical measurement of the spectral power flux emitted, received, transmitted or reflected by an object per unit solid angle and projected area. It uses an integrating sphere to measure the calibration coefficients for each wavelength band (pixel) [43].

The camera digital output is mapped to a physical quantity (radiance) using a certified spectral transfer standard (integrating sphere plus calibrated emitter). Thus, radiometric calibration accounts for the spectral variation of the external lens system, internal optics, sensor and dispersive elements (grating and filter). Radiance values are typically used in high altitude / long distance measurement scenarios (plane or satellite based measurements). Radiometric calibration does not account for a potential active illumination light source, atmospheric absorption between the object under study and the camera system as well as surface properties of the specimen. It corrects for the camera and optics spectrally varying efficiency.

Radiance data can be converted to reflectance data if the irradiation source is known or measured [44] In many applications absolute radiometric calibration and the corresponding radiance data is not required. Often, it is preferred to use reflectance data rather than radiance data. In contrast to radiance data which involves an absolute calibration, reflectance data does not require absolute calibration. A relative spectral calibration to correct for the spectrally varying system efficiency using a simple white reference and dark offset subtraction is sufficient for reflectance measurements. Reflectance data is corrected for camera effects, atmospheric conditions and lighting effects, so only the surface properties of the measured object remain.

Spectral and spatial binning - reducing the noise level

To acquire a high retrieval accuracy within the acquired data a high signal to noise ratio (SNR) is required. SNR is the ratio of the radiance measured to the noise created by the detector and instrument electronics [4]. This ratio can be increased by combining spectral image information along the spectral axis (spectral binning) or by integrating the neighbour pixels (spatial binning) [35]. It was shown that binning along the spectral axis using just a few neighbours reduces the (spectral) image size in favor of an enhanced signal to noise ratio [45]. Nevertheless, lowest SNR ratio is usually found at the beginning and end of the measurable range of a sensor. A common step to deal with this area is simply cutting the first and last few spectral bands of the sensor [36].

In general, wavelengths next to each other are highly correlated [46]. Thus it can be stated, that a limited spectral binning will not affect the informative value of the remaining spectrum.

Binning can be performed directly at the camera internal hardware (hardware binning) or by a processing software when loading the datacube (software binning). In general, hardware binning results in less noise than software binning as the sensor signal is directly merged in the camera prior to analog digital conversion. If using hardware binning, this step has to be performed before any calibration. If using software binning, it is the first step in the pre-processing right after the hardware calibration steps.

Data preprocessing

Pre-processing can be initiated after hardware calibration and measurement validation. A standardized process is needed to compare measurements from different timepoints and from different measuring setups. The pre-procesing steps include the reflectance retrieval, the spectral smoothing and 3D cor-

rection, masking of the object of interest, data splitting, dimension reduction and feature selection for ML.

reflectance retrieval - overcoming the light source influence

To enable comparable measurements for time series within the same measurement setup, between different sensor setups or under different illumination conditions the normalization of the datacube according to the maximum and minimum reflectance intensity is needed. Therefore the dark image is captured by recording the hypercube with a lid on the camera or a closed shutter. This dark data cube described the lowest possible sensor signal. Right after this the white reference spectrum is acquired using a spectrally known reference target. Most often highly reflective materials like barium sulfate (www.SphereOptics.de, www.labsphere.com) act as a reference. Alternatively the use of materials with a known spectral reflectance across the entire spectral range is established as a standard procedure. Here black, dark and light gray objects can be measured with a point spectrometer to get a known reflectance value. When sharing data sets the reference spectral characteristics should be provided as meta-data to ensure the reusability and comfortableness. For performing the normalization step the object scan, the dark currend scan and the reference panel scan are needed. The normalization step can be described by formula 1:

$$I_{Norm} = \frac{cube_O - cube_O^D}{cube_R^W - cube_R^D} \tag{1}$$

Equation 1 has already been described in literature [3] [4]. The numerator describes the subtraction of the measured object cube $cube_O$ and the associated dark current $cube_O^D$, the denominator describes the subtraction of the white reference measurement $cube_R^W$ and the associated dark reference $cube_R^D$. An important feature of Equation 1 is the reduction of non-uniformity caused by either the imaging chip, the illumination or the measuring situation (box etc.).

For measurements in a greenhouse with a variable environment like a change in light condition, or when measuring time series or measurements that cover a large area it is recommended to use multiple targets or periodical re-calibration of the sensor setup.

spectral smoothing - dealing with peaks and spectral outliers

Based on the assumption that the plant spectrum has a smooth spectrum and peaks covering just one or two bands within the spectrum are the result of outliers and noise the use of soft smoothing algorithms is valid. The Savitzky-Golay smoothing algorithm [47] is the most established one for hyperspectral data. [48] showed the applicability for use of 15 centered points and a polynomial of degree 3 for a Specim FX10 camera providing 220 bands within $400 - 1000nm$. Furthermore multiplicative signal correction [49] and standard normal variate [50] are well established routines for signal correction.

3D correction - correcting the influence of the sensor-object distance

The measured reflectance on the detector is depending on the reflected light intensity and the distance between sensor and reflection point on the object/plant. For measuring a plant with upper and lower leaves, the distance to the sensor is different for both leaves. This results in differences in the measured intensity. Some publications show the normalization of the spatial distance [18] [51]. A prerequisite for this is an integration of a 3D measuring device in the measuring setup (laserscanner, ultrasound etc.). Depending on the distance, the corrected cube contains equal reflectance values for similar surfaces although the distance to the camera is different by using pixel wise distance normalization.

segmentation masking

Image segmentation is used to partition an image into meaningful parts that have similar features and properties [52]. For the demands of plant phenotyping this usually means the separation from plant and background pixels. This is mostly based on simple vegetation indices or thresholds using a specific wavelength [53]. Further segmentation like the identification of single leaves, or the detection of disease symptoms are focused on later in the workflow pipeline as ML methods are used to tackle this problem.

After masking, the transition between fore- and background is very sharp. Pixels at this transition include parts of both classes and are depicted as "mixed pixels". To overcome the influence of these pixels to the analysis result, these pixels have to be removed. Literature shows that the use of erosion as a binary image processing technique is efficient. A filter element with the size of $3x3$ pixels is used to shrink the region of the foreground [54]. A negative side effect related to the reduction of foreground data is the possibility of loosing important information which can be used to enhance the data quality.

preparation for ML

Up to this point the datacube consists of hundreds of spectral bands. To detect the specific wavelength that include the biggest impact for the question of interest machine learning is needed. This is also important for a later transfer to multispectral cameras with less spectral bands but with the opportunity to measure in high throughput on the field scale.

To prepare the data for use in a common ML routine, using supervised classification approaches, the data set is split into three subgroups including the same distribution of groups within the three sets. That means the ratio between the included groups is similar. Set one is called the training set and is used to calculate the model of the ML method like support vector machines (SVM) or decision trees (DT). Set two is called validation data set and is used for model hyper-parameter tuning. The third set is called the test set and is used to evaluate the performance of the developed model and to calculate a model accuracy. The size of the groups differs with respect to the number available samples. A repeated cross-validation using different splits of the data set (test and training) is recommended. Dimensionality reduction methods can decrease spectral redundancy and reduce data volume within the data set. Common techniques are principal component analysis (PCA) [55], feature selection using recursive feature elimination (RFE) [56], ReliefF [57] or correlation-based feature selection [58].

Data analysis and interpretation

hyperspectral traits

Hyperspectral traits can be categorized into different groups, depending of the focus of the data. If the data is coming from a single plant (trait level 1) the datacube can be used to derive very lowly resolved information about the plant such as the plant canopy [59]. If the datacube is segmented into regions including single leaves, disease symptoms or spatially confined areas (ROI, trait level 2), these regions can be compared together. This is commonly done by a classification on pixel (single spectrum) level [60]. Time series measurements are essential for accurate capturing of developing disease symptoms. This leads to the development of hyperspectral dynamics over time (trait level 3) [24] [48]. Hyperspectral datacubes are affected by distance and inclination of the measured object.

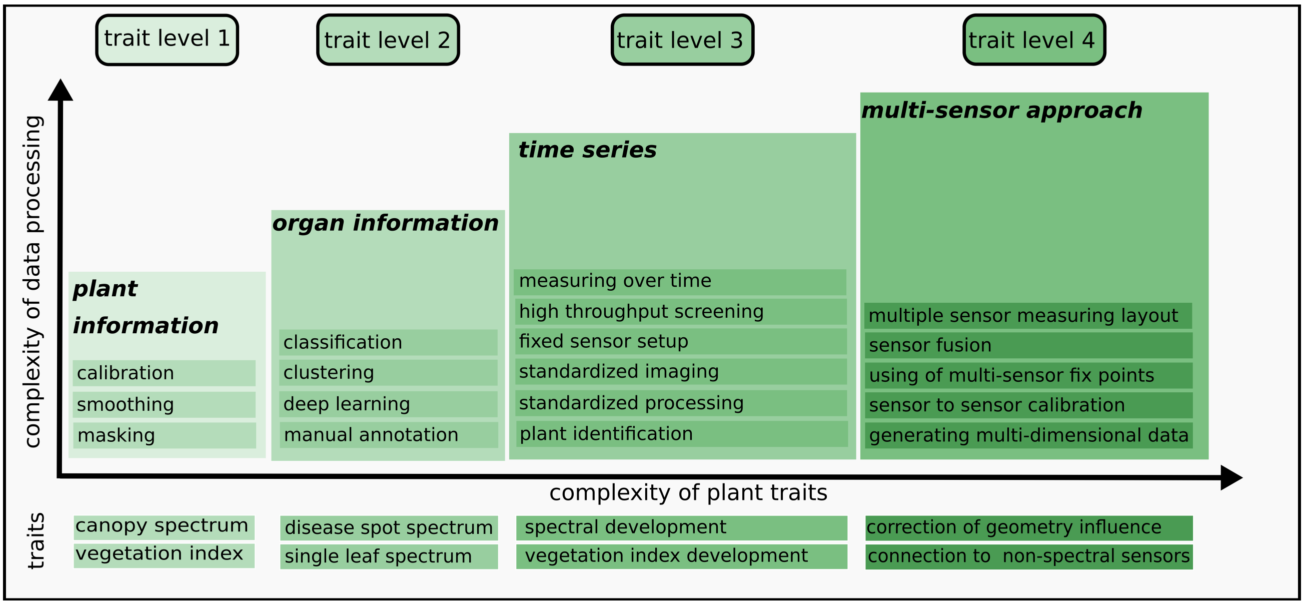

Figure 4. A general trait visualization Plant traits are parameters that describe the hyperspectral properties of the plant tissue. Nevertheless, these traits can be grouped according to the effort that is needed for their extraction. First level (1) traits describe the spectrum of the whole canopy. By using a classification based on ML algorithm it is possible to identify spectra of single leaves (level 2). By measurements over time the development of these spectra can be visualized (level 3) and by using further sensors it is possible to reduce geometrical effects based on a sensor fusion (level 4).

The correction of the hyperspectral information according to distance and inclination is needed. This can be done by modeling the measuring setup and the occurring errors. It needs the use of an accompanying sensor, measuring the object geometry such as a 3D laserscanner [61] [62] and fusing the data for a complete 3D-hyperspectral data model that enables detection of plant disease within a corrected spectrum [33]. An overview about these traits, prerequisites and applications is shown in Fig. 4.

machine learning

For data analysis and ML, the tasks can be divided into supervised methods and unsupervised methods. Supervised methods require a known target value and therefore labeled data to train a model. Within the supervised learning methods, methods can be grouped by their target. If the output is a label as an affiliation to a group and thus is categorical, the method is called classification. Prominent routines for supervised classification are SVM, DT and Neural Network architectures (NN). A similar approach using labeled data is regression, where the output does not predict a group but a numeric value. Known methods for this scenario are Support Vector Regression (SVR), DT and NN.

A special case of ML is Deep Learning (DL). DL allows computational models that are composed of multiple processing layers to learn representations of data with multiple levels of abstraction. It also describes an algorithm allowing raw data as input and automatically discovers a representation, consisting of multiple non-linear modules, for detection or classification [63]. In contrast to SVM or DT approaches, DL is based on NN architectures and depends on huge labelled datasets for training. DL approaches have been widely used on RGB images for the demands of plant phenotyping as a classification of root tips, shoot and leaves [64] [65] and can be depicted to be state of the art. During the last years, hyperspectral applications are raising. Different types of DL approaches have been used for plant disease [66] or stress detection [67].

Usually the results of a classification are presented by a confusion matrix, which indicates for a specific trained model the resulting classification of the test dataset regarding true positive, false positives, false negative and true negative. It compares the predicted values to the true values.

Unsupervised approaches do not need labeled data and try to detect patterns within the data. Clustering approaches like k-means shift manual work from model generation to cluster interpretation as it is the task of the scientist to give semantic to the clustered datasets. The clustering of hyperspectral datasets has been successfully shown for the detection of drought for maize [68].

Challenges and limitations

HSI has to face many challenges regarding sensor setup, illumination, data processing and plant specific characteristics. Starting with the measuring setup where the sensor, illumination and the object distance has to be adapted to the plant size to gain best reflectance results. Thus, the setup has to tailored has to be tailored towards the size of the plants. Both extrema within one measuring setup causes problems in illumination, image resolution and chip intensity.

When extending hyperspectral imaging to the UV area between 200–400nm, plants can suffer from the harmful properties of illumination in this spectral region [5]. Further evaluation of the effects of light exposure on the study objects is recommended as plant properties such as architecture, tissue composure and wax layer differ between species.

Surface geometry has a remarkable effect on the measured spectrum. [7] found a correlation between normalized difference vegetation index (NDVI) and surface inclination. Thus this effect has to be taken into account or if possible has to be corrected. This emphasizes the need for imaging setups including different sensors for geometry and reflectance.

The workflow proposed is not transferable to field conditions which requires a different experimental set up to ensure high quality hyperspectral measurements [69].

High throughput imaging setups [21] combine hyperspectral cameras with high frequent imaging which leads to complex datasets independent of the scale [70]. This emphasized the need for reliable, stable and efficient algorithms and high-end computational machines to process the datacubes. Image

analysis and interpretation is the key plant phenotyping bottleneck [71].

Conclusion

HSI is a well-established tool for plant phenotyping in greenhouses. But each laboratory is using a specialized workflow for data assessing, processing and handling which makes the data individually valid but difficult to compare.

This study introduces a generalized workflow for handling hyperspectral image data for greenhouses and laboratories. It includes calibration, reflectance retrieval, data smoothing, masking and preparation for use in a machine learning routine.

This workflow includes hardware-based calibration steps as well as software based processing. Furthermore, a general definition for hyperspectral traits is introduced to establish a level-system starting from traits for the whole plant, to traits for single organs, traits describing temporal development and traits that are based on the measurements of different sensors. An literature overview using hyperspectral imaging and ML is introduced to show the different application areas for plant measuring in agriculture together with the used ML method and the used plant material. Thus a general overview for the application of hyperspectral imaging in plant science is reasonable. This review offers a standardized protocol for raw data processing and how plant traits can be categorized due to their complexity regarding effort in data processing and derivable traits.

Declarations

List of abbreviations

- (A)NN – (artificial) neural network
- CNN – convolutional neural network
- DC – dark current
- DT – decision tree
- (F)LDA – (fishers) linear discriminant analysis
- FNN – fully connected neural network
- GAN – generative adversarial network
- HSI – hyperspectral imaging
- ML – machine learning
- NDVI – normalized difference vegetation index
- NIR – near infrared (700 – 1000nm)
- PLS (R) – partial least square (regression)
- QTL – Quantitative Trait Locus
- RGB – red, green, blue, digital camera sensor
- SAE – stacked auto encoder
- SAM – spectral angle mapper
- SDA – stepwise discriminant analysis
- SNR – signal noise ratio
- SiVm – simplex volume maximization
- SVDD – support vector data descriptor
- SVM – support vector machines
- SWIR – short wave infrared (1000 – 2500nm)
- UV – ultra violett spectrum (< 380nm)
- VIS – visual spectrum (380 – 700nm)
- VNIR – visual + infrared spectrum (380 – 1000nm)
- WR – white reference

Ethical Approval (optional)

“Not applicable”

Consent for publication

“Not applicable”

Competing Interests

‘The authors declare that they have no competing interests’.

Funding

This study was partially funded by the Deutsche Forschungsgemeinschaft (DFG, German Research Foundation) under Germany's Excellence Strategy - EXC 2070 - 390732324. Furthermore it was supported by Bayer AG - Crop Science.

Author’s Contributions

SP and AKM designed the research. AKM supervised the project. SP and AKM designed the review manuscript, prepared the figures and studied the literature. All authors read and approved the final version of the article.

Acknowledgements

We would like to thank Oliver Lischtschenko from Ocean Optics B.V. for his helpful comments and suggestions regarding the hyperspectral sensor calibration. Furthermore we would like to thank Patrick Schramowski for proofreading the machine learning part and Abel Barreto and Anita Kuepper for proofreading and help with the figures.

Authors’ information (optional)

References

1. Mahlein AK, Kuska MT, Thomas S, Wahabzada M, Behmann J, Rascher U, et al. Quantitative and qualitative phenotyping of disease resistance of crops by hyperspectral sensors: seamless interlocking of phytopathology, sensors, and machine learning is needed! Current Opinion in Plant Biology 2019 Aug;50:156–162. https://doi.org/10.1016/j.pbi.2019.06.007.
2. Fiorani F, Rascher U, Jahnke S, Schurr U. Imaging plants dynamics in heterogenic environments. Current Opinion in Biotechnology 2012 Apr;23(2):227–235. https://doi.org/10.1016/j.copbio.2011.12.010.
3. Elmasry G, Kamruzzaman M, Sun DW, Allen P. Principles and Applications of Hyperspectral Imaging in Quality Evaluation of Agro-Food Products: A Review. Critical Reviews in Food Science and Nutrition 2012 Nov;52(11):999–1023. https://doi.org/10.1080/10408398.2010.543495.
4. Elmasry G, Sun DW. Principles of Hyperspectral Imaging Technology. In: Hyperspectral Imaging for Food Quality Analysis and Control Elsevier; 2010.p. 3–43. https://doi.org/10.1016/b978-0-12-374753-2.10001-2.
5. Brugger A, Behmann J, Paulus S, Luigs HG, Kuska MT, Schramowski P, et al. Extending hyperspectral imaging for plant phenotyping to the UV-range. Remote Sensing 2019 Jun;11(12):1401. https://doi.org/10.3390/rs11121401.
6. Behmann J, Acebron K, Emin D, Bennertz S, Matsubara S, Thomas S, et al. Specim IQ: Evaluation of a new, miniaturized handheld hyperspectral camera and its application for plant phenotyping and disease detection. Sensors 2018 Feb;18(2):441. https://doi.org/10.3390/s18020441.

7. Behmann J, Mahlein AK, Paulus S, Dupuis J, Kuhlmann H, Oerke EC, et al. Generation and application of hyperspectral 3D plant models: methods and challenges. Machine Vision and Applications 2015 Oct;27(5):611–624. https://doi.org/10.1007/s00138-015-0716-8.
8. Croft H, Chen JM. Leaf Pigment Content. In: Comprehensive Remote Sensing Elsevier; 2018.p. 117–142. https://doi.org/10.1016/b978-0-12-409548-9.10547-0.
9. Corti M, Gallina PM, Cavalli D, Cabassi G. Hyperspectral imaging of spinach canopy under combined water and nitrogen stress to estimate biomass, water, and nitrogen content. Biosystems Engineering 2017 Jun;158:38–50. https://doi.org/10.1016/j.biosystemseng.2017.03.006.
10. Behmann J, Steinrücken J, Plümer L. Detection of early plant stress responses in hyperspectral images. ISPRS Journal of Photogrammetry and Remote Sensing 2014 Jul;93:98–111. https://doi.org/10.1016/j.isprsjprs.2014.03.016.
11. Mahlein AK, Kuska MT, Behmann J, Polder G, Walter A. Hyperspectral Sensors and Imaging Technologies in Phytopathology: State of the Art. Annual Review of Phytopathology 2018 Aug;56(1):535–558. https://doi.org/10.1146/annurev-phyto-080417-050100.
12. Pierna JAF, Vermeulen P, Amand O, Tossens A, Dardenne P, Baeten V. NIR hyperspectral imaging spectroscopy and chemometrics for the detection of undesirable substances in food and feed. Chemometrics and Intelligent Laboratory Systems 2012 Aug;117:233–239. https://doi.org/10.1016/j.chemolab.2012.02.004.
13. Huang M, Wan X, Zhang M, Zhu Q. Detection of insect-damaged vegetable soybeans using hyperspectral transmittance image. Journal of Food Engineering 2013 May;116(1):45–49. https://doi.org/10.1016/j.jfoodeng.2012.11.014.
14. Yang W, Yang C, Hao Z, Xie C, Li M. Diagnosis of Plant Cold Damage Based on Hyperspectral Imaging and Convolutional Neural Network. IEEE Access 2019;7:118239–118248. https://doi.org/10.1109/access.2019.2936892.
15. Zhang SY, Fei T, Ran YH. Diagnosis of heavy metal cross contamination in leaf of rice based on hyperspectral image: a greenhouse experiment. In: 2018 IEEE International Conference on Advanced Manufacturing (ICAM) IEEE; 2018. p. 159–162. https://doi.org/10.1109/amcon.2018.8614938.
16. Nansen C, Zhao G, Dakin N, Zhao C, Turner SR. Using hyperspectral imaging to determine germination of native Australian plant seeds. Journal of Photochemistry and Photobiology B: Biology 2015 Apr;145:19–24. https://doi.org/10.1016/j.jphotobiol.2015.02.015.
17. Moghadam P, Ward D, Goan E, Jayawardena S, Sikka P, Hernandez E. Plant disease detection using hyperspectral imaging. In: 2017 International Conference on Digital Image Computing: Techniques and Applications (DICTA) IEEE; 2017. p. 1–8. https://doi.org/10.1109/dicta.2017.8227476.
18. Reddy KN, Huang Y, Lee MA, Nandula VK, Fletcher RS, Thomson SJ, et al. Glyphosate-resistant and glyphosate-susceptible palmer amaranth (*amaranthus palmeriS. Wats.*): hyperspectral reflectance properties of plants and potential for classification. Pest Management Science 2014 Mar;70(12):1910–1917. https://doi.org/10.1002/ps.3755.
19. Zhang C, Ye H, Liu F, He Y, Kong W, Sheng K. Determination and Visualization of pH Values in Anaerobic Digestion of Water Hyacinth and Rice Straw Mixtures Using Hyperspectral Imaging with Wavelet Transform Denoising and Variable Selection. Sensors 2016 Feb;16(2):244. https://doi.org/10.3390/s16020244.
20. Yu X, Lu H, Liu Q. Deep-learning-based regression model and hyperspectral imaging for rapid detection of nitrogen concentration in oilseed rape (Brassica napus L.) leaf. Chemometrics and Intelligent Laboratory Systems 2018 Jan;172:188–193. https://doi.org/10.1016/j.chemolab.2017.12.010.
21. Ge Y, Bai G, Stoerger V, Schnable JC. Temporal dynamics of maize plant growth, water use, and leaf water content using automated high throughput RGB and hyperspectral imaging. Computers and Electronics in Agriculture 2016 Sep;127:625–632. https://doi.org/10.1016/j.compag.2016.07.028.
22. Rumpf T, Mahlein AK, Steiner U, Oerke EC, Dehne HW, Plümer L. Early detection and classification of plant diseases with Support Vector Machines based on hyperspectral reflectance. Computers and Electronics in Agriculture 2010 Oct;74(1):91–99. https://doi.org/10.1016/j.compag.2010.06.009.
23. Leucker M, Mahlein AK, Steiner U, Oerke EC. Improvement of lesion phenotyping in *cercospora beticola*–sugar beet interaction by hyperspectral imaging. Phytopathology 2016 Feb;106(2):177–184. https://doi.org/10.1094/phyto-04-15-0100-r.
24. Behmann J, Bohnenkamp D, Paulus S, Mahlein AK. Spatial referencing of hyperspectral images for tracing of plant disease symptoms. Journal of Imaging 2018 Dec;4(12):143. https://doi.org/10.3390/jimaging4120143.
25. Nansen C, Sidumo AJ, Capareda S. Variogram analysis of hyperspectral data to characterize the impact of biotic and abiotic stress of maize plants and to estimate biofuel potential. Applied Spectroscopy 2010 Jun;64(6):627–636. https://doi.org/10.1366/000370210791414272.
26. Elvanidi A, Katsoulas N, Ferentinos KP, Bartzanas T, Kittas C. Hyperspectral machine vision as a tool for water stress severity assessment in soilless tomato crop. Biosystems Engineering 2018 Jan;165:25–35. https://doi.org/10.1016/j.biosystemseng.2017.11.002.
27. Moghimi A, Yang C, Miller ME, Kianian S, Marchetto P. Hyperspectral imaging to identify salt-tolerant wheat lines. In: Thomasson JA, McKee M, Moorhead RJ, editors. Autonomous Air and Ground Sensing Systems for Agricultural Optimization and Phenotyping II SPIE; 2017. p. 1182. https://doi.org/10.1117/12.2262388.
28. Pandey P, Ge Y, Stoerger V, Schnable JC. High throughput in vivo analysis of plant leaf chemical properties using hyperspectral imaging. Frontiers in Plant Science 2017 Aug;8. https://doi.org/10.3389/fpls.2017.01348.
29. do Prado Ribeiro L, Klock ALS, Filho JAW, Tramontin MA, Trapp MA, Mithöfer A, et al. Hyperspectral imaging to characterize plant-plant communication in response to insect herbivory. Plant Methods 2018 Jul;14(1). https://doi.org/10.1186/s13007-018-0322-7.
30. Forster A, Behley J, Behmann J, Roscher R. Hyperspectral Plant Disease Forecasting Using Generative Adversarial Networks. In: IGARSS 2019 - 2019 IEEE International Geoscience and Remote Sensing Symposium IEEE; 2019. p. 1793–1796. https://doi.org/10.1109/igarss.2019.8898749.
31. Barreto A, Paulus S, Varrelmann M, Mahlein AK. Hyperspectral imaging of symptoms induced by Rhizoctonia solani in sugar beet: comparison of input data and different machine learning algorithms. Journal of Plant Diseases and Protection 2020;.
32. Berdugo CA, Zito R, Paulus S, Mahlein AK. Fusion of sensor data for the detection and differentiation of plant diseases in cucumber. Plant Pathology 2014 May;63(6):1344–1356. https://doi.org/10.1111/ppa.12219.
33. Roscher R, Behmann J, Mahlein AK, Dupuis J, Kuhlmann H, Plümer L. Detection of disease symptoms on hyperspectral 3D plant models. ISPRS Annals of Photogrammetry, Remote Sensing and Spatial Information

Sciences 2016 Jun;III-7:89–96. https://doi.org/10.5194/isprs-annals-iii-7-89-2016.
34. Lawrence KC, Park B, Windham WR, Mao C. Calibration of a pushbroom hyperspectral imaging system for agricultural inspection. Transactions of the ASAE 2003;46(2). https://doi.org/10.13031/2013.12940.
35. Rehman SU, Kumar A, Banerjee A. SNR improvement for hyperspectral application using frame and pixel binning. In: Xiong XJ, Kuriakose SA, Kimura T, editors. Earth Observing Missions and Sensors: Development, Implementation, and Characterization IV SPIE; 2016. p. 134 – 139. https://doi.org/10.1117/12.2220599.
36. Mahlein AK, Hammersley S, Oerke EC, Dehne HW, Goldbach H, Grieve B. Supplemental Blue LED Lighting Array to Improve the Signal Quality in Hyperspectral Imaging of Plants. Sensors 2015 Jun;15(6):12834–12840. https://doi.org/10.3390/s150612834.
37. Gaigalas AK, Wang L, He HJ, DeRose P. Procedures for Wavelength Calibration and Spectral Response Correction of CCD Array Spectrometers. Journal of Research of the National Institute of Standards and Technology 2009 Jul;114(4):215. https://doi.org/10.6028/jres.114.015.
38. Yao H, Lewis D. Spectral Preprocessing and Calibration Techniques. In: Hyperspectral Imaging for Food Quality Analysis and Control Elsevier; 2010.p. 45–78. https://doi.org/10.1016/b978-0-12-374753-2.10002-4.
39. Sun YC, Huang C, Xia G, Jin SQ, Lu HB. Accurate wavelength calibration method for compact CCD spectrometer. Journal of the Optical Society of America A 2017 Mar;34(4):498. https://doi.org/10.1364/josaa.34.000498.
40. Kumar VN, Rao DN. Determination of the instrument function of a grating spectrometer by using white-light interferometry. Applied Optics 1997 Jul;36(19):4535. https://doi.org/10.1364/ao.36.004535.
41. Weng J, Cohen P, Herniou M. Camera calibration with distortion models and accuracy evaluation. IEEE Transactions on Pattern Analysis and Machine Intelligence 1992;14(10):965–980. https://doi.org/10.1109/34.159901.
42. Yokoya N, Miyamura N, Iwasaki A. Preprocessing of hyperspectral imagery with consideration of smile and keystone properties. In: Larar AM, Chung HS, Suzuki M, editors. Multispectral, Hyperspectral, and Ultraspectral Remote Sensing Technology, Techniques, and Applications III SPIE; 2010. p. 73 – 81. https://doi.org/10.1117/12.870437.
43. Hruska R, Mitchell J, Anderson M, Glenn NF. Radiometric and Geometric Analysis of Hyperspectral Imagery Acquired from an Unmanned Aerial Vehicle. Remote Sensing 2012 Sep;4(9):2736–2752. https://doi.org/10.3390/rs4092736.
44. Durell C. White paper: Top-of-Atmosphere reflectance calibration of satellite and airborne sensor systems using FLARE vicarious calibration network. North Sutton, NH 03260 USA: Labsphere, Inc; 2016.
45. Polder G, van der Heijden GW. Calibration and characterization of spectral imaging systems. In: Tong Q, Zhu Y, Zhu Z, editors. Multispectral and Hyperspectral Image Acquisition and Processing SPIE; 2001. p. 10 – 17. https://doi.org/10.1117/12.441362.
46. Mahlein AK, Rumpf T, Welke P, Dehne HW, Plümer L, Steiner U, et al. Development of spectral indices for detecting and identifying plant diseases. Remote Sensing of Environment 2013 Jan;128:21–30. https://doi.org/10.1016/j.rse.2012.09.019.
47. Savitzky A, Golay MJE. Smoothing and differentiation of data by simplified least squares procedures. Analytical Chemistry 1964 Jul;36(8):1627–1639. https://doi.org/10.1021/ac60214a047.
48. Bohnenkamp D, Kuska MT, Mahlein AK, Behmann J. Hyperspectral signal decomposition and symptom detection of wheat rust disease at the leaf scale using pure fungal spore spectra as reference. Plant Pathology 2019 Apr;68(6):1188–1195. https://doi.org/10.1111/ppa.13020.
49. Bodner G, Nakhforoosh A, Arnold T, Leitner D. Hyperspectral imaging: a novel approach for plant root phenotyping. Plant Methods 2018 Oct;14(1). https://doi.org/10.1186/s13007-018-0352-1.
50. Asaari MSM, Mishra P, Mertens S, Dhondt S, Inzé D, Wuyts N, et al. Close-range hyperspectral image analysis for the early detection of stress responses in individual plants in a high-throughput phenotyping platform. ISPRS Journal of Photogrammetry and Remote Sensing 2018 Apr;138:121–138. https://doi.org/10.1016/j.isprsjprs.2018.02.003.
51. Huang Y, Lee MA, Nandula VK, Reddy KN. Hyperspectral Imaging for Differentiating Glyphosate-Resistant and Glyphosate-Susceptible Italian Ryegrass. American Journal of Plant Sciences 2018;09(07):1467–1477. https://doi.org/10.4236/ajps.2018.97107.
52. Kaur D, Kaur Y. Various image segmentation techniques: a review. International Journal of Computer Science and Mobile Computing 2014;3(5):809–814.
53. Hillnhütter C, Mahlein AK, Sikora RA, Oerke EC. Use of imaging spectroscopy to discriminate symptoms caused by Heterodera schachtii and Rhizoctonia solani on sugar beet. Precision Agriculture 2011 Jun;13(1):17–32. https://doi.org/10.1007/s11119-011-9237-2.
54. Moghimi A, Yang C, Miller ME, Kianian SF, Marchetto PM. A Novel Approach to Assess Salt Stress Tolerance in Wheat Using Hyperspectral Imaging. Frontiers in Plant Science 2018 Aug;9. https://doi.org/10.3389/fpls.2018.01182.
55. Williams PJ, Kucheryavskiy S. Classification of maize kernels using NIR hyperspectral imaging. Food Chemistry 2016 Oct;209:131–138. https://doi.org/10.1016/j.foodchem.2016.04.044.
56. Guyon I, Weston J, Barnhill S, Vapnik V. Gene Selection for Cancer Classification using Support Vector Machines. Machine Learning 2002;46(1/3):389–422. https://doi.org/10.1023/a:1012487302797.
57. Robnik-Šikonja M, Kononenko I. Theoretical and Empirical Analysis of ReliefF and RReliefF. Machine Learning 2003;53(1/2):23–69. https://doi.org/10.1023/a:1025667309714.
58. Moghimi A, Yang C, Marchetto PM. Ensemble feature selection for plant phenotyping: a journey from hyperspectral to multispectral imaging. IEEE Access 2018;6:56870–56884. https://doi.org/10.1109/access.2018.2872801.
59. Cao X, Luo Y, Zhou Y, Duan X, Cheng D. Detection of powdery mildew in two winter wheat cultivars using canopy hyperspectral reflectance. Crop Protection 2013 Mar;45:124–131. https://doi.org/10.1016/j.cropro.2012.12.002.
60. Bergsträsser S, Fanourakis D, Schmittgen S, Cendrero-Mateo M, Jansen M, Scharr H, et al. HyperART: non-invasive quantification of leaf traits using hyperspectral absorption-reflectance-transmittance imaging. Plant Methods 2015;11(1):1. https://doi.org/10.1186/s13007-015-0043-0.
61. Behmann J, Mahlein AK, Paulus S, Kuhlmann H, Oerke EC, Plümer L. Calibration of hyperspectral close-range pushbroom cameras for plant phenotyping. ISPRS Journal of Photogrammetry and Remote Sensing 2015 Aug;106:172–182. https://doi.org/10.1016/j.isprsjprs.2015.05.010.
62. Paulus S. Measuring crops in 3D: using geometry for plant phenotyping. Plant Methods 2019 Sep;15(1). https://doi.org/10.1186/s13007-019-0490-0.
63. LeCun Y, Bengio Y, Hinton G. Deep learning. Nature 2015 May;521(7553):436–444. https://doi.org/10.1038/nature14539.

64. Pound MP, Atkinson JA, Townsend AJ, Wilson MH, Griffiths M, Jackson AS, et al. Deep machine learning provides state-of-the-art performance in image-based plant phenotyping. GigaScience 2017 Aug;6(10). https://doi.org/10.1093/gigascience/gix083.
65. Mochida K, Koda S, Inoue K, Hirayama T, Tanaka S, Nishii R, et al. Computer vision-based phenotyping for improvement of plant productivity: a machine learning perspective. GigaScience 2018 Dec;8(1). https://doi.org/10.1093/gigascience/giy153.
66. Golhani K, Balasundram SK, Vadamalai G, Pradhan B. A review of neural networks in plant disease detection using hyperspectral data. Information Processing in Agriculture 2018 Sep;5(3):354–371. https://doi.org/10.1016/j.inpa.2018.05.002.
67. Singh AK, Ganapathysubramanian B, Sarkar S, Singh A. Deep Learning for Plant Stress Phenotyping: Trends and Future Perspectives. Trends in Plant Science 2018 Oct;23(10):883–898. https://doi.org/10.1016/j.tplants.2018.07.004.
68. Asaari MSM, Mertens S, Dhondt S, Wuyts N, Scheunders P. Detection of plant responses to drought using close-range hyperspectral imaging in a high-throughput phenotyping platform. In: 2018 9th Workshop on Hyperspectral Image and Signal Processing: Evolution in Remote Sensing (WHISPERS) IEEE; 2018. p. 121 – 138. https://doi.org/10.1109/whispers.2018.8747228.
69. Bohnenkamp D, Behmann J, Mahlein AK. In-Field Detection of Yellow Rust in Wheat on the Ground Canopy and UAV Scale. Remote Sensing 2019 Oct;11(21):2495. https://doi.org/10.3390/rs11212495.
70. Virlet N, Sabermanesh K, Sadeghi-Tehran P, Hawkesford MJ. Field Scanalyzer: An automated robotic field phenotyping platform for detailed crop monitoring. Functional Plant Biology 2017;44(1):143. https://doi.org/10.1071/fp16163.
71. Campbell ZC, Acosta-Gamboa LM, Nepal N, Lorence A. Engineering plants for tomorrow: how high-throughput phenotyping is contributing to the development of better crops. Phytochemistry Reviews 2018 Jul;17(6):1329–1343. https://doi.org/10.1007/s11101-018-9585-x.

Personal Cover Click here to access/download;Personal Cover;CoveringLetter.docx

Dr. Stefan Paulus

Institute of Sugar Beet Research

Holtenser Landstr. 77

37079 Göttingen

Dear Sir or Madam,

Please find attached our revised manuscript “Technical workflows for hyperspectral plant image assessment and processing on the greenhouse and laboratory scale“ for submission in GigaScience.

The comments of the reviewers have been very helpful and have been completely included into the manuscript. Furthermore the language has been checked by a native speaker.

We hope that the study now is acceptable for publication in your journal.

Kind regards,

Stefan Paulus
