# Supplementary material for: Technical workflows for hyperspectral plant image assessment and processing on the greenhouse and laboratory scale
Source: Gigascience. 2020 Aug 20;9(8):giaa090. doi: 10.1093/gigascience/giaa090 (PMC7439585; doi:10.1093/gigascience/giaa090)
Supplement: giaa090_Response_to_Reviewer_Comments_Revision_1 [file giaa090_response_to_reviewer_comments_revision_1.pdf]

Author's Response To Reviewer Comments

Close

The comments of the reviewers have been very helpful and have been completely included into the manuscript. Furthermore the language has been checked by a native speaker.

Close
