# Supplementary material for: Technical workflows for hyperspectral plant image assessment and processing on the greenhouse and laboratory scale
Source: Gigascience. 2020 Aug 20;9(8):giaa090. doi: 10.1093/gigascience/giaa090 (PMC7439585; doi:10.1093/gigascience/giaa090)
Supplement: giaa090_Reviewer_1_Report_Original_Submission — Yu Jiang -- 4/9/2020 Reviewed [file giaa090_reviewer_1_report_original_submission.pdf]

Reviewer Report

Title: Technical workflows for hyperspectral plant image assessment and processing on the greenhouse and laboratory scale

Version: Original Submission **Date:** 4/9/2020

Reviewer name: Yu Jiang

Reviewer Comments to Author:

The submitted manuscript reviewed relevant literature and summarized a general workflow for the analysis of plant hyperspectral images collected in controlled environments. This review could have a great impact to the research community: The general workflow could guide researchers to standardize the data acquisition and processing of plant hyperspectral images for controlled environment studies, help accumulate global research efforts, promote the data sharing, and ultimately advance big data analysis for plant spectral responses and therefore biological understanding. Therefore, the manuscript fits well with the journal's scope and could be of great interest to readers. There are some parts need to be further improved or explained.

1. In my opinion, a unique feature of spectral imaging is the combination of spatial and spectral information for objects rather than the combination of spatial and temporal information, which has been stated by the authors in the first paragraph in Background section.
2. Details and explanations are needed for the data acquisition section. While line-scan (pushbroom) systems are widely used, many researchers also used area scanning mode (rarely point scanning, aka whiskbroom, mode) for studying plant spectral responses. To the best comprehensiveness, it would be better to briefly introduce all three scanning modes including basic system setup and pros and cons of using each mode. A figure may be added for the best illustration of the system setups.
3. Data pre-processing (e.g., reflectance calibration or flat field correction)/meta-data information is utmost important for sharing plant hyperspectral images. Authors may consider to emphasize this importance and provide more information on how to select reference targets. For example, Spectralon targets are generally in good quality with known spectral characteristics, so data collected using this type of reference targets could be directly shared as long as the target model number and manufacturer are provided. In case Spectralon targets cannot be used (due to either cost consideration or spatial limitation), inexpensive alternative references can be used but the reference spectral characteristics should be provided as meta-data to ensure the reusability and comparableness of shared datasets.
4. Authors may consider use "flat field correction" as the name for the section of "reflectance calibration /normalizing …". An important feature of applying Eq.1. to images is to reduce nonuniformity caused by either the imaging chip, illumination, or both.
5. In the section of "preparation for ML", please consider adjusting the description order as "training", "validation", and "testing", which is logically natural and widely used by research communities. Authors may also consider cite a technical-driven review paper on feature selection. This will help readers to further the understanding and knowledge of the techniques can be potentially used.
6. It would be very interesting and useful if authors could provide a table to list some publicly

available datasets that were collected by following the general workflow. This will in turn help the technical community to obtain domain datasets for the development of new tools in the future.

7. There are some repeated words and typos to be carefully checked by the authors. For example: "publications" in the abstract and "bedefined" to "be defined" in the Data acquisition and processing section.

Methods

Are the methods appropriate to the aims of the study, are they well described, and are necessary controls included? Choose an item.

Conclusions

Are the conclusions adequately supported by the data shown? Choose an item.

Reporting Standards

Does the manuscript adhere to the journal's guidelines on minimum standards of reporting? Choose an item.

Choose an item.

Statistics

Are you able to assess all statistics in the manuscript, including the appropriateness of statistical tests used? Choose an item.

Quality of Written English

Please indicate the quality of language in the manuscript: Choose an item.

Declaration of Competing Interests

Please complete a declaration of competing interests, considering the following questions:

- Have you in the past five years received reimbursements, fees, funding, or salary from an organisation that may in any way gain or lose financially from the publication of this manuscript, either now or in the future?
- Do you hold any stocks or shares in an organisation that may in any way gain or lose financially from the publication of this manuscript, either now or in the future?
- Do you hold or are you currently applying for any patents relating to the content of the manuscript?
- Have you received reimbursements, fees, funding, or salary from an organization that holds or has applied for patents relating to the content of the manuscript?

- Do you have any other financial competing interests?
- Do you have any non-financial competing interests in relation to this paper?

If you can answer no to all of the above, write 'I declare that I have no competing interests' below. If your reply is yes to any, please give details below.

I declare that I have no competing interests

I agree to the open peer review policy of the journal. I understand that my name will be included on my report to the authors and, if the manuscript is accepted for publication, my named report including any attachments I upload will be posted on the website along with the authors' responses. I agree for my report to be made available under an Open Access Creative Commons CC-BY license (http://creativecommons.org/licenses/by/4.0/). I understand that any comments which I do not wish to be included in my named report can be included as confidential comments to the editors, which will not be published.

Choose an item.

To further support our reviewers, we have joined with Publons, where you can gain additional credit to further highlight your hard work (see: https://publons.com/journal/530/gigascience). On publication of this paper, your review will be automatically added to Publons, you can then choose whether or not to claim your Publons credit. I understand this statement.

Yes Choose an item.
