# Supplementary material for: Technical workflows for hyperspectral plant image assessment and processing on the greenhouse and laboratory scale
Source: Gigascience. 2020 Aug 20;9(8):giaa090. doi: 10.1093/gigascience/giaa090 (PMC7439585; doi:10.1093/gigascience/giaa090)
Supplement: giaa090_Reviewer_2_Report_Original_Submission — Yufeng Ge -- 4/15/2020 Reviewed [file giaa090_reviewer_2_report_original_submission.pdf]

Reviewer Report

Title: Technical workflows for hyperspectral plant image assessment and processing on the greenhouse and laboratory scale

Version: Original Submission **Date:** 4/15/2020

Reviewer name: Yufeng Ge

Reviewer Comments to Author:

This is a review paper focusing on close-range hyperspectral imaging for plant assessment in the greenhouse and laboratory scales. Given the broad interest of using hyperspectral imaging for plant phenotyping research, as well as the complexity of data structure and analysis method, this manuscript is quite timely and relevant. The hyperspectral image is known for its large data volume. The topic thus is appropriate for the journal. The paper covered the topics including camera and measurement setup, data preprocessing, and data analysis/interpretation. The authors' argument is that a standardized workflow for image acquisition, processing and analysis is needed to make the data comparable among various labs, which is a valid point. The paper provides a good technical summary of hyperspectral imaging (such as camera and imaging stage setup, white referencing), and gives a good compilation of its applications on plant assessment that can be useful for the phenotyping research community. My major comments for the authors to consider improving the manuscript are in the following.

Section of spectral smoothing. The authors only discussed Savitzky-Golay method and missed many other methods that are common for spectral preprocessing. In addition to spectral averaging (binning) that the authors also discussed, other methods like Multiplicative Signal Correction and Standard Normal Variate are also widely used. Other preprocessing such as first and second order derivative are also common. Note Savitzky-Golay can also be used for differentiation. I think you need to mention these methods rather than just Savitzky-Golay.

Preparation for ML. You discussion of calibration set, validation set, and test set are not correct. In machine learning, calibration set is for model calibration (to calibrate model parameters), validation is for model hyper-parameter tuning, and the test set is to evaluate the performance of the developed model. Please make sure you express this correctly. In some implementations, an explicit validation set is not used where model calibration and hyper-parameter tuning are conducted together. In these implementations, test set is also referred to as validation set. I would recommend the authors to read some of the literature on NIRS analysis, as when the images are reduced to the spectrum level, the (pre)processing and analysis share commonalities. There are quite a few publications recently on using VIS-NIR-SWIR for leaf analysis in the context of plant phenotyping. Please study those so you can see calibration/validation schemes and spectral preprocessing.

The explanation following Equation 1 was poor. I cannot understand it. Please revise.

There is significant room for the authors to improve the writing and presentation of the manuscript. There are quite a few places where the wording and phrases can be improved. Please see my comments on the attached document.

Methods

Are the methods appropriate to the aims of the study, are they well described, and are necessary controls included? Choose an item.

Conclusions

Are the conclusions adequately supported by the data shown? Choose an item.

Reporting Standards

Does the manuscript adhere to the journal's guidelines on minimum standards of reporting? Choose an item.

Choose an item.

Statistics

Are you able to assess all statistics in the manuscript, including the appropriateness of statistical tests used? Choose an item.

Quality of Written English

Please indicate the quality of language in the manuscript: Choose an item.

Declaration of Competing Interests

Please complete a declaration of competing interests, considering the following questions:

- Have you in the past five years received reimbursements, fees, funding, or salary from an organisation that may in any way gain or lose financially from the publication of this manuscript, either now or in the future?
- Do you hold any stocks or shares in an organisation that may in any way gain or lose financially from the publication of this manuscript, either now or in the future?
- Do you hold or are you currently applying for any patents relating to the content of the manuscript?
- Have you received reimbursements, fees, funding, or salary from an organization that holds or has applied for patents relating to the content of the manuscript?
- Do you have any other financial competing interests?
- Do you have any non-financial competing interests in relation to this paper?

If you can answer no to all of the above, write 'I declare that I have no competing interests' below. If your reply is yes to any, please give details below.

I declare that I have no competing interests

I agree to the open peer review policy of the journal. I understand that my name will be included on my report to the authors and, if the manuscript is accepted for publication, my named report including any attachments I upload will be posted on the website along with the authors' responses. I agree for my report to be made available under an Open Access Creative Commons CC-BY license (http://creativecommons.org/licenses/by/4.0/). I understand that any comments which I do not wish to be included in my named report can be included as confidential comments to the editors, which will not be published.

Choose an item.

To further support our reviewers, we have joined with Publons, where you can gain additional credit to further highlight your hard work (see: https://publons.com/journal/530/gigascience). On publication of this paper, your review will be automatically added to Publons, you can then choose whether or not to claim your Publons credit. I understand this statement.

Yes Choose an item.
