# Supplementary material for: Technical workflows for hyperspectral plant image assessment and processing on the greenhouse and laboratory scale
Source: Gigascience. 2020 Aug 20;9(8):giaa090. doi: 10.1093/gigascience/giaa090 (PMC7439585; doi:10.1093/gigascience/giaa090)
Supplement: giaa090_Reviewer_3_Report_Original_Submission — Susan Meerdink -- 4/23/2020 Reviewed [file giaa090_reviewer_3_report_original_submission.pdf]

Reviewer Report

Title: Technical workflows for hyperspectral plant image assessment and processing on the greenhouse and laboratory scale

Version: Original Submission **Date:** 4/23/2020

Reviewer name: Susan Meerdink

Reviewer Comments to Author:

This paper presents a workflow for researchers using hyperspectral imaging for phenotyping applications, specifically based in greenhouses and laboratory settings. This paper is very timely and quite necessary, in my opinion. Overall I think the paper is well organized and presents information that will be very useful for researchers as they design their experimental setups. My background is remote sensing, specifically hyperspectral, so many of my comments and suggestions are based on lining up the language in this manuscript with the language used in the existing remote sensing literature base. Since remote sensing researchers have been working with hyperspectral since the 1980s, I believe this will allow readers to find established and published methods that can directly apply to plant phenotyping without having to 'reinvent the wheel'. I am also assuming that most of your readers may not be familiar with hyperspectral. Especially since if I were new to hyperspectral for phenotyping, I would start with reading this paper!
General Comments:
* To be technically correct, use the term hyperspectral instead of spectral. RGB imagery is also spectral, but it just happens to be broadband and only three bands.
* The camera characteristics and measuring setup section should be broken up into two sections. One for camera characteristics and one for the measuring setup. The camera characteristics description is thorough, but I would like to see more details (or more explicitly stated) on the experimental design or measuring setup. Specifically, the authors could elaborate on the following topics:
o As the authors mention, illumination is a significant factor in collecting high-quality data. Not all bulbs will work appropriately - what things do researchers need to know not to have illumination issues? Why should any fluorescent lights be turned off before collection?
o Side-view versus nadir image collection - why would you choose one over the other? Why will side view not translate to outdoor image collections?
o The inclusion of a reference panel (briefly mentioned in a different section) in the scene. Should it be all scenes or a preferred location within a scene?
o A discussion on the field of view of the camera and how to determine camera height based on the sample being collected and desired spatial resolution.
o Pushbroom versus integrating cameras
* There needs to be a better description of each of the remote sensing data levels. At the moment, the terminology isn't quite correct, and the clarity is missing (Radiometric calibration section). Specifically, it would be important to define digital numbers, radiance, and reflectance data levels. They each have very different factors that influence them and require different corrections.

* Do not use the term normalization for reflectance retrieval. Reflectance calibration is ok, but to match the remote sensing literature, reflectance retrieval would be more accurate.

Specific Comments:

Abstract > Results: "This review describes a general workflow for the assessment and the processing of hyperspectral plant data at the greenhouse scale."

I would add greenhouse and laboratory scale since this is the first mention of the measurement scale and it will match the title

Abstract > Conclusions: I would have this start on a new line like Background and Results.

"This publications provides a structured overview on implementing hyperspectral imaging into biological studies."

Publication should be singular. I would also add at the end "at the greenhouse and laboratory scale". This paper would not be useful for outdoor collections with UAV or airborne sensors.

Key Words: Make sure to include hyperspectral.

Key Points: hyperspectral not spectral, needs to be structure for evaluation of what?

"During the last years, spectral sensing of plants has developed as a valuable tool for plant phenotyping [1] [2]."

Rewording - "During recent years, hyperspectral sensing…." I think it is important to say hyperspectral instead of spectral. RGB is also spectral, but it just happens to be broadband.

"The principle of hyperspectral imaging (HSI) is based on the fact that all materials reflect electromagnetic energy in prominent patterns and
specific wavelength due to difference of their chemical composition and inner physical structure [3]. Spectroscopy is defined as the method of acquiring and explaining the spectral characteristics of an object regarding light intensity emerging from molecules at different wavelengths to provide a precise fingerprint of an object."

This sentence needs some rewording. This is not the principle of hyperspectral imaging but remote sensing in general. The difference between hyperspectral and other remote sensing is that hyperspectral is characterized by measuring hundreds of narrow bands in the electromagnetic spectrum. For any remote sensing sensor, the measured signature is the result of a material's chemical composition and inner/outer physical structure. It is important to note that the spectral signature is not just the inner leaf, especially since that depends on the part of the electromagnetic spectrum that is measured. Additionally, it is important to specify HOW hyperspectral is different than multi-spectral sensors (specifically RGB cameras are mentioned). In the paper, a lot of great examples are shown using hyperspectral. Still, I think the introduction could use one sentence saying why someone would invest the extra time/money/effort into using hyperspectral over an RGB camera. Lastly, spectroscopy can also be collected with a point spectrometer instead of an imager. There is a whole literature base that uses point spectroscopy for phenotyping, which is not the focus on this paper. I would add a single sentence acknowledging this difference. Also, it may not be apparent to readers that spectroscopy equals hyperspectral, and I would say that hyperspectral is more commonly used in the plant sciences literature.

"Spectral cameras have become affordable that increase the visible spectrum (400 - 700nm, VIS) of RGB-cameras by the ultra-violet (200 - 400nm, UV,[5]), the near infrared spectrum (700 - 1000nm,NIR, [6]) or even the short wave infrared spectrum (1000 - 2500nm, SWIR, [7])."

This sentence needs rewording. Hyperspectral cameras have become more affordable and as a result, more commonly used? Compared to RGB cameras, they increase the spectral resolution and spectral range?
Reflectance imaging of plants has been related to plant tissue characteristics [9], to detect abiotic stresses [10] or plant diseases [11].
This is the first time the term reflectance is used, and it might be easier for readers who are not familiar with this data type to use hyperspectral instead (until you get a chance to define reflectance in the Data Acquisition and Processing section). This list, as written, suggests these are the only applications of hyperspectral imaging of plants. I would add at the end "among others" to give some flexibility.
"To introduce HSI as a state-of-the-art tool for plant phenotyping a literature overview is presented showing the different biological objectives what hyperspectral sensors are used for in the laboratory and greenhouse scale starting from stress detection and disease classiÂ¬fication to a linking to molecular analysis (QTL analysis) grouped by the introduced level-description."
Suggested rewording - "To introduce HSI as a state-of-the-art tool for plant phenotyping, a literature overview is presented showing the different biological objectives can be achieved with hyperspectral sensors in the laboratory and greenhouse settings including stress detection, disease classiÂ¬fication, and molecular analysis (QTL analysis)."
"The following paragraph introduced introduces techniques to overcome different ..."
Typos: "The following section introduces techniques ..."
"A comprehensive literature review shows examples for hyperspectral application from biotic stress detection like disease or virus detection, abiotic stress detection like heavy metal or cold stress and plant trait extraction like biochemical traits or leaf water content."
Since this is the start of a paragraph, please include again this is at the greenhouse/laboratory scale.
Table 1: Please include in the caption this is for the greenhouse/laboratory scale. I'm not as familiar with hyperspectral greenhouse studies, but there is only one citation for each of these?
"Spectral systems and resulting data differ in the way the camera is calibrated and the data is processed."
These are not the only ways hyperspectral systems differ. As mentioned in the following sections, there are many other factors. Perhaps a more generalized sentence? "Hyperspectral systems and resulting data will vary due to many factors, including camera characteristics, experimental setup, calibration, and data processing.
"… sensor wavelength calibration, the instrument function, the radiometric calibration and spectral and pixel binning."
What is "the instrument function"?
"Four categories of factors that influence the measured spectrum of plants can bedefiÂ¬ned."
Add space between be and defined.
Also, these four factors are HUGE when collecting hyperspectral data and often result in the most errors or incorrectly interpreted data. I love the figure and that these factors are mentioned, but I think they could use a little more elaboration. How might each factor impact your data? The last sentence starts to address this, but in my opinion, it is too much of a summary of all of them. For example, spectra variability due to differences in genotypes is not caused by the optical configuration but the plant's properties.

Camera characteristics and measuring setup
As mentioned in general comments, I believe this section should be split into two, which would allow authors to go into detail about how the measurement setup is critical for high-quality measurements. As I progress through specific comments, I will highlight sections that could be expanded on or moved to the measurement set up section.
"Hyperspectral cameras for plant phenotyping often are line scanners (pushbrooms) as this type of sensor is commonly used in plant science or for high throughput analysis as it, unlike snapshot cameras, provides a very high spatial and spectral resolution."
This sentence is awkward and could use rewording. While they are often line scanners there are other hyperspectral camera systems. Since this is a literature review, mention those scanners and how they are different. In the measurement setup section, the pros/cons of each could be explained.
"The next step, the transfer of these sensor types to the field scale has already been started for tracking the canopy development in cereals [37] or as an open-source and open data project of Terra-Ref [38]."
My remote sensing background has significant issues with this sentence. Hyperspectral data collection has been happening for decades with airborne sensors or point spectrometers for plant applications. Including predicting nitrogen content and canopy development. Since this sentence doesn't add to the camera characteristics section, I would remove it or reword so that it doesn't exclude a whole body of research (which is outside the scope of this paper).
Wavelength calibration: I'm quite confused by this section. The wavelengths that sensor measures should be set by the manufacturer. Are there enough people creating their own hyperspectral sensors for this section to be applicable? In my experience, wavelengths rarely drift, and if they do, the manufacturer would prefer to do the correction. The sentence "The wavelength calibration describes the comparison of measured spectral values with known values [40] and consequently, the mapping of the dispersed geometric access to wavelength in nm." sounds like it is discussing reflectance retrieval, but that is a different section. The sentences "A polynomial Â¬fit of the geometric position of the atomic emission lines on the chip and the known wavelength is conducted. This step is usually performed preliminary by the manufacturer and enables displaying the spectral axis in units of wavelength (nm)." Sounds like you are discussing the conversion of digital numbers to radiance, but that is also another section. I've also never heard the term dispersed geometric access, so it would probably be good to define? Now, it is important to know that each band has a spectral response function (again generally provided by sensor manufacturer or estimate by Gaussian function). This information is critical to resample a camera to another camera spectral resolution.
"Due to differences in quantum efficiency of the detector and varying efficiency of the grating and other optical components (lenses etc.), measurements using different optical systems of the same object under same illumination conditions may not be identical [41]."
A sentence needs to follow this one that spells out to the reader that this data level is called digital numbers. This data-level is influenced by sensor characteristics, atmospheric conditions, and surface properties (in this example plants). This will emphasize the reason why sensors at this data type level are not comparable.
"To correct for such instrument related differences, radiometric calibration of the measurement device or white referencing is needed."
* White reference is NOT used for radiometric calibration. Many software programs will incorporate

the radiance to reflectance step into one which would use the white reference. However, the term white referencing is specifically for converting to reflectance. This is a critical difference when making measurements outdoors, but it worth separating here.

* It is also important to tell the reader what the radiance is, especially since there are many plant applications (such as photosynthetic studies) that require radiance values, not reflectance. This data product is influenced by the light source, atmospheric absorptions, and surface properties, but it does remove camera factors.

* To convert from DNs to radiance, a gain and offset per band are applied to the data which are provided by the sensor designers or engineers. Software provided by the manufacturer should have those values automatically provided. IF they don't, then you have to develop them yourself, which is the description actually provided in this section.

"In many applications absolute radiometric calibration is not required. Often it is sufficient to use a relative spectral calibration to correct for the spectrally varying system efficiency. A simple white referencing and dark subtraction is sufficient for reflectance measurement."

This needs to be reworded. Right now, it jumps from radiometric calibration to a reflectance measurement - which has not been defined. Also, this depends on the camera system. Often it is possible to 'skip' the radiance conversion because it a linear regression with DNs, but this is not the case with every camera (depending on the camera characteristics it can be non-linear spectrally and spatially).

Spectral and spatial binning: Yes, SNR can be increased when data is binned, but many new users will do this incorrectly. For example, many hyperspectral sensors have 'bad bands' towards the upper and lower range of the sensor. Bad bands are those defined with having very high noise and unreliable measurements. These bad bands are lower SNR ratio than other bands because they are at the upper limits of the sensor's capabilities. There can also be bad bands due to atmospheric conditions, which in a greenhouse with high water vapor could be strong. I also feel like this is not necessary for all cameras and really depends on the SNR of the camera used. My suggestion would be to word this as an optional step and explain when a user should consider these methods. Especially since there are sections on dimensionality reduction and spectral smoothing which also impacts the spectral data.

"Thus it can be stated, that a slightly spectral binning will not affect the informative value of the remaining spectrum."

Slightly? I think a different word might more appropriate.

"It includes pre-processing steps where the normalization is performed, the spectral smoothing and 3D correction up to a masking of the object of interest and data splitting, dimension reduction and feature selection for ML."

This sentence is awkward and could use rewording.

Reflectance Calibration: Do not use the term normalization for reflectance retrieval. Define what the reflectance data level is and what the units are. It is important for the readers to know that this data level removes camera effects, atmospheric conditions, and lighting effects, so only the surface properties remain. THIS data source is comparable across camera systems, whereas the other data levels are not.

"Most often highly reflective materials like barium sulfate (SphereOptics.com) act as a reference."

In my opinion, the reference panel is one of the most critical components of making high-quality

hyperspectral measurements. I would love to see this in an experimental setup section with a lot more details. For example, the material does need to be highly reflective but also highly reflective across the entire spectral range of the camera measures. Also, probably the most commonly used panel (but of course more expensive) is a spectralon panel made by Labsphere. White paint for camera measuring 400-1000 nm can also be sufficient.

"Alternatively the use of materials with a known spectral reflectance is established as a standard procedure."

Yes! I always recommend a black, light gray, and dark gray target also. These can be measured with a point spectrometer to get a known reflectance value.

"Performing the object scan right after including the associated dark image, the normalization step can be described by formula 1:"

This formula is the most basic way of converting from radiance to reflectance, using only a single target. In the remote sensing literature, it is referred to as the empirical line correction method. However, if you have a variable atmosphere (such as a greenhouse with fluctuating values) or are covering a large area, a single target may not be sufficient for good data. This is also true if the lighting conditions change or if the data set is a time series. A more advanced empirical line correction method incorporates multiple targets, which can make it robust to these changes. Conversion to reflectance from radiance generally results in the largest data errors, so in my opinion is worth elaborating.

"Based on the assumption that the plant spectrum has a smooth spectrum and peaks within the spectrum are results of outliers and noise the use of soft smoothing algorithms is valid."

This sentence needs to be clarified. Plant spectra can have peaks or valleys that are due to biophysical or structural conditions that people may be interested in. Very sharp peaks that only span one or two wavelengths are definitely noise. This is where a discussion of 'bad bands' that I mentioned before would be useful. Again, this may not apply to all cameras and it may not apply to the whole spectrum depending on the SNR.

"Most established is the Savitzky-Golay smoothing algorithm [49] for hyperspectral data where 15 centered points and a polynomial of degree 3 has shown its applicability [50]."

This is highly dependent on the camera's spectral resolution.

"Literature shows that an the use of erosion as a binary image processing technique is efficient."

Typo shown in italics. There should be a citation with this statement or is it the same as the following sentence? Might be worth mentioning that some machine learning algorithms are robust to them anyway. Also, I'm sure you are aware there is a whole literature for working with mixed pixels which might be helpful for readers to know if their spatial resolutions are coarse.

Preparation for ML: I love that the authors chose to focus on machine learning techniques. I have found too many phenotyping papers that rely on a vegetation index to retrieve their trait of interest. Why do we have cameras that measure hundreds of bands if we are going to reduce them down to one value? I would love to see one sentence on why researchers should use ML approaches rather than a vegetation index.

"To decrease redundancy within the dataset dimension reduction as it can be performed."

I would like to suggest a clarification for this sentence - "Dimensionality reduction methods can decrease spectral redundancy and reduce data volume within the dataset."

"State-of-the-art techniques are principal component analysis (PCA, [55]), feature selection using

recursive feature elimination (RFE), ReliefF or correlation-based feature selection [56]."

I would change "state-of-the-art" to common since PCA was one of the very first dimensionality reduction techniques. Or split it into two sentences - one with common and another with new algorithms.

"If the data is coming from a single plant (trait level 1) the datacube can be used to derive very rough information about the plant like the plant canopy [57]."

Very rough information? What does this mean? Instead of like, I would suggest "such as"

"The correction of thehyperspectral information according to distance and inclination is needed."

Space needed between the and hyperspectral.

"In contrast to SVM or DT approaches, DL is based on N architectures and is based on very huge datasets used for training."

Consider rewording to remove duplicate "is based on"

"DL approaches have been widely used on RGB images for the demands of plant phenotyping as there is a classiÂ¬fication of root tips, shoot and leaves [61] [62] and can be depicted to be state of the art."

Remove there is.

"Usually the results of a classiÂ¬fication are presented by a confusion matrix, which indicates…"

Since the previous sentence said no labeled data was needed, it might be worth mentioning that the confusion matrix does need labeled data.

"Thus, the setup has to tailored has to be tailored towards the size of the plantss."

Remove duplicate s on plants.

"Beside effects of the geometry, like the correlation between normalized difference vegetation index (NDVI) and inclination, have to be taken into account or if possible have to be corrected
[7]."

NDVI is not only influenced by leaf inclination but also more broadly canopy structure.

"When transferring results from the laboratory or greenhouse to the Â¬field the work ow for using HSI is different and has to be designed individually [66]."

I think this paragraph should be condensed significantly since it is definitely out of the scope of the paper and a single paragraph would not be sufficient to explain how this workflow would be transferrable to field settings. The reflectance retrieval process (referred to as normalization here) is completely different for field collections. As mentioned most everything is different. I would summarize by saying "The workflow proposed is not transferrable to field conditions which requires a very different experimental set up to ensure high quality hyperspectral measurements."

"Especially when using high throughput imaging setups [21] combined with hyperspectral cameras periodical imaging leads to huge datasets independent of the scale [37]."

This sentence is should be reworded.

Methods

Are the methods appropriate to the aims of the study, are they well described, and are necessary controls included? Choose an item.

Conclusions

Are the conclusions adequately supported by the data shown? Choose an item.

Reporting Standards

Does the manuscript adhere to the journal's guidelines on minimum standards of reporting? Choose an item.

Choose an item.

Statistics

Are you able to assess all statistics in the manuscript, including the appropriateness of statistical tests used? Choose an item.

Quality of Written English

Please indicate the quality of language in the manuscript: Choose an item.

Declaration of Competing Interests

Please complete a declaration of competing interests, considering the following questions:

- Have you in the past five years received reimbursements, fees, funding, or salary from an organisation that may in any way gain or lose financially from the publication of this manuscript, either now or in the future?
- Do you hold any stocks or shares in an organisation that may in any way gain or lose financially from the publication of this manuscript, either now or in the future?
- Do you hold or are you currently applying for any patents relating to the content of the manuscript?
- Have you received reimbursements, fees, funding, or salary from an organization that holds or has applied for patents relating to the content of the manuscript?
- Do you have any other financial competing interests?
- Do you have any non-financial competing interests in relation to this paper?

If you can answer no to all of the above, write 'I declare that I have no competing interests' below. If your reply is yes to any, please give details below.

I declare that I have no competing interests.

I agree to the open peer review policy of the journal. I understand that my name will be included on my report to the authors and, if the manuscript is accepted for publication, my named report including any attachments I upload will be posted on the website along with the authors' responses. I agree for my report to be made available under an Open Access Creative Commons CC-BY license (http://creativecommons.org/licenses/by/4.0/). I understand that any comments which I do not wish to

be included in my named report can be included as confidential comments to the editors, which will not be published.

Choose an item.

To further support our reviewers, we have joined with Publons, where you can gain additional credit to further highlight your hard work (see: https://publons.com/journal/530/gigascience). On publication of this paper, your review will be automatically added to Publons, you can then choose whether or not to claim your Publons credit. I understand this statement.

Yes Choose an item.
