# Supplementary material for: Technical workflows for hyperspectral plant image assessment and processing on the greenhouse and laboratory scale
Source: Gigascience. 2020 Aug 20;9(8):giaa090. doi: 10.1093/gigascience/giaa090 (PMC7439585; doi:10.1093/gigascience/giaa090)
Supplement: giaa090_Reviewer_3_Report_Revision_1 — Susan Meerdink -- 7/6/2020 Reviewed [file giaa090_reviewer_3_report_revision_1.pdf]

Reviewer Report

Title: Technical workflows for hyperspectral plant image assessment and processing on the greenhouse and laboratory scale

Version: Revision 1 **Date:** 7/6/2020

Reviewer name: Susan Meerdink

Reviewer Comments to Author:

A big thank you to the authors for addressing my concerns and working hard to incorporate terminology commonly used in remote sensing literature. I have a few remaining comments, but they are very minor changes.
"Spectral cameras have become adordable during the last years. Unlike RGB cameras imaging the visible spectrum (400 - 700nm, VIS) ...." I believe you want to saying HYPERspectral here instead.
"Compared to spectroscopy, which measures the same spectral area HSI is...." HSI has not been defined yet.
"For reference panels the options are a permanent reference measuring after each plant if using a box design, referencing within
the measurable volume at the same height as the majority of the plant pixels or a periodical referencing along the scan axis when using a measuring setup at a longer line stage." I like the inclusion of this sentence, but reference panels haven't been discussed yet so the readers may not know what one is. I would suggest adding a sentence saying something like "More information about reference panels can be found in Section X".
"The use of passive light like sunlight which is available outdoors and in greenhouses is valid." I would probably replace valid with preferred. If the sun can be used this should be the first choice. I also think it might be worth mentioning that some greenhouse glass CAN alter the sunlight spectrum if any coatings or special glass is used.
"Often, it is sufficient to use reflectance data rather than radiance data." I would alter this sentence slightly to lead your readers in the right direction. Its not only sufficient, but PREFERRED to use reflectance data especially for vegetation studies. In fact, many of the methods proposed in the ML Sections won't work unless it is reflectance data (as I'm sure you already knew).
"The pre-procesing steps include the normalization, the spectral smoothing and 3D correction, masking of the object of interest, data splitting, dimension reduction and feature selection for ML." Update normalization to be reflectance retrieval.

Methods

Are the methods appropriate to the aims of the study, are they well described, and are necessary controls included? Choose an item.

Conclusions

Are the conclusions adequately supported by the data shown? Choose an item.

Reporting Standards

Does the manuscript adhere to the journal's guidelines on [minimum standards of reporting?] Choose an item.

Choose an item.

Statistics

Are you able to assess all statistics in the manuscript, including the appropriateness of statistical tests used? Choose an item.

Quality of Written English

Please indicate the quality of language in the manuscript: Choose an item.

Declaration of Competing Interests

Please complete a declaration of competing interests, considering the following questions:

- Have you in the past five years received reimbursements, fees, funding, or salary from an organisation that may in any way gain or lose financially from the publication of this manuscript, either now or in the future?
- Do you hold any stocks or shares in an organisation that may in any way gain or lose financially from the publication of this manuscript, either now or in the future?
- Do you hold or are you currently applying for any patents relating to the content of the manuscript?
- Have you received reimbursements, fees, funding, or salary from an organization that holds or has applied for patents relating to the content of the manuscript?
- Do you have any other financial competing interests?
- Do you have any non-financial competing interests in relation to this paper?

If you can answer no to all of the above, write 'I declare that I have no competing interests' below. If your reply is yes to any, please give details below.

I declare that I have no competing interests.

I agree to the open peer review policy of the journal. I understand that my name will be included on my report to the authors and, if the manuscript is accepted for publication, my named report including any attachments I upload will be posted on the website along with the authors' responses. I agree for my report to be made available under an Open Access Creative Commons CC-BY license (http://creativecommons.org/licenses/by/4.0/). I understand that any comments which I do not wish to

be included in my named report can be included as confidential comments to the editors, which will not be published.

Choose an item.

To further support our reviewers, we have joined with Publons, where you can gain additional credit to further highlight your hard work (see: https://publons.com/journal/530/gigascience). On publication of this paper, your review will be automatically added to Publons, you can then choose whether or not to claim your Publons credit. I understand this statement.

Yes Choose an item.
